# Molecular Recognition and Chiral Discrimination from NMR and Multi‐Scale Simulations

**DOI:** 10.1002/chem.202404694

**Published:** 2025-04-02

**Authors:** Tadeu Luiz Gomes Cabral, João Pedro Brussolo da Silva, Claudio Francisco Tormena, Matthias Stein

**Affiliations:** ^1^ Physical Organic Chemistry Lab, Chemistry Institute University of Campinas – UNICAMP Campinas São Paulo 13083–970 Brazil; ^2^ Molecular Simulations and Design Group Max Planck Institute for Dynamics of Complex Technical Systems Magdeburg 39106 Germany

**Keywords:** chirality, diffusion, molecular dynamics, NMR spectroscopy, quantum chemical calculations

## Abstract

Chiral molecules are particularly interesting to the pharmaceutical and agrochemical sectors due to their chemical and physical properties. Separation and identification of enantiomers are critical for a broad range of compounds, and discriminating stereoisomers in solution remains a key analytical challenge. Nuclear Magnetic Resonance (NMR) with Matrix‐Assisted Diffusion‐Ordered Spectroscopy (MAD), in the presence of chiral resolving agents, has emerged as a tool to explore these chiral mixtures. However, insight into the intermolecular interactions that lead to chiral recognition is still limited. Here, we combine experimental MAD studies with computational approaches to investigate the enantioselective discrimination of Mandelic Acid (MA) enantiomers using (*R*)‐BINOL and (*S*)‐BINOL. Molecular dynamics simulations explain the differences in diffusion coefficients for heterochiral complexes. Furthermore, quantum mechanical calculations confirmed enantioselective binding preferences due to differences in Gibbs free energies, highlighting the fundamental interactions and structural criteria that explain the NMR shielding and the diffusion trends. This integrated approach bridges experimental and theoretical studies, offering a comprehensive understanding of chiral recognition mechanisms and elucidating the observed heterochiral preference of BINOL for MA enantiomers. Our findings advance the field of chiral analysis and lay a foundation for future developments for identifying stereoisomers and recognition modes underlying enantioselective binding.

## Graphical abstract

This study explores the enantioselective discrimination of mandelic acid enantiomers using (*R*)‐BINOL and (*S*)‐BINOL through a combination of nuclear magnetic resonance experiments, molecular dynamics simulations, and quantum mechanical calculations. The findings provide a detailed understanding of the diffusion properties and binding interactions that drive chiral recognition at the molecular level.

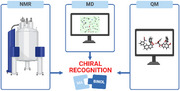

## 1 Introduction

Chiral molecules are widely applied in the pharmaceutical industry,^[^ ^1^ ^]^ agrochemical sector,^[^ ^2^ ^]^ food science,^[^ ^3^ ^]^ and fragrance industries.^[^ ^4^ ^]^ Enantiomers are non‐superimposable mirror images with identical physical and chemical properties. However, these compounds behave differently when interacting with polarized light or other chiral entities.^[^ ^5^, ^6^ ^]^ In the latter case, distinct non‐covalent interactions may result in different chemical and biological activities for each stereoisomer. For instance, one enantiomer of a drug may exhibit the desired therapeutic effect, while the other stereoisomer may be inactive or even potentially harmful.^[^ ^7^ ^]^ As a result, the U.S. Food and Drug Administration (FDA)^[^ ^8^ ^]^ and the European Medicines Agency (EMA)^[^ ^9^ ^]^ established specific regulations and guidelines for the development, approval, and marketing of chiral drugs. These directives require that each enantiomer, before entering the market, must be properly characterized and its properties thoroughly examined, ensuring the safety and efficacy of the chiral pharmaceuticals. Consequently, the control, analysis, and characterization of each isomer in a racemic mixture are critical for drug development. However, the chiral resolution is still challenging for experiments and theoretical research.^[^ ^6^, ^10^ ^]^

A range of advanced experimental techniques, such as chiral chromatography,^[^ ^11^, ^12^ ^]^ X‐ray powder diffraction,^[^ ^13^ ^]^ and circular dichroism,^[^ ^14^ ^]^ are employed to examine and identify enantiomers. Among these, nuclear magnetic resonance (NMR) spectroscopy has emerged as a particularly advantageous and convenient tool to characterize chiral molecules.^[^ ^15^, ^16^ ^]^ In NMR, enantiomeric mixtures typically produce superimposed signals, requiring the addition of a chiral resolving agent (CRA) to separate the overlapping chemical shifts, distinguish between the two stereoisomers, and then quantify the enantiomeric excess (*ee*).^[^ ^17^, ^18^ ^]^ Recently, the idea of combining CRAs with diffusion‐NMR experiments (DOSY), known as the chiral matrix‐assisted DOSY (MAD) approach, was introduced.^[^ ^19^, ^20^, ^21^ ^]^ This innovative strategy extends beyond merely separating spectral peaks of enantiomers or determining the enantiomeric excess *ee*; it also offers additional insights into the physical chemistry behind the enantiodiscrimination process, including detailed information about the stereoselectivity of CRAs. This additional knowledge is essential for the determination of the absolute configuration of chiral entities, a significant challenge faced by the NMR community.

**Figure 1:**
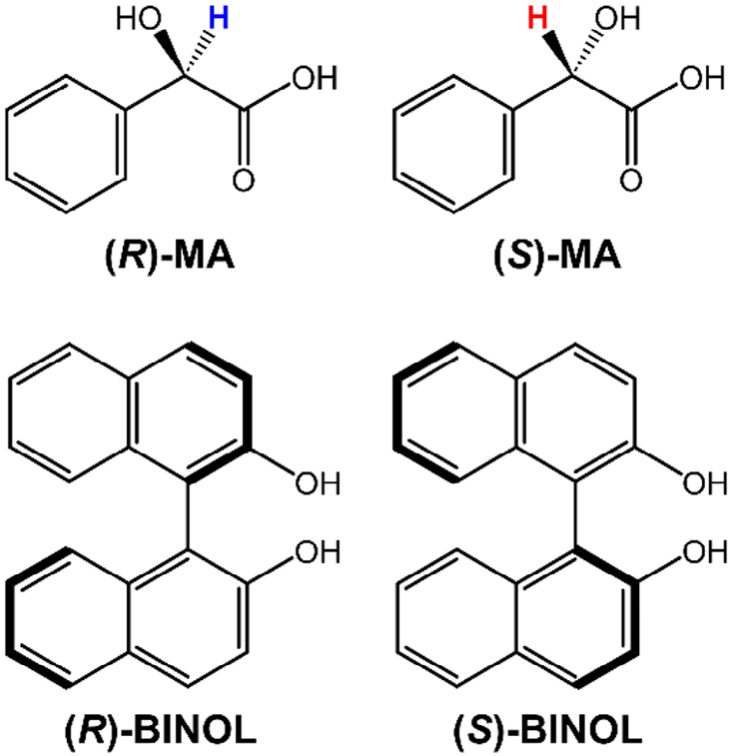
Chemical structures of the investigated mandelic acid (MA) enantiomers: (*R*)‐MA and (*S*)‐MA, and the chiral resolving agents: (*R*)‐BINOL and (*S*)‐BINOL. The highlighted atoms indicate the hydrogen atoms at the chiral center monitored in this study.

The concept of MAD relies on the formation of distinct diastereomeric complexes between the CRA and each enantiomer, which alter the enantiomer's chemical environment and diffusion properties. This enables chiral discrimination and offers insights into the complex dynamics and the enantioselectivity of the resolving agent.^[^ ^22^, ^23^ ^]^ The viability of the MAD strategy was demonstrated by Salome and Tormena^[^ ^20^ ^]^ who employed (*R*,*S*)‐BINOL and its derivatives as CRAs to discriminate stereoisomers of 17 chiral compounds. While their study established the feasibility of achieving enantiodifferentiation by MAD and described the influence of various experimental conditions on discrimination, it was unable to accurately assess the stereoselectivity of the CRAs for certain analytes. An example of this limitation is the application of (*R,S*)‐BINOL to discriminate a racemic mixture of Mandelic Acid (MA) enantiomers (Figure 1), a key prototypical chiral molecule in industrial synthesis, cosmetics, and pharmaceuticals.^[^ ^24^ ^]^ Therefore, the study was not fully conclusive due to a lack of information about the enantiodiscriminative recognition of (*R,S*)‐BINOL, stemming from unresolved signals and unresolved diffusional characteristics for each MA enantiomer.

Computational approaches, including molecular dynamics (MD) simulations and density functional theory (DFT) calculations, are essential tools for elucidating chiral complexation phenomena.^[^ ^25^, ^26^ ^]^ Theoretical approaches can provide a detailed atomic‐level resolution of chiral interactions, their thermodynamics, and the behavior of diastereomeric complexes over time, thereby elucidating chiral recognition processes beyond experimental observations.^[^ ^27^, ^28^, ^29^ ^]^ These computational methods are also utilized to investigate interactions between solvents and chiral compounds,^[^ ^30^ ^]^ analyze chiral separations,^[^ ^31^ ^]^ and explore various enantioselectors.^[^ ^32^, ^33^, ^34^ ^]^ Although the literature includes examples of computational studies on chiral systems that were experimentally characterized using NMR, many of these studies focus primarily on interactions between enantiomers and specific resolving agents, such as cyclodextrins or metal complexes.^[^ ^35^, ^36^ ^]^ Computer simulations to assign the absolute configuration by NMR were mostly focusing on alignment media instead of the isotropic solution state.^[^ ^37^ ^]^ Assignment and resolution of enantioselective differences in the chiral complexation process involving MA enantiomers and BINOL still remain unresolved.

Here, we present a novel and systematic framework for understanding the chiral recognition modes between (*R*)‐MA and (*S*)‐MA with (*R*)‐BINOL and (*S*)‐BINOL at the molecular level. We combine experimental and computational approaches to investigate the details of molecular binding recognition that are driving the enantioselective discrimination as observed in diffusion‐NMR. Extensive molecular dynamics simulations were performed to calculate the diffusion properties and analyze the temporal evolution of diastereomeric complexes. Quantum mechanical studies were used to refine the binding free energies and interaction modes. Calculated ^1^H‐NMR shifts are in excellent agreement with experimental spectra and allow the identification of relevant binding modes. Our approach provides good agreement between computed and experimental enantioselective recognition modes, demonstrating the power of this complementary strategy in unraveling the molecular‐level principles of chiral recognition. It thus contributes significantly to the ongoing development of methodologies for stereochemical analysis.

## 2 Results and Discussion

### 2.1 Chiral Discrimination from NMR Studies

**Figure 2:**
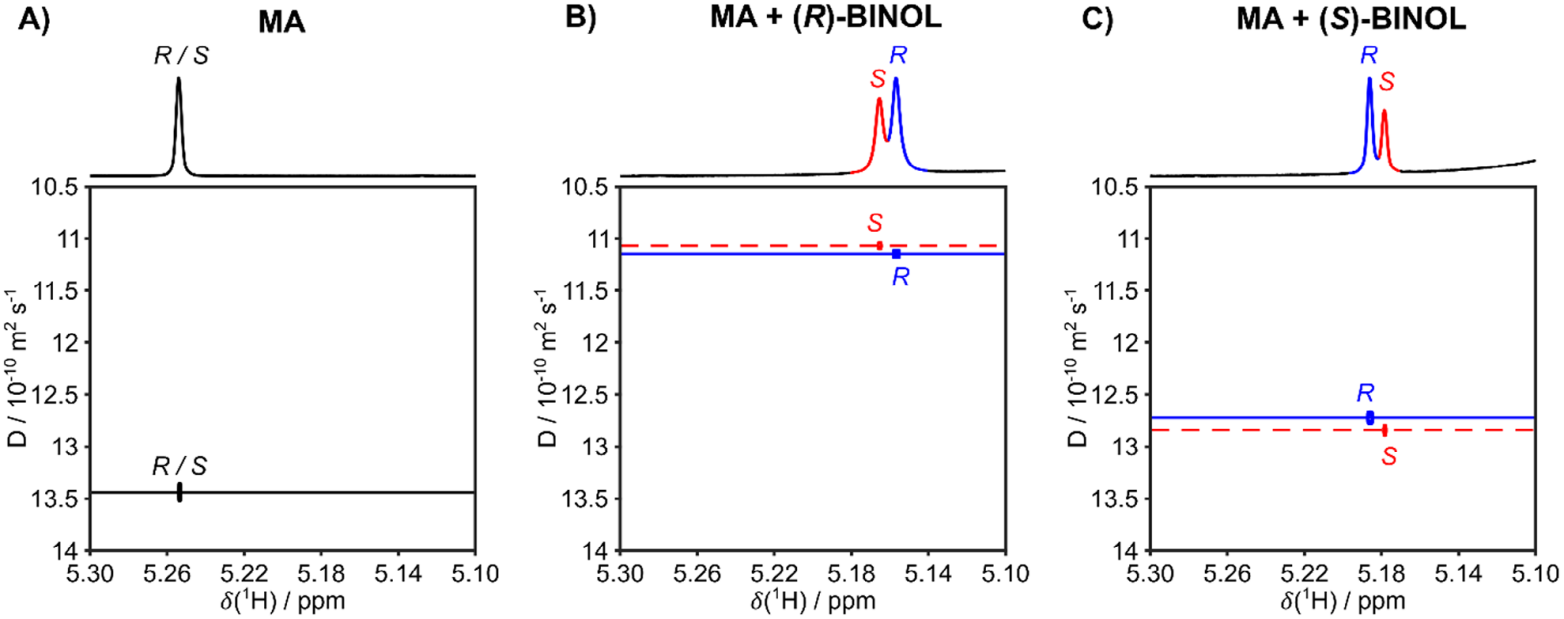
600 MHz ^1^H DOSY with the least attenuated 1D spectrum displayed at the top for an A) enantiomeric mixture of (*R*)‐ and (*S*)‐Mandelic Acid (MA), B) (*R,S*)‐MA with (*R*)‐BINOL, and C) (*R,S*)‐MA with (*S*)‐BINOL. The mixture contains 70% (*R*)‐MA and 30% (*S*)‐MA, with deuterated chloroform as the solvent.

Salome and Tormena^[^ ^20^ ^]^ initially explored the experimental differentiation of MA enantiomers binding to (*R*)‐BINOL and (*S*)‐BINOL as CRAs. However, the molecular recognition modes could not be resolved due to the racemic nature of their sample. To address this limitation, we investigated a chiral system using a non‐racemic mixture of 70% (*R*)‐MA and 30% (*S*)‐MA. The enriched presence of the *R*‐enantiomer in the sample allowed a precise frequency assignment and, consequently, assignment of diffusion coefficients for each individual stereoisomer (Figure 2), thus clarifying the stereoselective recognition of the (*R*,*S*)‐BINOL CRA. The experiments were also performed for mixtures of 50/50 and 30/70 (*R*)‐MA/(*S*)‐MA. The results are consistent and rule out an effect of concentration on the diffusional properties (see below).

As depicted in Figure 2A, the MA enantiomeric mixture, in the absence of a CRA, shows identical chemical shifts and diffusion constants. Upon the addition of BINOL, the MA stereoisomers become distinguishable and separable due to the formation of distinct diastereoisomeric complexes. These complexes are responsible for the discrimination of enantiomers since they result from differences in intermolecular interactions between each MA enantiomer and BINOL.

This difference in chemical environment is further reflected by two specific issues, which depend on the stereochemistry of the employed CRA. For instance, when (*R*)‐BINOL was applied as a resolving agent (Figure 2B), the signal with higher intensity, (*R*)‐MA, exhibited a lower chemical shift. This suggests that the hydrogen atom at the chiral carbon center of (*R*)‐MA experiences a larger shielding chemical environment than the corresponding hydrogen atom of (*S*)‐MA in the presence of (*R*)‐BINOL.

On the opposite side, when (*S*)‐BINOL was employed (Figure 2C), (S)‐MA showed a lower frequency than (*R*)‐MA, suggesting a larger shielding effect for the (*S*)‐enantiomer. Thus, homochiral complexes show larger shielding for the hydrogen atom at the chiral center than the heterochiral complexes.

The diffusion dimension also follows the two distinct trends depending on the chirality of the resolving agent (see Figure 2). In the presence of (*R*)‐BINOL, the heterochiral (*S*)‐MA complex exhibited a lower diffusion coefficient, while (*R*)‐MA showed a lower diffusion coefficient in complex with (*S*)‐BINOL. A comparison of the diffusion behavior for both BINOL enantiomers revealed that (*R*)‐CRA was responsible a larger reduction in the (*R,S*)‐MA diffusion coefficients than the (*S*)‐resolving agent. This suggests that for (*R*)‐BINOL, the complexation is more favorable, and the equilibrium is slightly shifted toward the formation of the diastereomeric complex compared to (*S*)‐BINOL. However, the (S)‐resolving agent induces a greater distinctiveness (larger difference in diffusion coefficient) for complexes of CRA and (*R*,*S*)‐MA enantiomers, demonstrating a higher stereoselectivity compared to the (*R*)‐agent. Table 1 gives the measured diffusion coefficients and the differences Δ*D* between homo‐ and heterochiral diastereomeric complexes.

**Table 1:** Experimental and calculated diffusion coefficients (*D* ^exp.^ and *D* ^calc.^) for each (*R,S*)‐MA enantiomer in the presence of (*R*)‐BINOL or (*S*)‐BINOL.

Resolving agent	Enantiomer	*D* ^exp.^ ± error	Δ*D* ^exp.^ ± error	*D* ^calc.^ ± error	Δ*D* ^calc.^ ± error
(*R*)‐BINOL	(*R*)‐MA	11.15 ± 0.02	+0.07 ± 0.03	10.29 ± 0.18	+2.26 ± 0.18
	(*S*)‐MA	11.08 ± 0.02		8.03 ± 0.02	
(*S*)‐BINOL	(*R*)‐MA	12.73 ± 0.03	−0.11 ± 0.04	6.79 ± 1.56	−3.43 ± 1.85
	(*S*)‐MA	12.84 ± 0.03		10.22 ± 0.99	

These differences in frequency and diffusion coefficients also arise from the varying lifetimes of the diastereomeric complexes, which result from the strength of the interactions of BINOL with each MA enantiomer. The longer the enantiomer remains in complex with the chiral agent (longer lifetime), the larger the reduction of diffusion coefficient with respect to the corresponding counter stereoisomer. The enantiomer with the lower diffusion coefficient thus forms the most stable diastereomeric complex with the CRA, thereby indicating BINOL's enantioselective binding for a particular stereoisomer. The diffusional analysis indicates a heterochiral selectivity between BINOL and MA, i.e., (*R*)‐CRA interacts more tightly with the (*S*)‐MA enantiomer and *vice versa*.

The same enantiodiscriminative pattern observed for mixtures of 70/30 of (*R*)‐MA/*(S)*‐MA can also be detected in racemic mixtures 50/50 and mixtures with 30/70, albeit to a slightly different extent (see Section S3.2, Supporting Information). This clearly demonstrates the reproducibility of the difference of enantiodiscrimative diffusion coefficient differences, independent from an enantiomeric excess in solution. Previous studies^[^ ^19^, ^38^, ^39^ ^]^ on similar chiral systems with known enantiomeric excess, also suggested that enantioseparation tendencies (in the frequency and diffusion dimension) generally remain consistent across different enantiomeric ratios. This indicates that the enantiomeric composition may not significantly influence the stereoselectivity of the resolving agent.

Although diffusion differences are observed across varying enantiomeric compositions, these represent moderate enantiodiscriminations (small differences), which is an expected outcome due to the chemical similarity of the analytes. Enhancing this separation with new resolving agents is a current analytical challenge that is beyond the scope of this work and was addressed in our previous experimental studies.^[^ ^19^ ^]^

Furthermore, the diffusion coefficients (*D*) and enantiodifferences in the diffusion domain (Δ*D*_R,S_) exhibit only slight variations when comparing the results from different enantiomeric compositions. This is a consequence of changes in the equilibrium rate between the free and complex entities. However, the differences between enantiomer diffusion coefficients, Δ*D*_RS_ = *D*_R_ − *D*_S_, remain consistent and exceed the estimated experimental error (Table 1 and Table S3, Supporting Information). This reinforces the importance of focusing on relative trends rather than absolute values when analyzing chiral interactions through diffusion‐NMR experiments. To rule out interactions between mandelic acid molecules as the cause of the observed diffusion asymmetry, additional Nuclear Overhauser Effect Spectroscopy (NOESY) experiments were performed. In the recorded 2D NOESY spectra, no correlation between the MA and BINOL signals was observed (see Section S3.3, Supporting Information). However, the lack of correlation does not necessarily demonstrate the absence of intermolecular interactions between the two compounds. In small molecules, a small spin diffusion effect results in the intermolecular homonuclear NOE being less pronounced than intramolecular NOE. Consequently, the cross‐relaxation process during the NOE experiment was not able to detect the correlation between the signals of MA and CRA, even with varying mixing times.

However, there is sufficient evidence from the diffusion experiments to support the presence of intermolecular interactions that demonstrate the chiral recognition between MA and BINOL.

### 2.2 Enantiodiscrimination from Calculated Diffusion Coefficients and Complex Formation

Despite the clear experimental observation of (*R,S*)‐MA and (*R,S*)‐BINOL enantioselective discrimination, the underlying determinants of the chiral recognition interactions remain unresolved. This motivated us to perform extensive molecular dynamics (MD) simulations. The primary focus was to computationally reproduce the differences in diffusion‐NMR profiles. Molecular diffusion refers to the displacement and translational random motion of molecules in solution. Accurately simulating this motion requires a large number of MD simulations and an averaging of results from the individual trajectories.^[^ ^40^, ^41^ ^]^ The accuracy of the calculated diffusion coefficient also depends on the number of molecules (system size), the choice of force fields, and the simulation time for each replicate.^[^ ^40^, ^41^, ^42^, ^43^, ^44^, ^45^ ^]^

**Figure 3:**
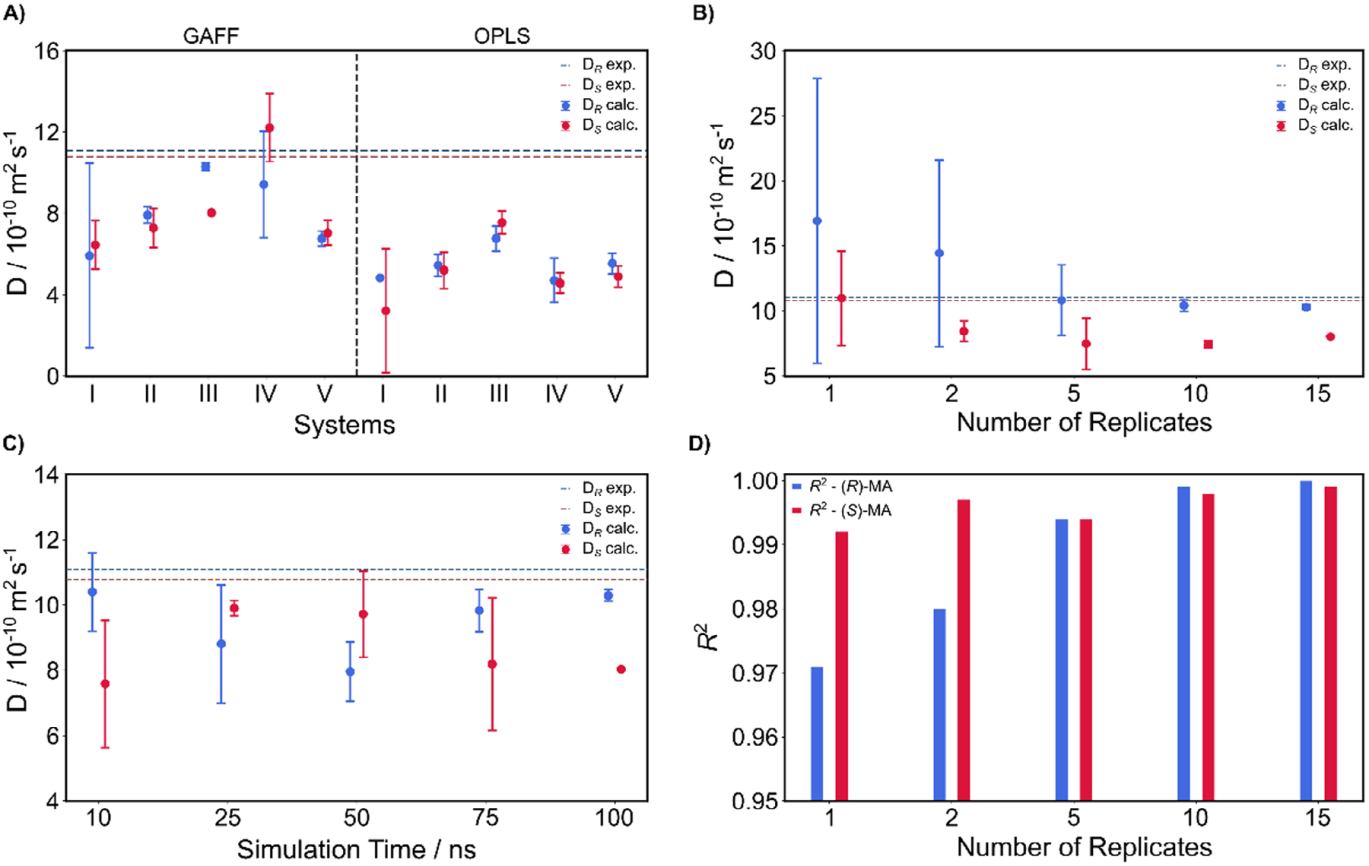
Effect of MD system setup on calculated diffusion coefficients of MA enantiomers in the presence of (*R*)‐BINOL. A) Calculated diffusion coefficients using GAFF and OPLS force fields and comparison with experiment for various concentrations: I: 1 (*R*)‐MA and 1 (*S*)‐MA/10 (*R*)‐BINOL/935 CHCl_3_; II: 5 (*R*)‐MA and 5 (*S*)‐MA/50 (*R*)‐BINOL/2338 CHCl_3_; III: same composition as I but 5 solute copies; IV: 1 (*R*)‐MA or 1 (*S*)‐MA/10 (*R*)‐BINOL/468 CHCl_3_; V: 5 (*R*)‐MA or 5 (*S*)‐MA/25 (*R*)‐BINOL/1169 CHCl_3_. Systems IV and V are individual simulations for one enantiomer of MA. B) Calculated diffusion coefficients using the GAFF force field for different numbers of replicates. C) Calculated diffusion coefficients and their statistical errors as a function of simulation time for 15 replicates. D) *R^2^* correlation coefficients for linear fitting of mean square displacement (MSD) as a function of the number of 100 ns replicates.

To determine the appropriate concentration, suitable force field, and required simulation time for accurate calculations of diffusion coefficients, we extensively benchmarked these parameters. The key findings for appropriate molecular dynamics (MD) settings to model the dynamics of the MA enantiomers in the presence of a resolving agent are depicted in Figure 3.

Figure 3A illustrates the effect of varying solvent concentrations and force fields (GAFF and OPLS) on the calculated diffusion coefficients for MA enantiomers in the presence of BINOL. Overall, the GAFF force field exhibited smaller deviations from experimental values compared to the OPLS force field. The results also demonstrate that simulations with 5 solute molecules provide reliable and statistically relevant data. Additionally, maintaining concentrations closer to the experimental conditions, ≈468 CHCl_3_ molecules per MA molecule (System III in Figure 3A), further enhances the accuracy of calculated diffusion coefficients. Another important observation is that both (*R*)‐enantiomer and (*S*)‐enantiomers must be simulated simultaneously to capture enantiomeric differences in diffusion properties. When each enantiomer is simulated separately (systems IV and V in Figure 3A), the calculated diffusion coefficients are almost indistinguishable and do not exceed the error margin. This demonstrates that the observed enantioselectivity results from a competitive interaction between the MA enantiomers and the CRA. Distinct binding modes and complex’ lifetimes are observable only when both enantiomers are present.

After optimizing the concentration, we investigated the impact of simulation time on the diffusional behavior. As shown in Figure 3C, a simulation time of 100 ns is necessary to capture diffusion trends accurately with a small statistical error. Although shorter simulations yield diffusion coefficients in a similar range to those at 100 ns, they are unsuccessful in reproducing the experimental order of enantiomeric diffusion coefficient differences between the MA stereoisomers in the presence of BINOL. Thus, longer simulation times are required to properly sample the enantiomeric separation and diffusion profiles, particularly for small chiral molecules. The overestimated diffusion coefficient differences between (*R*)‐MA and (*S*)‐MA in the presence of (*R*)‐BINOL demonstrate the challenges in accurately predicting diffusion coefficients for small organic solutes in organic solvents. These might stem from the complexity of accurately modeling solute–solute, solvent–solvent, and especially solute–solvent interactions with classical and non‐polarizable forcefields. Such limitations (as reported in the literature and our studies) often result in underestimated diffusion coefficients for these types of systems (here for the heterochiral complexes).

Typically, longer simulation times reduce the need for a large number of replicates.^[^ ^40^, ^41^, ^42^, ^43^ ^]^ However, due to the lack of previous studies on the diffusion properties of these chiral systems, we also evaluated the optimal number of replicates that allow an enantiodiscrimination of the MA enantiomers. Figure 3B shows the convergence of calculated diffusion coefficients plus their errors. The correlation coefficient (*R^2^*) is given in Figure 3D for linear fitting of the mean square displacement (MSD) as a function of the number of replicates. With increasing number of replicates, the calculated diffusion coefficients and enantiomeric differences become distinguishable and more reliable. This is due to the improved description of molecular diffusion and the linearity of the mean MSD from 15 replicates. As shown in Figure 3D, *R* ^2^ exceeds 0.990 when the number of replicates increases, indicating a linear MSD regime. In our study, 15 replicates were sufficient to accurately describe the diffusional behavior of MA enantiomers in presence of a CRA. The time evolution of the MSD for each trajectory with different force fields, system composition, number of replicates, and their linear fits are given in pages S12–S28, Supporting Information.

With the optimized system setup and force field parameters, (*R*)‐MA and (*S*)‐MA enantiomers in the presence of (*R*)‐BINOL or (*S*)‐BINOL as resolving agents were simulated. The simulations were conducted by maintaining the ratios of MA enantiomers and BINOL close to the concentrations with optimal experimental separations.^[^ ^20^ ^]^

**Figure 4:**
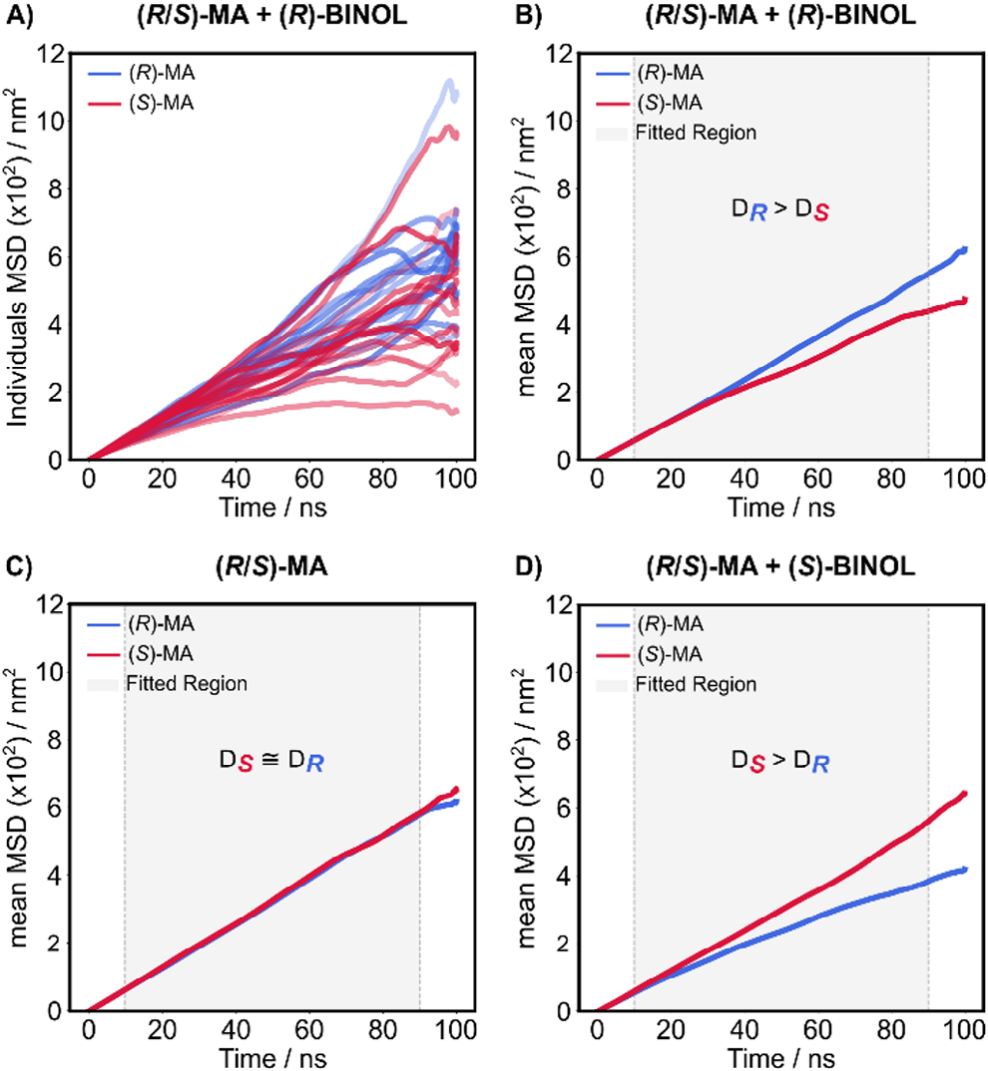
A) Mean square displacement (MSD) over time for (*R*)‐MA (blue) and (*S*)‐MA (red) in the presence of (*R*)‐BINOL from 15 independent MD simulations of 100 ns each. B) MSD from averaged displacements of individual trajectories in the presence of (*R*)‐BINOL., C) diffusion of MA enantiomers in the absence, and D) in the presence of (*S*)‐BINOL. The gray area indicates the region used for linear fitting to obtain the diffusion coefficients.

Figure 4A shows the MSD profiles for the individual MD trajectories for (*R*,*S*)‐MA in the presence of (*R*)‐BINOL. They are clearly non‐linear in time and do not accurately reflect the molecular diffusion behavior. However, a more accurate representation of diffusional motion emerges by averaging over all trajectories (Figure 4B–D). This average sufficiently describes the linear regime, allowing us to apply the Einstein relation to derive diffusion coefficients.^[^ ^40^, ^41^, ^42^, ^43^ ^]^ In the absence of any CRA (Figure 4C), the MA enantiomers exhibit almost identical MSDs and indistinguishable diffusion coefficients. Figure 4B,D illustrates that the heterochiral BINOL complexes exhibit lower diffusion coefficients. In the case of (*R*,*S*)‐MA in the presence of (*R*)‐BINOL (Figure 4B), the simulations show that the diffusion coefficient for (*S*)‐MA decreases more (is lower) than for (*R*)‐MA. In the presence of (*S*)‐BINOL (Figure 4D), (R)‐MA displays a lower diffusion coefficient than (*S*)‐MA. In the simulations, the heterochiral complex with lower diffusion coefficient is slightly underestimated, while the homochiral with a higher coefficient is in excellent agreement with the experiment (Table 1).

The averaged MSD of heterochiral complexes over time shows a reduced linearity over time compared to the homochiral complexes. However, inspection of the *R* ^2^ values reveals that, for both CRAs, the values are 0.999 for (*R*)‐BINOL and 0.996 for (*S*)‐BINOL, indicating a strong correlation and a close to linear profile for the average MSD plot of both enantiomers. A deviation from linearity might originate from the formation of the diastereomeric complexes between MA and BINOL and their different intermolecular interactions or complex’ lifetimes. These differences in the complexation process would vary between homo‐ and heterochiral complexes and affect the random motion of the diastereomeric complexes to different degrees.

Overall, our molecular simulations are able to reproduce the experimentally observed differences in diffusion coefficients for MA enantiomers. This gives confidence in the choice of setup and force field reliability for the calculated diffusion constants from MD data. By comparing the calculated values with experimental data (Table 1), it is clear that the MD simulations qualitatively reproduce the diffusion coefficient trends. Upon the addition of (*R*,*S*)‐BINOL, the diffusion coefficients of both MA enantiomers decrease, indicating that both stereoisomers begin to interact with the CRA and form diastereomeric complexes with the heterochiral complexes displaying lower diffusion constants. The diffusion coefficients of all independent simulations, their statistical error, and the linear correlation coefficient for each independent trajectory for (*R*)‐BINOL and (*S*)‐BINOL can be found in pages S28–S31, Supporting Information).

As shown in Table 1, our approach is able to reproduce the correct signs (positive or negative) for differences in diffusion profiles Δ*D*. Additionally, it can reproduce the relative ratios between the Δ*D* values in the presence of each resolving agent. Experimentally, the absolute ratio of separation (ΔD_(_ *_S_*_)‐BINOL_/ΔD_(_ *_R_*_)‐BINOL_) by (*S*)‐BINOL to that by (*R*)‐BINOL is ≈1.57 ± 0.88 (i.e., (*S*)‐BINOL exhibits a 1.57 ± 0.88 times higher discrimination of MA enantiomers compared to (*R*)‐BINOL)), while the computed value is 1.52 ± 0.83. Although the Δ*D* values for each CRA are similar (when considering experimental error), the agreement in the relative ratios suggests that our approach accurately captures the chiral separation effect induced by both resolving agents.

Such an agreement with only small deviations from experimental is a challenging task due to the mutual dependence of dynamic properties (such as diffusion coefficients) on various experimental and computational conditions and parameters. For instance, the NMR diffusion coefficients are susceptible to changes depending on the data acquisition conditions and also data processing. Very often, interpretations rely on qualitative trends and relative differences between the diffusion coefficients rather than absolute numerical values. Here, our simulations can actually reproduce the trends of experimental observations and pave the way for a more detailed investigation of the chiral recognition modes.

In our investigated system, the change in diffusion coefficients is more pronounced for heterochiral complexes. This indicates that the formation of more stable heterochiral diastereomeric complexes is responsible for the reduction of diffusion coefficients. It is plausible to infer that the stability of these complexes is related to their lifetimes. Table 2 supports this assumption by presenting the calculated mean residence time of chiral complexes for each MA with each BINOL stereoisomer, i.e., the diastereoisomeric complex lifetimes. Details and results are given in pages S31–S33, Supporting Information).

**Table 2:** Calculated average complex lifetimes with error for (*R*,*S*)‐BINOL interacting with (*R*,*S*)‐MA in ns.

Complex	Lifetime	error
(*R*)‐MA + (R)‐BIN	84.46	0.38
(*S*)‐MA + (R)‐BIN	86.04	0.46
(*R*)‐MA + (*S*)‐BIN	85.48	0.39
(*S*)‐MA + (*S*)‐BIN	83.11	0.49

From Table 2, it becomes apparent that, indeed, the heterochiral complexes between MA and BINOL (*RS* or *SR*) have a longer lifetime than homochiral complexes (*RR* or *SS*). In the presence of (*R*)‐BINOL, the complexes with (*S*)‐MA interact for 1.58 ± 0.60 ns longer than with (*R*)‐MA, suggesting that these complexes are more stable. In the (*S*)‐BINOL case, the lifetime difference is even larger (by 2.37 ± 0.63 ns). This is fully consistent with the diffusional coefficient differences (see above). For (*S*)‐BINOL, marginally larger lifetime differences can be seen, which might imply a slightly more pronounced chiral separation. This is also seen in experimental and computational diffusion coefficient differences, where (*S*)‐BINOL exhibits a slightly more pronounced effect in stereoisomer separation (in terms of relative ratios).

**Figure 5:**
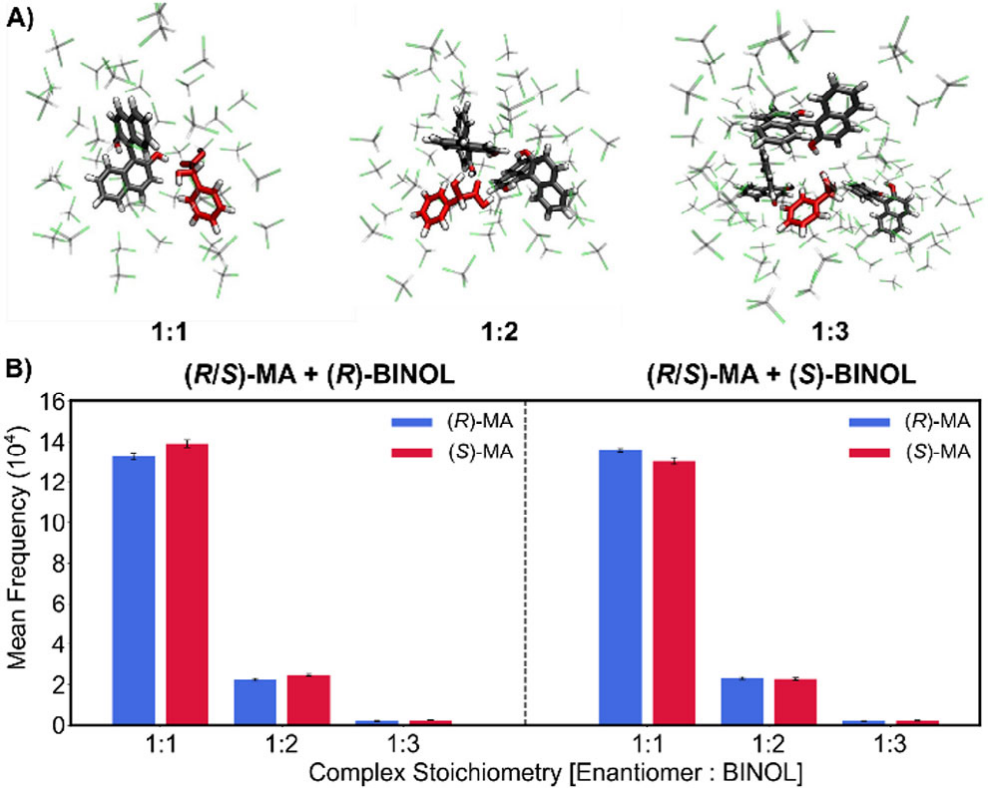
A) Representative molecular structures for complex formation between MA and BINOL with different stoichiometry. B) Mean frequency of complex formation between MA enantiomers in the presence of (*R*)‐BINOL (left) and (*S*)‐BINOL with different stoichiometry.

In order to further understand the factors that influence the observed lifetime and stability differences, we investigated the probability of formation of MA‐BINOL chiral complexes with different stoichiometry. This information is summarized in Figure 5, showing the average occurrence of complexes. Detailed numerical results can be found in pages S33–S35, Supporting Information). The formation of 1:1 complexes is clearly dominating, and 1:2, 1:3, and higher complex numbers are very rarely observed. Complexes with even larger stoichiometry are statistically not relevant.

This already rationalizes the enantioselective complexation and suggests that the 1:1 complex is the dominant species in solution. It also explains the observed enantiodifferences of the formed diastereomeric complexes over the simulation time. For (*R*)‐BINOL, the longer lifetime of the (*S*)‐enantiomer complex leads to a higher number of complexes compared to the (*R*)‐analyte. The excess number of heterochiral 1:1 complexes is 6429 ± 2521 for (*R*)‐BINOL and 5565 ± 1797 for (*S*)‐BINOL, respectively (see Tables S31 and S32, Supporting Information).

It is important to note that the MA enantiomers, in principle, can also self‐associate in solution. However, MA–MA interactions apparently do not contribute to the differentiation of chemical shifts or diffusion coefficients in the absence of BINOL. Since this is not relevant to the chiral recognition process, MA–MA complex formation was not considered in our analysis.

**Figure 6:**
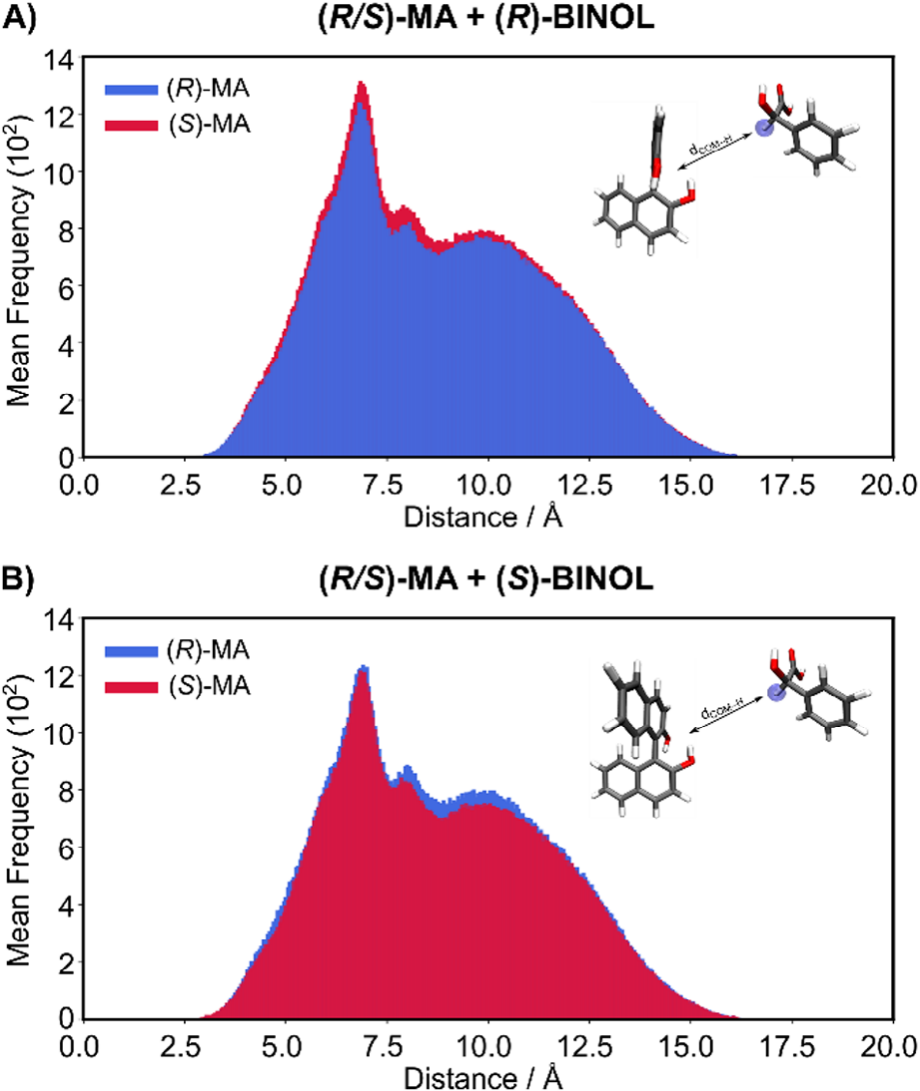
Histograms illustrating the mean frequency distribution of the distances between the hydrogen atom at the chiral center of MA enantiomers and the center of mass of A) (*R*)‐BINOL and B) (*S*)‐BINOL in a 1:1 complex.

Additional structural analysis on the dominating 1:1 complexes was conducted to correlate the NMR frequency with MD results and to elucidate the correlation between the enantiomer with the lower diffusion coefficient and higher NMR shielding. We calculated the distances between the discriminating hydrogen atom of (*R*,*S*)‐MA and the center of mass (COM) of the BINOL for each 1:1 complex from the MD (Figure 6). This approach provided insights into the chemical environment of each enantiomer in the presence of the CRA.

Figure 6 illustrates that the complexation behavior for (*R*)‐BINOL is the mirror image of (*S*)‐BINOL. The largest number of complexes are formed at H_MA_···BINOL_COM_ distances between 5.0 and 7.5 Å, where non‐covalent interactions such as van der Waals, hydrogen bonding, and π─π stacking are feasible in both homochiral and heterochiral complexes. Within 4.0 to 12.5 Å, the heterochiral complexes exhibit a higher number of contacts than homochiral complexes. In contrast, at shorter distances, homochiral occurs slightly more frequently than heterochiral complexation. For distances larger than 12.5 Å, the chiral selection is absent since intermolecular interactions are short‐range and susceptible to solvent effects, resulting in a lower differentiation between enantiomers.

These intermolecular distances between 2.5 to 12.5 Å are crucial for distinguishing between homo‐ and heterochiral 1:1 complexes. In general, at longer distances, where heterochiral complexes dominate, the discriminating hydrogen atom is farther from the BINOL. At shorter distances, where homochiral complexes are dominating, this hydrogen atom is closer to the CRA. In the homochiral complex at a short distance, the hydrogen atom could be more shielded by the electronic density of BINOL compared to the other diastereomeric complexes due to their shorter intermolecular distances.

This may be one reason for the lower chemical shift in the NMR for homochiral complexes with BINOL (see Figure 2). These results are also consistent with the diffusion profiles (see above), where heterochiral complexes exhibited lower diffusion coefficients than homochiral complexes. Thus, we obtain the first coherent and consistent correlation between the experimental chemical shift data (shielding effects) and the observed diffusion coefficient differences based on the analysis of MD data.

**Figure 7:**
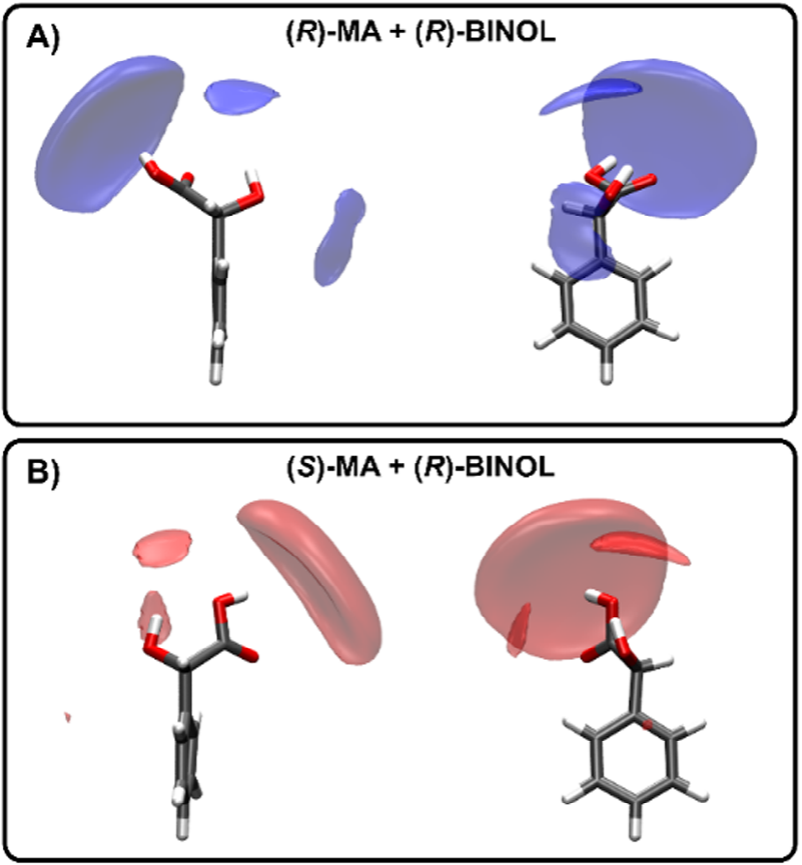
Spatial distribution function (SDF) of (*R*)‐BINOL in the vicinity of A) (*R*)‐MA and B) (*S*)‐MA at an isovalue of 0.5. Front view (right) and side view (left).

The spatial distribution function (SDF) of MA enantiomers in Figure 7 reveals the primary location of (*R*)‐BINOL molecules close to the carboxyl groups of both MA enantiomers. For both diastereomeric complexes, intermolecular interactions are dominated by hydrogen bonds with no significant differences between the enantiomers.

For the (*R*)‐enantiomer of MA, (*R*)‐BINOL molecules can also be found near the hydroxyl group and close to the aromatic ring of MA (Figure 7A). In contrast, for (*S*)‐MA (Figure 7B), BINOL entities are most frequently found in the spatial vicinity of the hydroxyl group, and fewer alternative sites of interactions exist. Thus, both MA enantiomers are capable of forming hydrogen bonds with (*R*)‐BINOL, but (*R*)‐MA can also establish additional non‐ncovalent interactions, such as π─π stacking and van der Waals, which are absent for (*S*)‐MA. Notably, the SDF shows a higher probability of finding (*R*)‐BINOL molecules in the vicinity of the hydrogen at the chiral center of (*R*)‐MA compared to (*S*)‐MA. Consequently, this leads to a larger shielding effect in the NMR spectra around (*R*)‐MA, explaining the lower NMR chemical shifts for this enantiomer. The effect is also seen at other isovalues of 0.30 and 0.4 (see Section S4.7, Supporting Information).

For (*S*)‐BINOL, the SDF is not as discriminative for the MA enantiomers (see page S36, Supporting Information). For both MA enantiomers, BINOL entities are predominantly located close to the hydroxyl and carboxyl groups. Thus, additional computational approaches are required to gain more insights into the molecular chiral recognition mechanisms.

### 2.3 Calculated Gibbs Free Energies and NMR Shifts

**Figure 8:**
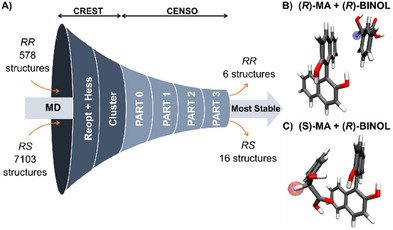
Example workflow for QM refinement of chiral complexes from MD snapshots. A) GFN2‐xTB refinement of complex structures of (*R*,*S*)‐MA and (*R*)‐BINOL from MD trajectories. B) representative homochiral complex and C) heterochiral complex structures. The highlighted atoms indicate the monitored hydrogen atoms at the chiral center.

To complete the molecular‐level picture and obtain quantitative results for chemical shifts and complex stabilities, selected snapshots from the MD simulations were further refined using quantum chemical calculations. The structures were selected based on clearly defined criteria (see Section S2.3, Supporting Information), in which the distance H_MA_···BINOL_COM_ is the most critical parameter for mapping the enantioselective binding. Based on this descriptor, 7103 heterochiral and 578 homochiral structures of complexes with (*R*)‐BINOL were selected from MD snapshots for further refinement at the QM level (see Figure 8). For complexes with (*S*)‐BINOL, 8414 heterochiral and 54 homochiral structures were identified. This higher number of heterochiral complexes is consistent with the more frequent formation of heterochiral complexes than homochiral complexes during the MD simulations.

Prior to accurate DFT calculations, this large number of selected structures from MD simulation had to be reduced by applying a fast but approximate structural re‐optimization at the GFN2‐xTB level. High‐energy and duplicate structures were eliminated, and the remaining structures clustered. The generated conformer–rotamer ensemble (CRE) was further refined at different levels of DFT (see Figure 8 and Section S5, Supporting Information). The number of unique QM‐refined structures at each level is given in Tables S33 and S34, Supporting Information.

After the QM refinement, the final ensemble consisted of 6 homochiral and 16 heterochiral complexes with (*R*)‐BINOL, and 5 homochiral and 11 heterochiral complexes with (*S*)‐BINOL. In both cases, the orientation of the hydrogen atom at the chiral center relative to the BINOL aromatic rings was critical and one of the determinants for the enantiodifferentiation. For instance, in the low energy complex structures with (*R*)‐BINOL, the hydrogen atom in the homochiral complex (Figure 8B) points toward the BINOL aromatic rings, whereas in the heterochiral complex (Figure 8C), it is pointing to the solvent. Thus, the hydrogen atom of (*R*)‐MA is experiencing more electronic density from the BINOL aromatic ring groups, making its chemical environment more shielded in a magnetic field than the hydrogen atom of (*S*)‐MA—exactly as observed in the NMR experiments. In the low energy structures of (*S*)‐MA in complex with (*R*)‐BINOL, stabilization is dominated by hydrogen bonds and π─π stacking or van der Waals interactions are less prominent.

These weaker interactions in the homochiral complexes can explain their shorter lifetimes in MD simulations and larger diffusion coefficients in the NMR measurements. In order to verify this hypothesis, we computed the Gibbs free energies of binding for all diastereomeric complexes. Table 3 gives the calculated Gibbs free energies of binding differences between (*R*)‐ and (*S*)‐MA binding to (*R*,*S*)‐BINOL. In Table 3, examples of the final level of energy calculations (Part 3) are given for different functionals. More data can be found in Section S5.2, Supporting Information. They are all consistent and give more negative Gibbs free energies of binding for the heterochiral complexes. This is in agreement with the experimental results, calculated diffusion coefficient differences, and calculated lifetimes (see above). The calculated differences in Gibbs free energies of binding ΔΔ*G* ^bind^ are small (0.4–2 kcal/mol). Experimental data for chiral recognition in the literature also give such small free energy differences.^[^ ^46^, ^47^, ^48^ ^]^

**Table 3:** Calculated differences in Gibbs free energies of binding ΔΔG*^bind^* of (*R,S*)‐MA with (*R,S*)‐BINOL in kcal/mol (all with a def2‐TZVPD basis set).

Resolving agent	PW6B95^[^ ^a^ ^]^	ωB97X‐V^[^ ^b^ ^]^	PW6B95^[^ ^c^ ^]^	Experimental
(*R*)‐BINOL	+1.484	+0.307	+1.393	>0
(*S*)‐BINOL	−1.849	−1.930	−1.149	<0

^[a]^ Optimized with r^2^scan‐3c/def2‐mTZVPP;

^[b]^ Optimized with ωB97X‐V/def2‐TZVPP;

^[c]^ Optimized with PW6B95‐D3/def2‐TZVPP.

The first column in Table 3 corresponds to the recommended functional and the default settings in CENSO 1.2.0. After an optimization at the r^2^scan‐3c/def2‐mTZVPP level in Part 2, the hybrid meta‐GGA XC functional PW6B95 is used, which is shown to give highly accurate Gibbs free energies of binding for non‐covalent complexes.^[^ ^49^ ^]^ The calculated Gibbs free energy differences are in good agreement with the experiment. In order to investigate the effect of the level of structural optimization, also the range‐separated hybrid, generalized gradient approximation density functional with non‐local correlation ωB97X‐V was used, followed by a Part 3 single‐point calculation with a larger basis set. However, the binding energy differences between homochiral and heterochiral complexes of (*R*,*S*)‐MA with (*R*)‐BINOL were close to 0 kcal/mol, which cannot explain the experimental differences in diffusional properties. An optimization at the hybrid meta‐GGA level also gave a difference of −1.4 kcal/mol for (*R*)‐BINOL, which is consistent with the optimization with the faster composite electronic structure method. However, these highly accurate structures were required to obtain accurate calculated ^1^H NMR chemical shifts (see below). While both PW6B95 and ωB97X‐V produce comparable thermodynamics, ωB97X‐V exhibited inferior performance when calculating the NMR shift differences (see Table 4 and Section S5.3, Supporting Information).

**Table 4:** Calculated differences in ^1^H‐NMR chemical shifts (def2‐TZVPD basis, Δ*δ*_MA_, in ppm) across different DFT functionals for complexation of (*R*,*S*)‐MA with BINOL. Levels of optimizations as in Table 3.

Resolving Agent	PBE0	ωB97X‐V	PW6B95	Experimental
(*R*)‐BINOL	−1.243	−2.163	−0.864	−0.008
(*S*)‐BINOL	+2.294	+2.272	+1.903	+0.008

For calculating ^1^H NMR chemical shifts, nine different DFT levels were critically evaluated (see Section S5.3, Supporting Information). Across the set of functionals and choice of basis set, consistent results were obtained. Differences in the NMR chemical shifts of the discriminating hydrogen atom were calculated in Part 4 and the results of the best performing functionals are presented in the Table 4.

The PW6B95 function provides the best agreement with the experiment, followed by the commonly used PBE0. ωB97X‐V exhibits the largest deviation from the experiment. Although the absolute values of the calculated NMR shifts deviate slightly from experimental data, the computed differences and trends agree well with experimental observations. This agreement in the trends is more crucial for accurately describing the chiral recognition phenomenon in this study than precisely predicting the NMR chemical shifts.

The PW6B95‐D3/def2‐TZVPD results reproduce well the experimental chemical shift differences from NMR, with the homochiral complex having a lower chemical shift compared to the heterochiral complex. It is important to note that solvation effects on NMR shifts and solvation energies were also evaluated using different solvent models SMD, CPCM, and openCOSMO‐RS. All results are consistent, with the SMD model giving a slightly better accuracy in capturing the experimental shift differences.

The calculated shift for the more shielded enantiomer is slightly underestimated, likely due to the overestimation of electronic density effects from the BINOL. However, the ability of DFT methods to calculate very small differences in chemical shifts is pushed to its limit. Here, it can be shown that the combination of MD sampling, with SQM and QM refinement, is able to explain the preferred formation of MA‐BINOL complexes with opposite chirality in terms of thermodynamics and chemical shift differences.

In this joint experimental and computational investigation, it was possible to elucidate the chiral recognition process between BINOL and MA enantiomers in a liquid solution. Dynamics (such as diffusion coefficients and complex lifetimes), thermodynamics (Gibbs free energies of binding), and electronic effects (chemical shifts) must all be considered to be able to discriminate diastereomeric complexes. The reduced diffusion coefficient and a higher chemical shift for (*S*)‐MA in the presence of (*R*)‐BINOL compared to the (*R*)‐stereoisomer was measured and modeled. Although these differences are small, they exceed experimental error, indicating that BINOL preferentially binds to the stereoisomer of opposite chirality. Extensive and multi‐replicate MD simulations accurately reproduced these experimental diffusion coefficient patterns, showing that the heterochiral complex, indeed, exhibited the lower diffusion coefficient. Detailed trajectory analysis revealed that the (*S*)‐enantiomer forms complexes with (*R*)‐BINOL, which have a longer lifetime than the (*R*)‐form, and *vice versa*. The simulations further demonstrated that the MA 1:1 BINOL complexation stoichiometry is dominating and able to explain the observed enantioselective discrimination, with heterochiral complexes forming more frequently than homochiral complexes.

Complex structures that contribute most to experimental separations were identified by applying distinct geometrical descriptors of complexes in the MD simulations. In particular, the distance between the hydrogen atom at the chiral carbon atom of MA and the center of mass of the CRA was relevant. Additionally, the spatial distribution functions of the resolving agent around each stereoisomer indicated that the chiral recognition probably involves multiple and weaker non‐covalent interactions between the (*R*)‐enantiomer and (*R*)‐BINOL, while stronger interactions occur with the (*S*)‐enantiomer. The spatial distribution function also illustrated how the CRA molecules can affect the chemical environment of each stereoisomer and influence their chemical shifts.

The QM refinement of prominent structures from MD snapshots confirmed BINOL's heterochiral binding preference. Overall, the QM results elucidated that the more favorable binding energies for heterochiral complexes resulted in higher complex stability and explained the longer interaction times between BINOL and the MA enantiomer of opposite chirality in MD simulations. The calculated differences in Gibbs free energies of binding were small but consistent and agree with the experiment. QM calculations were also able to rationalize the measured ^1^H NMR chemical shift differences. The orientation of the discriminating hydrogen atom relative to the BINOL rings explained the experiment's NMR chemical shift discrimination.

## 3 Conclusion

In this study, we successfully integrated experimental and computational approaches to rationalize the stereoselectivity of (*R*)‐BINOL and (*S*)‐BINOL recognition for mandelic acid enantiomers. Our precise NMR measurements revealed a clear preference of the CRA for the stereoisomer with opposite chirality and highlighted the ability of NMR to comprehend the chiral recognition in liquid solution experimentally.

For the first time, we demonstrated that extensive molecular dynamics simulations with many replicates can explain the diffusional behavior of these systems while also revealing the principles underlying the chiral recognition observed in diffusion‐NMR experiments at the molecular level. We effectively identified the theoretical key characteristics, such as the lifetimes of diastereomeric complexes, critical complex stoichiometries, and structural criteria essential for understanding the NMR properties of the investigated systems. Our quantum mechanical calculations confirmed the principal characteristics and interactions driving the observed chiral recognition preferences.

Overall, this research establishes a robust approach for future investigations into mechanisms of chiral recognition. It offers a systematic framework for understanding experimental enantiodiscrimination and supporting the design of novel chiral resolving agents. It can also be extended to explore chiral recognition features in mixtures involving enantiomers with different functional groups, sizes, or framework structures. As a final point, the advances presented in this work contribute to the ongoing development of highly accurate spectroscopic methods to determine absolute configurations, which is still a critical challenge for NMR and computational fields.

## 4 Experimental and Computational Section

### 4.1 NMR Methodology

The NMR samples were prepared using 200 mm of (*R* or *S*)‐BINOL (Figure 1) as chiral resolving agents to discriminate an enantiomeric mixture of 28 mm (*R*)‐Mandelic acid with 12 mm (*S*)‐Mandelic Acid (Figure 1). The analysis was carried out in 5 mm NMR tubes with 500 µL of deuterated chloroform (CDCl_3_) as a solvent, doped with 0.03% v/v of the reference compound tetramethyl silane (TMS). All chemicals used in this work were commercially available from Merck and Sigma‐Aldrich and used without further purification. For the samples with different enantiomeric mixture compositions and additional NMR experiments, refer to Supporting Information for further details.

The NMR measurements were conducted with a 14.1 Tesla Bruker Avance III spectrometer at 298.15 K using the TopSpin 3.6.4 software (Bruker Biospin). The equipment was equipped with a TBI probe with a *z*‐gradient coil producing a maximum nominal gradient strength of 53.5 G cm^−1^, operating at 600.17 MHz for ^1^H nuclei. The diffusion experiments were performed using the ^1^H‐Oneshot pulse sequence^[^ ^50^ ^]^ with 16 diffusion increments, where the gradient strength varied quadratically from 10% to 80% of the maximum nominal gradient value. For each increment, 16 scans, 16 dummy scans, and 32k data points were used. The diffusion time (*Δ*) and the gradient pulse duration δ (*p30*) were optimized for each experiment to achieve ≈80% attenuation between the first and last increment.

All acquired diffusion data were processed using GNAT 1.2.3 software^[^ ^51^ ^]^, applying Fourier Transformations, using a Lorentzian window function, and performing manual and individual phase and baseline corrections. The diffusion coefficients and their errors for each species were estimated by fitting the modified Stejskal–Tanner equation, performed by the GNAT. Further information can be found in Supporting Information.

### 4.2 Molecular Dynamics Simulations

A large number of benchmark simulations were performed regarding the choice of force field, composition of the simulation box, simulation length, and number of replicates. For the optimized system of (*R*,*S*)‐MA, (*R*,*S*)‐BINOL with chloroform as the solvent, the general AMBER force field (GAFF)^[^ ^52^ ^]^ with AM1‐BCC^[^ ^53^, ^54^ ^]^ partial atomic charges from the Antechamber program^[^ ^55^ ^]^ were used. Long‐range electrostatic interactions were managed using the reaction‐field method with a cutoff radius of 1.4 nm for the real space term, which was the same distance adopted to perform the neighbor search (Verlet algorithm) and accounting of van der Waals interactions. For all simulations, the starting configuration was built up with the PACKMOL program.^[^ ^56^ ^]^ The overall best‐performing number of 5 molecules of each MA enantiomer with 50 molecules of (*R* or *S*)‐BINOL were randomly included in a cubic simulation box along with 4675 chloroform molecules to mimic the experimental conditions. The initial box size dimension was chosen to correspond to the experimental density of CHCl_3_ at 298.15 K. The MD simulations were conducted using Gromacs 2018^[^ ^57^ ^]^ at 298.15 K.

The first step was a steepest descent energy minimization to converge to a maximum force below 1 kJ mol^−1^ nm^−1^. Next was an isothermal–isobaric ensemble (NPT) simulation, performed for 2 ns (preceded by 500 ps of an equilibration NPT run) to bring the system to the optimal density and adequate simulation box size for the production simulation steps. A Parrinello–Rahman barostat^[^ ^58^ ^]^ with a coupling constant of 2 ps was used to maintain the pressure at 1.0 bar. The final stage was 15 replicates of production runs in an NVT ensemble, each sampling for 100 ns (preceded by 500 ps of equilibration NVT runs). For both NPT and NVT ensembles, the Nosé–Hoover thermostat^[^ ^59^, ^60^ ^]^ with a coupling constant of 0.4 ps was applied to regulate the temperature at 298.15 K. In the MD simulations, the equations of motion were integrated using a time step of 0.1 fs, and the trajectories were saved every 1 ps for further data analysis.

The diffusion coefficients were obtained from the linear regime of the mean square displacement (MSD) curve using the Einstein relation (Equation (S2), Supporting Information). The MSD was computed by Gromacs for each trajectory and averaged over all simulations to obtain the final molecular diffusion coefficient for each compound. The linear regression was applied to the mean MSD over the simulation time curve between 10 and 90 ns. The visual molecular dynamics (VMD) program^[^ ^61^ ^]^ was used to visualize the diastereomeric complexes, calculate their lifetime, determine their stoichiometry, and measure the distances between the hydrogen atoms at the stereogenic centers (as highlighted in Figure 1) and the center of mass of (*R*,*S*)‐BINOL. The TRAVIS program^[^ ^62^ ^]^ was also applied to calculate the 3D spatial distribution functions (SDF) from the concatenated production trajectories. For more details on the MD assessment, detailed data analysis, and frame selection information, see Supporting Information.

### 4.3 Quantum Mechanical Calculations

Selected frames from the MD trajectories were refined using quantum mechanical calculations using the semi‐empirical extended tight‐binding GFN2 Hamiltonian from xTB 6.3.2.^[^ ^63^, ^64^ ^]^ The Conformer–Rotamer Ensemble Sampling Tool (CREST 3.0.2)^[^ ^65^ ^]^ was used for fast and efficient exploration of the conformational chemical space of a large number of complexes to give the final Conformer–Rotamer‐Ensemble. The Commandline Energetic Sorting algorithm (CENSO 1.2.0)^[^ ^66^ ^]^ combined with the Orca 5.0.4 program^[^ ^67^ ^]^ was employed for the refinement calculations of the CRE in terms of electronic energies, thermochemical corrections, and solvation effects.

Initially, the MD configurations were re‐optimized using GFN2‐xTB, and their thermodynamics were computed based on single‐point Hessian (SPH) calculations.^[^ ^68^ ^]^ High‐energy structures were eliminated, and a set of unique structures was obtained by filtering according to root mean square deviations, energies, and rotational constants, as implemented in CREST. The remaining conformers were clustered using a principal component analysis (PCA) and k‐means to reduce a large number of configurations into a set of representative structures, improving the efficiency of subsequent QM calculations.

The clustered ensembles were then refined using a four‐step protocol, and Gibbs free energies and NMR chemical shifts were calculated. In PART 0, the electronic energies were computed from single‐point energy (SPE) calculations at the B97‐D3/def2‐SV(P)+gCP level of theory, with solvation contribution modeled using the ALPB solvation model (at the GFN2‐xTB level). In the second step (PART 1), SPEs were recalculated with r^2^SCAN‐3c/def2‐mTZVPP, including implicit solvation, using the SMD solvation model. Here, SPH calculations were also performed using the modified rigid‐rotor harmonic oscillator (mRRHO) at GFN2‐xTB with an implicit solvent at the ALPB level to incorporate thermostatistical contributions and give Gibbs free energies. In PART 2, two consecutive optimization steps were performed. Initially, the structures were pre‐optimized using r^2^SCAN‐3c/def2‐mTZVPP and then again fully re‐optimized at PW6B95‐D3/def2‐TZVPP level, both with SMD as a solvation model. Thermostatistical contributions were again calculated, as above. This was followed by final single‐point DFT calculations to refine the electronic energies (PART 3) at the hybrid meta‐GGA PW6B95‐D3/def2‐TZVPD level with SMD and mRRHO thermochemical corrections.^[^ ^66^ ^]^ The NMR chemical shifts (*δ*), were finally calculated at the PW6B95‐D3/def2‐TZVPD level of theory and the Gibbs free energies of binding were calculated only for the final ensemble using the same level as in PART 3. All calculated NMR properties and binding energies are given as the Boltzmann‐weighted averages.

Other functionals, basis sets, and solvation models for computing the final SPEs and NMR shifts were also evaluated to assess their performance in the workflow (see Supporting Information). The aforementioned levels of theory were selected because they demonstrated the best performance for predicting the experimental binding and NMR chemical shifts. Further details on the QM calculations and their careful benchmarking are available in Supporting Information.

## Supporting Information

Detailed information on the NMR experiments, molecular dynamics analyses, MD frame selection, and assessment of various QM levels of theory, along with additional data supporting the findings of this study, is provided in the Supplementary Material of this article.^[^ ^69^, ^70^, ^71^, ^72^, ^73^, ^74^ ^]^

## Conflict of Interest

The authors declare no conflict of interest.

## Supporting information



Supporting Information

## Data Availability

All NMR experimental acquisition files; MD settings, topology files, selected MD frames; QM refinement structures and energies at all parts, along with calculated NMR shifts, are freely available in the Edmond repository (Open Research Data Repository of the Max Planck Society) at https://doi.org/10.17617/3.IXJQ6Y.
