# Supplementary material for: Molecular Recognition and Chiral Discrimination from NMR and Multi‐Scale Simulations
Source: Chemistry. 2025 Apr 2;31(25):e202404694. doi: 10.1002/chem.202404694 (PMC12057608; doi:10.1002/chem.202404694)
Supplement: Supplementary file 1 — Supporting Information [file CHEM-31-e202404694-s001.pdf]

Supporting Information

Molecular Recognition and Chiral Discrimination from NMR and Multi-Scale Simulations

Tadeu Luiz Gomes Cabral[a,b], João Pedro Brussolo da Silva[a], Claudio Francisco Tormena[a,*], Matthias Stein[b,*]

[a]Physical Organic Chemistry Lab, Chemistry Institute, University of Campinas - UNICAMP, P.O. Box 6154, 13083-970 Campinas, SP, Brazil.
[b]Molecular Simulations and Design Group, Max Planck Institute for Dynamics of Complex Technical Systems, Sandtorstrasse 1, 39106 Magdeburg, Germany.

Emails:
tormena@unicamp.br
matthias.stein@mpi-magdeburg.mpg.de

Contents

1 Information on NMR Experiments **S2**

1.1 DOSY Data Processing and Statistical Analysis Procedures S2

1.2 DOSY Experiments on Various Enantiomeric Compositions S3

1.3 NOESY Experimental Details S3

2 Information on Molecular Dynamics **S4**

2.1 OPLS-AA Force Field Parameterization and System Setup S4

2.2 Computing Diffusion Coefficients S4

2.3 Analysis of MD Trajectories and Selection of Frames S4

3 Data from NMR Experiments **S6**

3.1 Diffusion NMR Data S6

3.2 Diffusion Data from Different Enantiomeric Mixtures S6

3.3 ^{1}H-^{1}H NOESY Data S11

4 Data from Molecular Dynamics Simulations **S12**

4.1 Impact of Solution Composition and Force Field on Diffusion S12

4.2 Number of replicates and Diffusion Coefficients S23

4.3 Convergence of Diffusion Coefficients with Simulation Lengths S24

4.4 Calculated Diffusion Coefficients for (*R*/*S*)-MA, (*R*/*S*)-BINOL and $CHCl_3$ S28

4.5 Calculated Diastereomeric Complex Lifetime S31

4.6 Calculating the Stoichiometries of Diastereomeric Complexes S33

4.7 Spatial Distribution Functions S35

5 Data from Quantum Chemical Calculations **S36**

5.1 Selection of MD Frames and QM Refinement S36

5.2 Computed Gibbs Free Energies of Binding S37

5.3 DFT calculation of ^{1}H Chemical Shifts S38

5.4 Calculating Solvation Energy S39

6 References **S41**

1 Information on NMR Experiments

1.1 DOSY Data Processing and Statistical Analysis Procedures

The diffusion coefficients and their statistical errors were estimated using an exponential diffusion fitting with peak picking and a mono-exponential fit type, calculated using the GNAT program. Fourier transformations were applied with 128k points using a Lorentzian window function of 0.3 Hz. Manual corrections were performed for individual phase and baseline adjustments (on the order of 5). The spectrum was referenced to the TMS signal at 0 ppm. Statistical analysis was performed to calculate the difference between the diffusion coefficients (ΔD_{RS}) of (*R*)-enantiomers and (*S*)-enantiomers, along with the propagated error (error_{RS}), as shown in the following equations.

$$\Delta D_{RS} \pm \text{error}_{RS} = (D_R - D_S) \pm \sqrt{\text{error}_R^2 + \text{error}_S^2} \tag{1}$$

In equation 1, D_R and D_S represent the diffusion coefficients for the (*R*)-enantiomer and (*S*)-enantiomer, respectively. The terms error_R and error_S correspond to the experimental errors associated with the diffusion coefficients of the (*R*)-stereoisomer and (*S*)-stereoisomer. Reliable diffusion separation is achieved when the difference in diffusion coefficients exceeds the propagated error, e.g., when $\Delta D_{RS} > \text{error}_{RS}$.[1]

The statistical analysis (and the error propagation) for the relative ratio $\frac{\Delta D_{(S)\text{-BINOL}}}{\Delta D_{(R)\text{-BINOL}}}$, representing the absolute difference ratio between (*R*)-BINOL and (*S*)-BINOL, can be calculated using the following equations:[2]

$$\Delta D_{(R)\text{-BINOL}} \pm \text{error}_{(R)\text{-BINOL}} = 0.07 \pm 0.03$$

$$\Delta D_{(S)\text{-BINOL}} \pm \text{error}_{(S)\text{-BINOL}} = -0.11 \pm 0.04$$

The ratio is calculated as:

$$\left| \frac{\Delta D_{(R)\text{-BINOL}}}{\Delta D_{(S)\text{-BINOL}}} \right| = \frac{|0.07|}{|-0.11|} = 1.57$$

To propagate error is computed as:

$$\frac{\text{error}_{\text{prop}}}{\left| \frac{\Delta D_{(R)\text{-BINOL}}}{\Delta D_{(S)\text{-BINOL}}} \right|} = \sqrt{\left(\frac{\text{error}_{(R)\text{-BINOL}}}{\Delta D_{(R)\text{-BINOL}}} \right)^2 + \left(\frac{\text{error}_{(S)\text{-BINOL}}}{\Delta D_{(S)\text{-BINOL}}} \right)^2}$$

$$\frac{\text{error}_{\text{prop}}}{1.57} = \sqrt{\left(\frac{0.03}{0.07} \right)^2 + \left(\frac{0.04}{0.11} \right)^2}$$

$$\frac{\text{error}_{\text{prop}}}{1.57} = 0.56$$

$$\text{error}_{\text{prop}} = 0.56 \times 1.57 = 0.88$$

Thus, the absolute relative ratio and its propagated error are:

$$\left|\frac{\Delta D_{(R)\text{-BINOL}}}{\Delta D_{(S)\text{-BINOL}}}\right| = 1.57 \pm 0.88$$

1.2 DOSY Experiments on Various Enantiomeric Compositions

For this experiment, the samples were prepared using a molar ratio of 1 Mandelic Acid (MA) to 5 BINOL, with a MA concentration of 25 mM and a concentration of 125 mM for both (*R*)-BINOL and (*S*)-BINOL, in 500 µL of deuterated chloroform containing 0.03% v/v of tetramethylsilane (TMS) as the reference compound. Based on these concentrations, different NMR samples were prepared, covering enantiomeric compositions of 70% (*R*)-MA, 50% (*R*)-MA, and 30% (*R*)-MA.

The NMR measurements were conducted on a Bruker Avance III spectrometer at 298.15 K, equipped with a BBI probe with a *z*-gradient coil, producing a maximum nominal gradient strength of 55 G·cm^{-1}, and operating at 400.18 MHz for ^{1}H nuclei. Diffusion experiments were performed using the ^{1}H-Oneshot pulse sequence[3] with 16 diffusion increments, where the gradient strength varied quadratically from 10% to 80% of the maximum nominal gradient value. For each increment, 16 scans, 16 dummy scans, and 32k data points were collected. The diffusion time (Δ) and gradient pulse duration (δ, p30) were optimized for each experiment to achieve approximately 80% attenuation between the first and last increment. The DOSY data processing was conducted as described previously.

1.3 NOESY Experimental Details

The NOESY experiments were conducted on a sample containing 70% (*R*)-MA, (*R*)-BINOL and 5 µL of D_2O (to suppress the OH signal near the MA signals), using a Bruker Avance III spectrometer, equipped with a BBI probe, at a temperature of 298.15 K. The spectrometer operated at a frequency of 400.18 MHz for ^{1}H nuclei.

The NOESY experiments were conducted using the *noesygpphpp* pulse sequence[4,5] (as implemented by Bruker). Experiments were performed with varying mixing times (ranging from 400 to 1000 ms); however, due to the similarity of the results, only the spectrum obtained with a mixing time of 1000 ms is presented. The data acquisition involved 256 increments in the indirect dimension (F1). For each increment, 64 scans and 16 dummy scans were recorded, with 4K data points in the direct dimension (F2). The acquired spectra were processed using automatic baseline and phase corrections, following the parameters recommended in the pulse program: *PHC0* = 90°, *PHC1* = -180°, and *FCOR* = 1.

2 Information on Molecular Dynamics

2.1 OPLS-AA Force Field Parameterization and System Setup

The LigParGen Web server[6] (link: https://traken.chem.yale.edu/ligpargen/) was used with the localized bond-charge corrected CM1A model (1.14*CM1A-LBCC)[7] for the system parametrization. The initial configuration was then constructed using PACKMOL.

2.2 Computing Diffusion Coefficients

The diffusion coefficients (*D*) were calculated using the Einstein relation[8], presented in Equation 2, which describes a random diffusional motion of molecules.

$$D = \frac{1}{2d} \lim_{t \to \infty} \frac{d}{dt} \left\langle [\vec{r}(t) - \vec{r}(0)]^2 \right\rangle \tag{2}$$

Where $\vec{r}(t)$ describes the position of a particle at time t, and $\vec{r}(0)$ denotes the position of the particle at the initial time $t = 0$ (origin). The term $\left\langle [\vec{r}(t) - \vec{r}(0)]^2 \right\rangle$ represents the mean squared displacement (MSD), and d the system's dimension.

In practical terms, the diffusion coefficient is equal to one-sixth of the slope of the linear region of the MSD curve as a function of simulation time. Therefore, Equation 2 can be rewritten as:

$$D = \frac{1}{6} \times \textit{MSD slope} \tag{3}$$

An estimate of the error is provided by calculating the difference between the diffusion coefficients derived from fitting the data over the first and second halves of the fit interval.

2.3 Analysis of MD Trajectories and Selection of Frames

To calculate the complex lifetime, we counted the number of frames in each replicatete, in which any BINOL molecule was in contact distance with any (*R* or *S*)-enantiomer molecules. The total number of such frames was then converted into nanoseconds by correlating the number of frames with BINOL-enantiomer interactions to the total frames over a simulation period of 100 ns. The average across all replicatetes was calculated, and the associated error was determined as the standard deviation divided by the square root of the number of replicates.

A distance cutoff of 5.5 Å between any atoms of BINOL and any atoms of (*R* or *S*)-enantiomers was applied. This cutoff was selected based on the typical and expected interactions relevant to chiral recognition, such as π-stacking, hydrogen bonding, van der Waals interactions, and others.[9] This range is considered appropriate for capturing all relevant intermolecular interactions.

In order to calculate the number of complexes with stoichiometries of (BINOL:MA) 1:1, 1:2, 1:3, 1:4, and 1:5 for each stereoisomer, we counted the number of BINOL molecules interacting with each (*R* or *S*)-enantiomer for each MD replicatete, adhering to the same distance criteria of 5.5 Å. The final frequency of each complex stoichiometry was then calculated as the mean value across all

replicatetes, with the error determined as the standard deviation divided by the square root of the number of replicates.

For the analysis of the distance between the hydrogen atoms at the chiral center and the center of mass (COM) of BINOL, the 1:1 diastereoisomeric complexes distances were calculated using the VMD program.

Regarding the frame selection, the distance between the hydrogen atom at the chiral center and the COM of BINOL was chosen as a criterion. Initially, we chose the distance intervals where differences in the occurrence of complexes between (*R* or *S*)-BINOL with (*R*)-MA and (*R* or *S*)-BINOL with (*S*)-MA were observed exceeding the margin of error (**Figure S1A**). After identifying the distance intervals with significant differences, the magnitude of these differences was quantified, as depicted in **Figure S1B**. Next, the distance intervals were divided into 2.5 Å increments, with the interval showing the highest frequency difference selected, as illustrated by the orange bars in **Figure S1C**.

To conclude the frame selection process, structures were selected based on diastereomeric complexes that have higher occurrence within the chosen distance interval. For example, if complexes between BINOL and (*R*)-MA are more frequent within a given interval compared to complexes with (*S*)-MA, it is assumed that the BINOL-(*R*)-MA complex is more representative for that distance interval. Thus, these structures were subsequently chosen for further refinement using QM calculations. Throughout these procedures, the frame selection ensured that homochiral and heterochiral structures with similar structures were not included, as they were expected to produce comparable effects and would not significantly contribute to rationalizing the experimental differences.

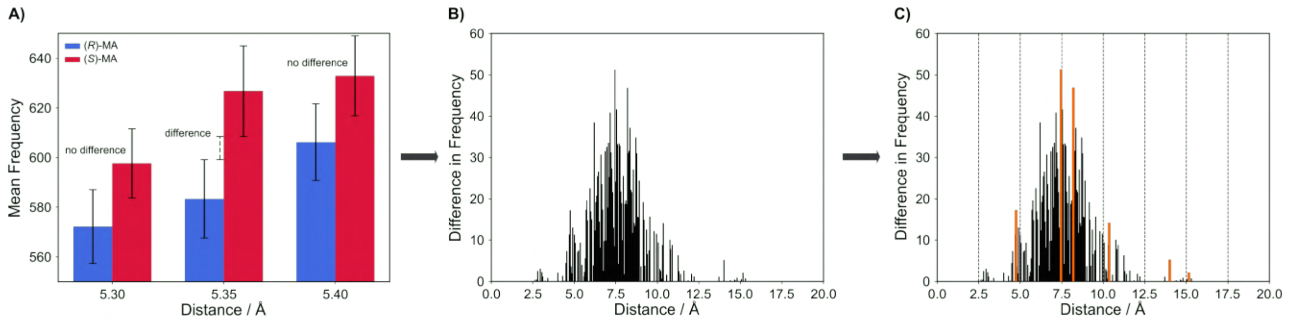

Figure S1: A) Distance intervals without and with significant differences in the mean frequency of complex formation between (*R*)-BINOL with (*R* or *S*)-MA. **B)** Magnitude of the difference in mean frequencies for distance intervals where a significant difference exists. **C)** Distance intervals divided into 2.5 Å increments. The orange bars represent the maximum frequency difference within each increment.

For further analysis using TRAVIS, the entire trajectories were concatenated. For the spatial distribution functions (SDF) analysis, complexes with a 1:1 stoichiometry (MA:BINOL) were saved in *gro* file format, and the subsequent analyses were conducted on all frames. The enantiomers were set as the reference molecules, while BINOL was designated as the observed molecule. The selected atoms in the reference molecule were the chiral carbon, the hydrogen and the oxygen bound to this carbon. In the observed molecule, all atoms were considered. The VMD program was used to visualize the SDFs.

3 Data from NMR Experiments

3.1 Diffusion NMR Data

Table S1: Experimental measured ^{1}H-DOSY (600 MHz) for Mandelic Acid (MA) enantiomers in the absence and presence of (*R*)-BINOL and (*S*)-BINOL. Frequency is given in ppm, and diffusion coefficients in x10^{-10} m^2 s^{-1}.

System	Enantiomer	Frequency	Exp. Ampl.	Fit. Ampl.	Error	Diff. coef.	Error
MA (free)	(*R*/*S*)-MA	5.25399	0.54932	0.56357	0.00147	13.43984	0.06539
MA + (*R*)-BINOL	(*R*)-MA	5.15683	0.04509	0.04623	0.00004	11.14857	0.01866
	(*S*)-MA	5.16545	0.03572	0.03665	0.00003	11.07783	0.01853
MA + (*S*)-BINOL	(*S*)-MA	5.17847	0.01512	0.01557	0.00002	12.84427	0.03251
	(*R*)-MA	5.18610	0.02232	0.02304	0.00004	12.73106	0.03393

Table S2: ^{1}H-DOSY (500 MHz) for Mandelic Acid (MA) enantiomers in the absence and presence of (*R*)-BINOL and (*S*)-BINOL from the Salome and Tormena[10]. Frequency is given in ppm, and diffusion coefficients in x10^{-10} m^2 s^{-1}.

System	Frequency	Exp. Ampl.	Fit. Ampl.	Error	Diff. coef.	Error
MA + (*R*)-BINOL	5.18197	3.46637	3.46083	0.00459	11.08037	0.02732
	5.19007	3.38298	3.38872	0.00387	10.78122	0.02320
MA + (*S*)-BINOL	5.22792	2.95133	2.96586	0.00437	10.41542	0.02602
	5.23574	2.86011	2.87325	0.00375	10.73493	0.02281

3.2 Diffusion Data from Different Enantiomeric Mixtures

Table S3: Experimental difference between the diffusion coefficients (ΔD_{RS}, in x10^{-10} m^2 s^{-1}) and the frequencies ($\Delta\delta_{RS} = \delta_R - \delta_S$, in Hz) of the (*R*)-MA and (*S*)-MA in the presence of (*R*)-BINOL and (*S*)-BINOL across different enantiomeric mixture compositions.

Mixture Composition	CRA	$\Delta D_{RS} \pm$ error$_{RS}$	$\Delta\delta_{RS}$
70% of (*R*)-MA	(*R*)-BINOL	0.16 ± 0.07	-2.69
	(*S*)-BINOL	-0.23 ± 0.06	5.01
50% of (*R*)-MA	(*R*)-BINOL	0.08 ± 0.02	-4.07
	(*S*)-BINOL	-0.12 ± 0.03	3.95
30% of (*R*)-MA	(*R*)-BINOL	0.15 ± 0.10	-4.89
	(*S*)-BINOL	-0.21 ± 0.07	3.03

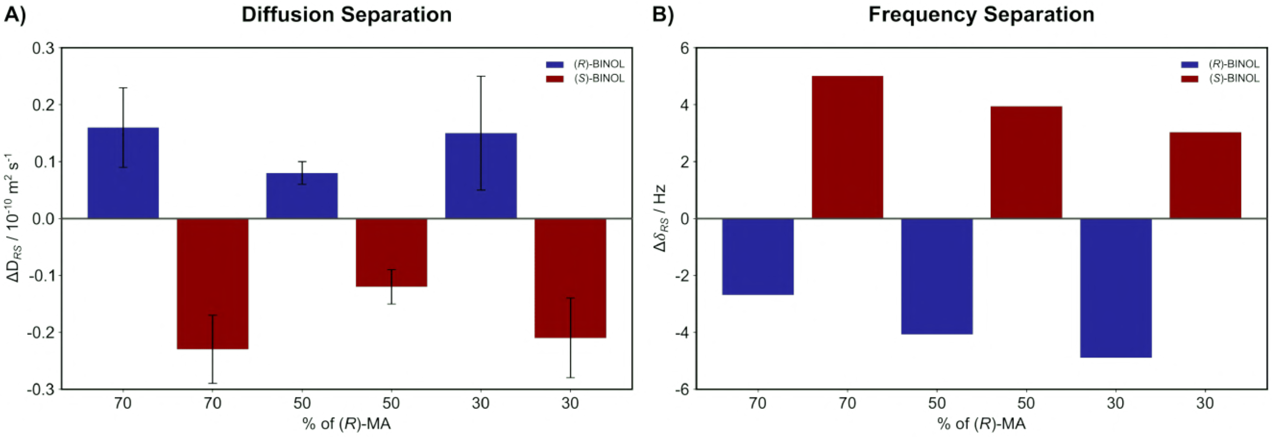

Figure S2: Experimental separations in the **A)** diffusion and **B)** frequency dimensions between (*R*)-MA and (*S*)-MA enantiomers in the presence of (*R*/*S*)-BINOL across varying enantiomeric mixture compositions.

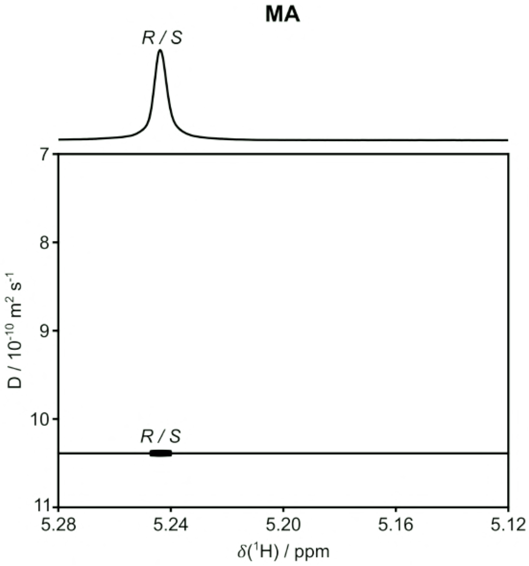

Figure S3: 400 MHz ^{1}H-DOSY with the least attenuated 1D spectrum displayed at the top for an enantiomeric mixture of Mandelic Acid (MA). The mixture contains 70% (*R*)-MA and 30% (*S*)-MA, with deuterated chloroform as the solvent.

Table S4: Experimental measured ^{1}H-DOSY (400 MHz) for Mandelic Acid (MA) enantiomers. The mixture contains 70% (*R*)-MA and 30% (*S*)-MA. Frequency is given in ppm, and diffusion coefficients in x10^{-10} m^{2} s^{-1}.

System	Enantiomer	Frequency	Exp. Ampl.	Fit. Ampl.	Error	Diff. coef.	Error
MA	(*R/S*)-MA	5.24390	0.99981	1.02252	0.00074	10.38713	0.01360

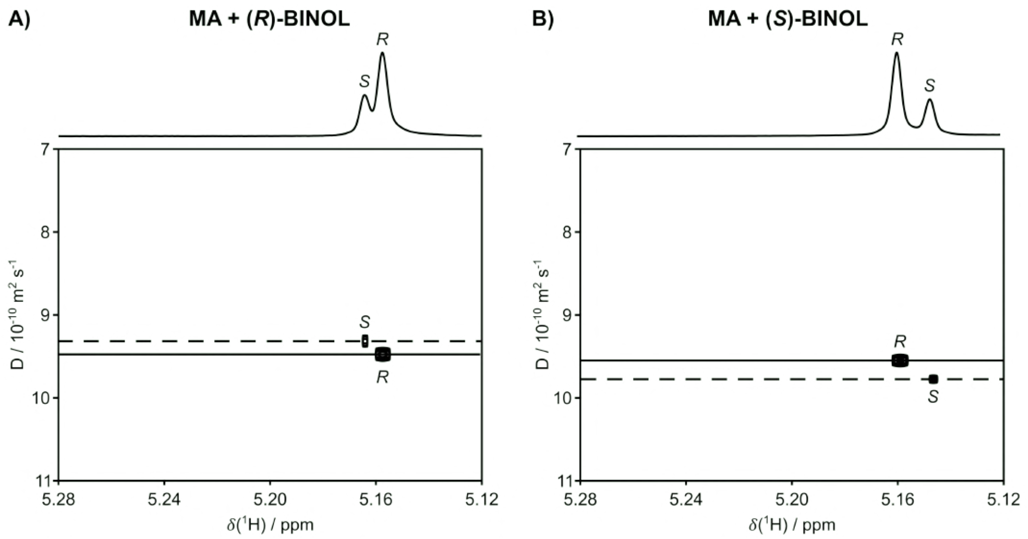

Figure S4: 400 MHz ^{1}H-DOSY with the least attenuated 1D spectrum displayed at the top for an enantiomeric mixture of Mandelic Acid (MA) with **A)** (*R*)-BINOL and **B)** with (*S*)-BINOL. The mixture contains 70% (*R*)-MA and 30% (*S*)-MA, with deuterated chloroform as the solvent.

Table S5: Experimental measured ^{1}H-DOSY (400 MHz) for Mandelic Acid (MA) enantiomers in the presence of (*R*)-BINOL and (*S*)-BINOL. The mixture contains 70% (*R*)-MA and 30% (*S*)-MA. Frequency is given in ppm, and diffusion coefficients in x10^{-10} m^2 s^{-1}.

System	Enantiomer	Frequency	Exp. Ampl.	Fit. Ampl.	Error	Diff. coef.	Error
MA + (*R*)-BINOL	(*R*)-MA	5.15761	0.06360	0.06469	0.00018	9.47522	0.04483
	(*S*)-MA	5.16433	0.03141	0.03191	0.00011	9.32005	0.05410
MA + (*S*)-BINOL	(*S*)-MA	5.14667	0.05456	0.05658	0.00013	9.77521	0.03678
	(*R*)-MA	5.15918	0.12171	0.12608	0.00032	9.54962	0.04099

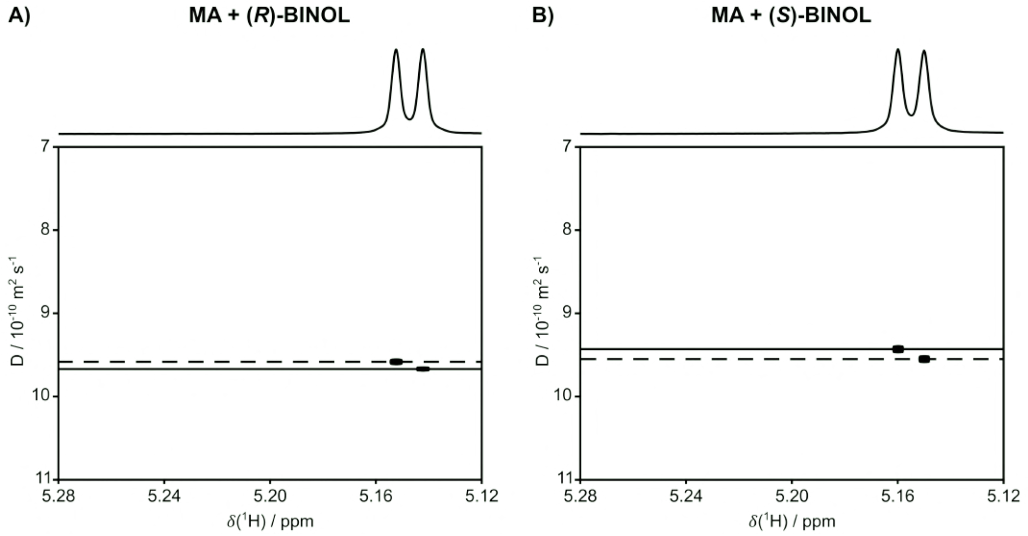

Figure S5: 400 MHz ^{1}H-DOSY with the least attenuated 1D spectrum displayed at the top for an enantiomeric mixture of Mandelic Acid (MA) with **A)** (*R*)-BINOL and **B)** with (*S*)-BINOL. The mixture contains 50% (*R*)-MA and 50% (*S*)-MA, with deuterated chloroform as the solvent.

Table S6: Experimental measured ^{1}H-DOSY (400 MHz) for Mandelic Acid (MA) enantiomers in the presence of (*R*)-BINOL and (*S*)-BINOL. The mixture contains 50% (*R*)-MA and 50% (*S*)-MA. Frequency is given in ppm, and diffusion coefficients in x10^{-10} m^2 s^{-1}.

System	Enantiomer	Frequency	Exp. Ampl.	Fit. Ampl.	Error	Diff. coef.	Error
MA + (*R*)-BINOL	(*R*)-MA	5.14223	0.08382	0.08615	0.00006	9.66626	0.01106
	(*S*)-MA	5.15239	0.08330	0.08555	0.00009	9.58391	0.01790
MA + (*S*)-BINOL	(*S*)-MA	5.15003	0.07136	0.07365	0.00010	9.55049	0.02229
	(*R*)-MA	5.15989	0.07265	0.07470	0.00011	9.43069	0.02424

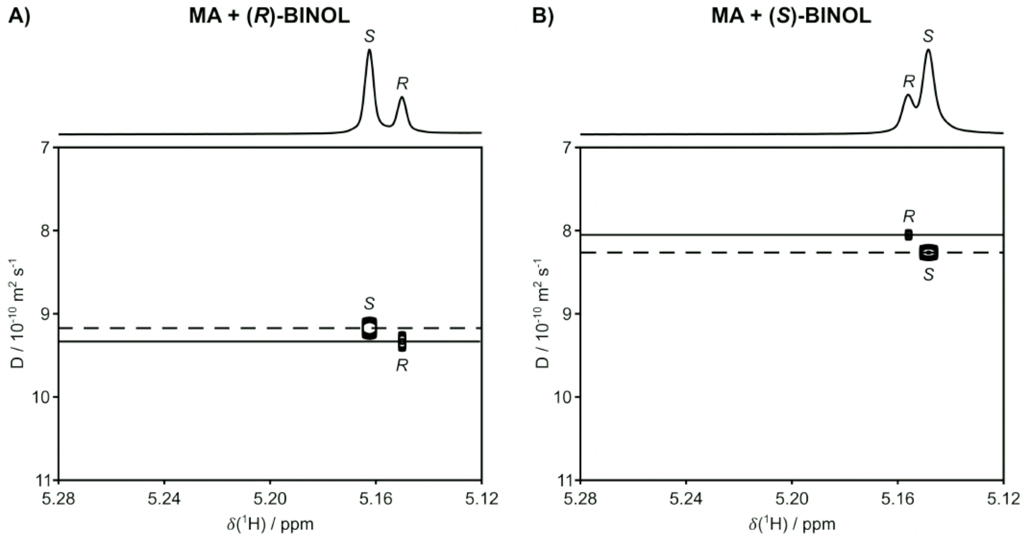

Figure S6: 400 MHz ^{1}H-DOSY with the least attenuated 1D spectrum displayed at the top for an enantiomeric mixture of Mandelic Acid (MA) with **A)** (*R*)-BINOL and **B)** with (*S*)-BINOL. The mixture contains 30% (*R*)-MA and 70% (*S*)-MA, with deuterated chloroform as the solvent.

Table S7: Experimental measured ^{1}H-DOSY (400 MHz) for Mandelic Acid (MA) enantiomers in the presence of (*R*)-BINOL and (*S*)-BINOL. The mixture contains 30% (*R*)-MA and 70% (*S*)-MA. Frequency is given in ppm, and diffusion coefficients in x10^{-10} m^2 s^{-1}.

System	Enantiomer	Frequency	Exp. Ampl.	Fit. Ampl.	Error	Diff. coef.	Error
MA + (*R*)-BINOL	(*R*)-MA	5.15024	0.05014	0.05228	0.00025	9.32906	0.07597
	(*S*)-MA	5.16247	0.11239	0.11670	0.00047	9.17703	0.06379
MA + (*S*)-BINOL	(*S*)-MA	5.14845	0.09538	0.09768	0.00035	8.26325	0.05434
	(*R*)-MA	5.15603	0.04497	0.04612	0.00014	8.05072	0.04675

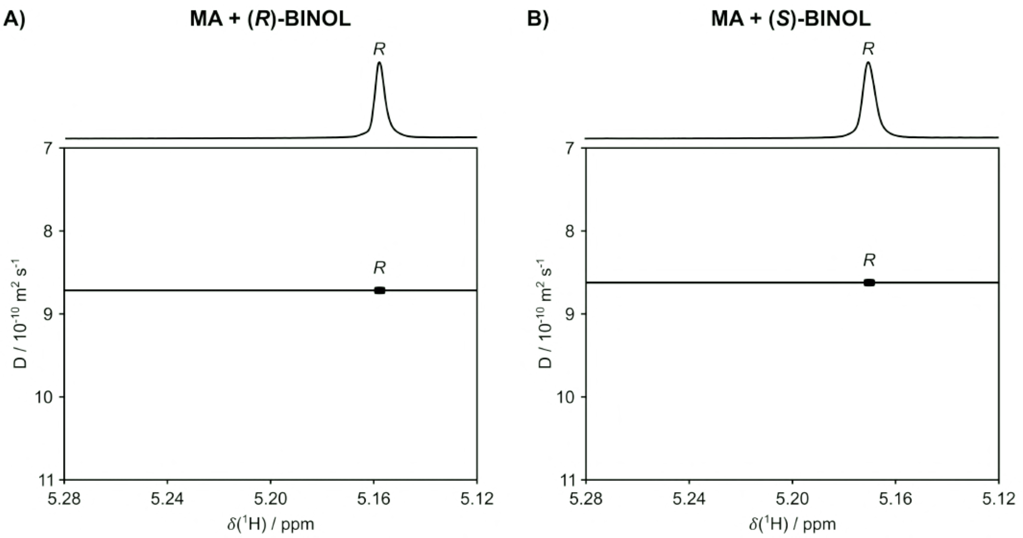

Figure S7: 400 MHz ^{1}H-DOSY with the least attenuated 1D spectrum displayed at the top for an enantiomeric mixture of Mandelic Acid (MA) with **A)** (*R*)-BINOL and **B)** with (*S*)-BINOL. The mixture contains 100% (*R*)-MA and 0% (*S*)-MA, with deuterated chloroform as the solvent.

Table S8: Experimental measured ^{1}H-DOSY (400 MHz) for Mandelic Acid (MA) enantiomers in the presence of (*R*)-BINOL and (*S*)-BINOL. The mixture contains 100% (*R*)-MA and 0% (*S*)-MA. Frequency is given in ppm, and diffusion coefficients in x10^{-10} m^2 s^{-1}.

System	Enantiomer	Frequency	Exp. Ampl.	Fit. Ampl.	Error	Diff. coef.	Error
MA + (*R*)-BINOL	(*R*)-MA	5.15796	0.09303	0.09572	0.00014	8.71662	0.02331
MA + (*S*)-BINOL	(*R*)-MA	5.17040	0.12025	0.12298	0.00022	8.62257	0.02811

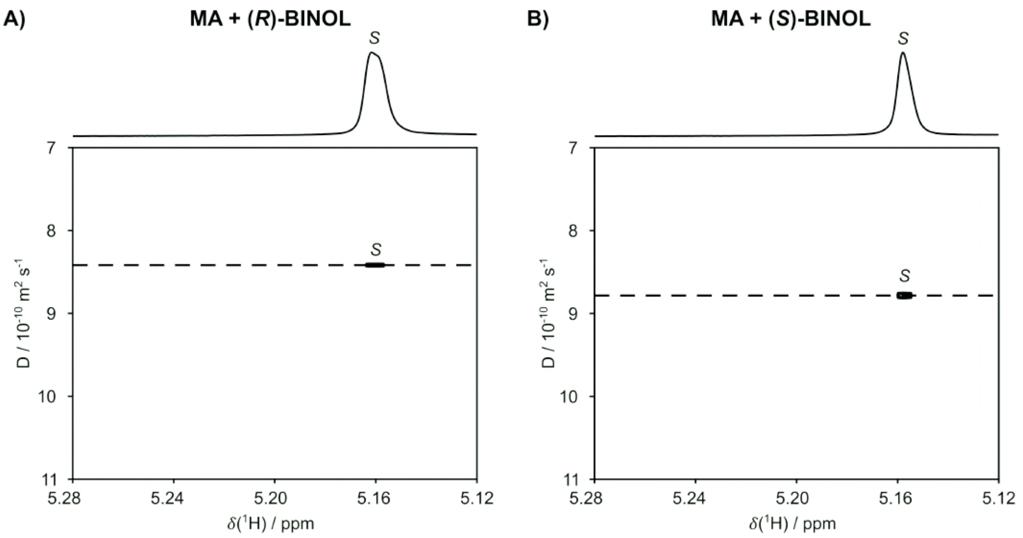

Figure S8: 400 MHz ^{1}H-DOSY with the least attenuated 1D spectrum displayed at the top for an enantiomeric mixture of Mandelic Acid (MA) with **A)** (*R*)-BINOL and **B)** with (*S*)-BINOL. The mixture contains 0% (*R*)-MA and 100% (*S*)-MA, with deuterated chloroform as the solvent.

Table S9: Experimental measured ^{1}H-DOSY (400 MHz) for Mandelic Acid (MA) enantiomers in the presence of (*R*)-BINOL and (*S*)-BINOL. The mixture contains 0% (*R*)-MA and 100% (*S*)-MA.Frequency is given in ppm, and diffusion coefficients in x10^{-10} m^2 s^{-1}.

System	Enantiomer	Frequency	Exp. Ampl.	Fit. Ampl.	Error	Diff. coef.	Error
MA + (*R*)-BINOL	(*S*)-MA	5.16168	0.08815	0.09049	0.00008	8.41068	0.01362
MA + (*S*)-BINOL	(*S*)-MA	5.15789	0.08577	0.08780	0.00013	8.78924	0.02323

3.3 ^{1}H-^{1}H NOESY Data

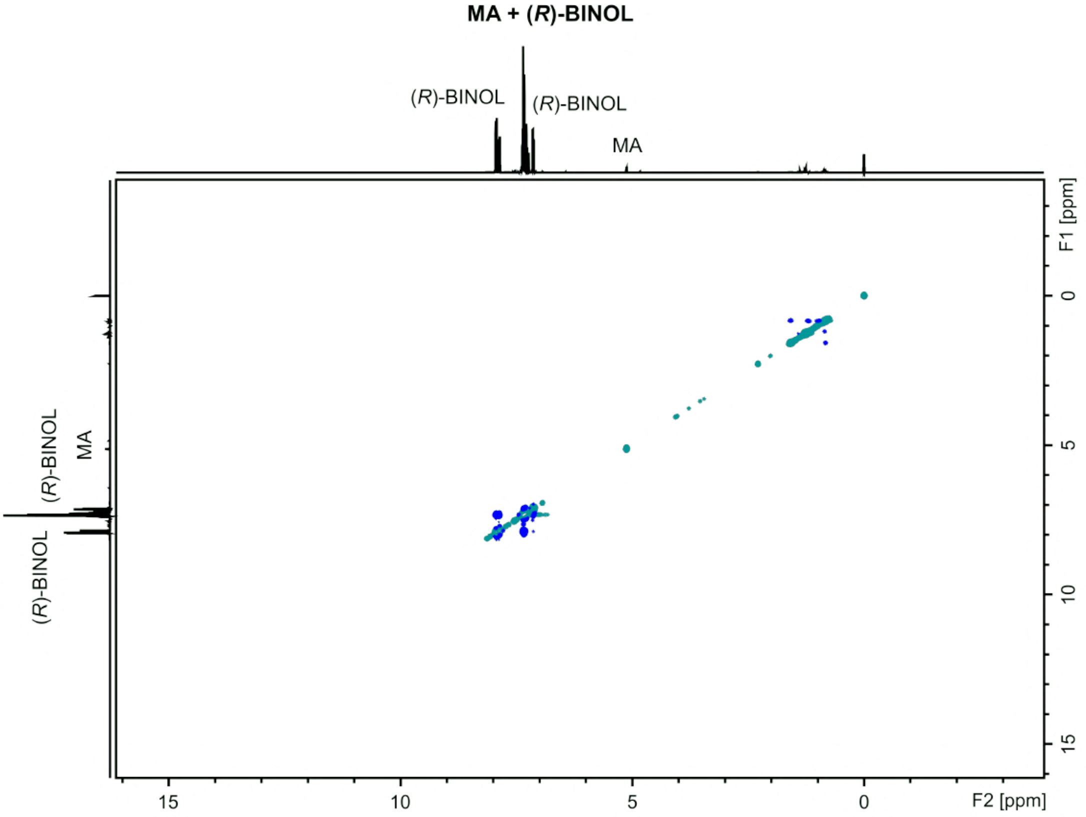

Figure S9: 400 MHz ^{1}H-^{1}H NOESY contour map for an enantiomeric mixture of Mandelic Acid (MA) (70% (*R*)-MA and 30% (*S*)-MA) in the presence of (*R*)-BINOL and 5 µL of D_2O, using deuterated chloroform as the solvent.

It is important to note that in the NOESY contour map presented in Figure S9, no correlation was observed between the hydrogen atom bonded to the asymmetric carbon of MA and the aromatic hydrogens of BINOL. However, this lack of observed correlation does not necessarily imply the absence of intermolecular interactions between the two compounds. Due to the inefficient or negligible spin diffusion effect in small molecules, intermolecular homonuclear NOE is less pronounced than intramolecular NOE.[11] Consequently, the cross-relaxation process during the NOE experiment was not effective in detecting the correlation between the signals of MA and CRA, even using different mixing times from 400 ms to 1000 ms.

4 Data from Molecular Dynamics Simulations

4.1 Impact of Solution Composition and Force Field on Diffusion

Table S10: Number of molecules of MA enantiomers, (*R*)-BINOL, and chloroform in the simulation box, along with the force field used for each studied condition.

Conditions	Number of Molecules				
	**(*R*)-MA**	**(*S*)-MA**	**(*R*)-BINOL**	**$CHCl_3$**	**Force Field**
I	1	1	10	935	GAFF
II	5	5	50	2338	
III	5	5	50	4675	
IV[a]	1	1	10	468	
V[a]	5	5	25	1169	
I	1	1	10	935	OPLS
II	5	5	50	2338	
III	5	5	50	4675	
IV[a]	1	1	10	468	
V[a]	5	5	25	1169	

[a] Under these conditions, the simulations were conducted in two separate boxes, each containing one enantiomer in the presence of (*R*)-BINOL and chloroform molecules.

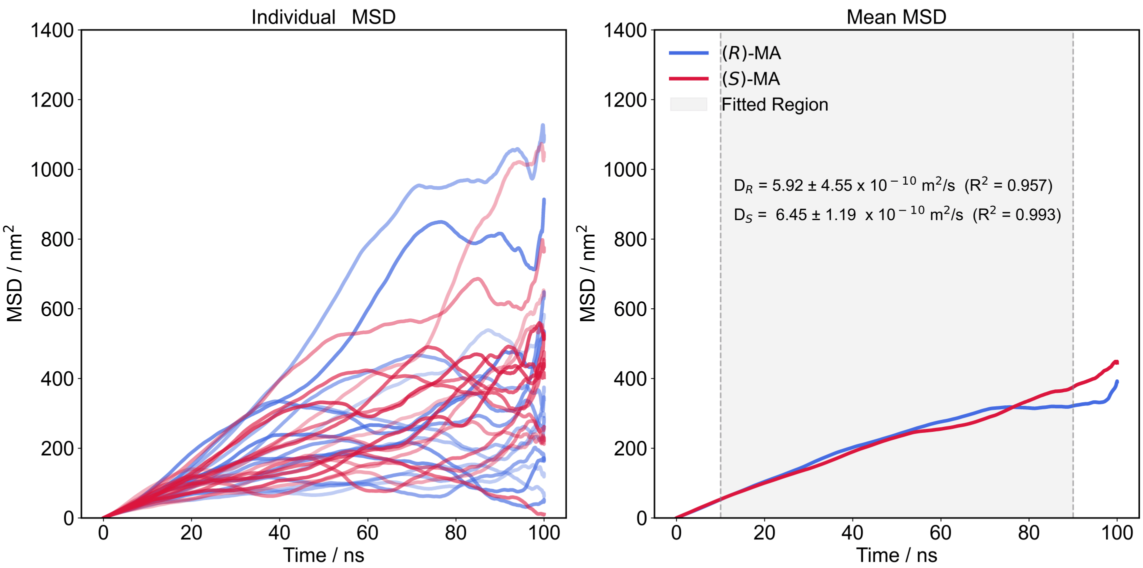

Figure S10: Mean square displacement (MSD) over time for (*R*)-MA (blue) and (*S*)-MA (red) obtained from 15 independent MD simulations of a cubic containing 1 molecule of (*R*)-MA, 1 molecule of (*S*)-MA, 10 molecules of (*R*)-BINOL and 935 molecules of $CHCl_3$ (left panel). The right panel shows the mean MSD obtained from an average of all MSD curves plotted against the simulation time. The gray area indicates the region used to fit to obtain the diffusion coefficients. All molecules were parameterized with GAFF force field.

Table S11: Calculated diffusion coefficients (**D**, in x10^{-10} m^2 s^{-1}), estimated errors (**error**), and correlation coefficients (**R^2**) for each MA enantiomer obtained from different MD replicates of a simulation containing 1 molecule of (*R*)-MA, 1 molecule of (*S*)-MA, 10 molecules of (*R*)-BINOL and 935 molecules of $CHCl_3$. All molecules were parameterized with GAFF force field.

Replicate	(*R*)-MA			(*S*)-MA		
	**D**	**error**	**R^2**	**D**	**error**	**R^2**
01	10.48	1.45	0.994	8.03	0.99	0.993
02	0.82	0.33	0.629	15.59	17.26	0.894
03	2.22	1.08	0.945	1.88	2.25	0.788
04	3.28	3.60	0.804	5.74	6.18	0.773
05	2.29	0.51	0.923	3.86	6.60	0.766
06	2.56	18.16	0.232	12.59	11.74	0.942
07	23.09	5.37	0.973	2.10	0.77	0.730
08	9.45	13.42	0.849	7.41	5.45	0.939
09	0.49	2.44	0.110	1.36	1.40	0.241
10	4.58	2.54	0.946	0.92	10.84	0.095
11	3.88	9.32	0.492	6.90	17.65	0.702
12	1.51	9.59	0.191	9.00	1.23	0.897
13	0.14	9.66	0.003	7.24	10.52	0.841
14	17.89	3.49	0.958	6.64	5.71	0.929
15	6.14	1.06	0.896	7.45	4.91	0.901
**Average**	5.92	4.55	0.957	6.45	1.19	0.993

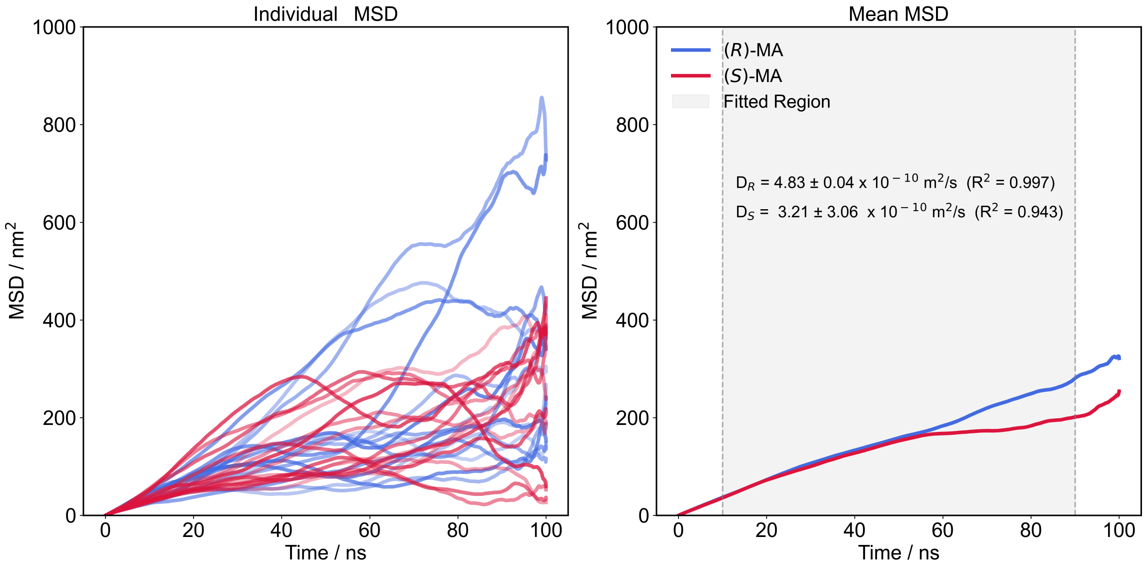

Figure S11: Mean square displacement (MSD) over time for (*R*)-MA (blue) and (*S*)-MA (red) obtained from 15 independent MD simulations of a cubic containing 1 molecule of (*R*)-MA, 1 molecule of (*S*)-MA, 10 molecules of (*R*)-BINOL and 935 molecules of $CHCl_3$ (left panel). The right panel shows the mean MSD obtained from an average of all MSD curves plotted against the simulation time. The gray area indicates the region used for fitting to obtain the diffusion coefficients. All molecules were parameterized with OPLS-AA.

Table S12: Calculated diffusion coefficients (**D**, in x10^{-10} m^2 s^{-1}), estimated errors (**error**), and correlation coefficients (**R^2**) for each MA enantiomer obtained from different MD replicates of a simulation containing 1 molecule of (*R*)-MA, 1 molecule of (*S*)-MA, 10 molecules of (*R*)-BINOL and 935 molecules of $CHCl_3$. All molecules were parameterized with OPLS-AA force field.

**Replicate**	**(*R*)-MA**			**(*S*)-MA**		
	**D**	**error**	**R^2**	**D**	**error**	**R^2**
01	1.91	6.86	0.507	7.10	4.04	0.964
02	2.75	2.22	0.856	3.10	4.89	0.817
03	2.86	5.75	0.785	2.93	1.48	0.955
04	10.51	11.29	0.895	1.40	3.01	0.722
05	4.75	2.65	0.931	4.88	1.47	0.987
06	0.78	0.34	0.828	3.89	5.82	0.859
07	14.42	2.71	0.981	-0.12	5.51	0.006
08	3.33	2.04	0.948	4.19	7.48	0.789
09	1.92	3.28	0.600	4.58	6.77	0.848
10	2.83	0.33	0.941	-0.99	9.02	0.137
11	8.73	9.29	0.922	4.46	9.86	0.752
12	11.14	21.40	0.782	2.47	0.90	0.914
13	0.25	1.68	0.029	3.07	2.72	0.930
14	2.38	4.43	0.698	2.58	7.27	0.354
15	3.88	0.32	0.890	4.57	7.40	0.671
**Average**	4.83	0.04	0.997	3.21	3.06	0.943

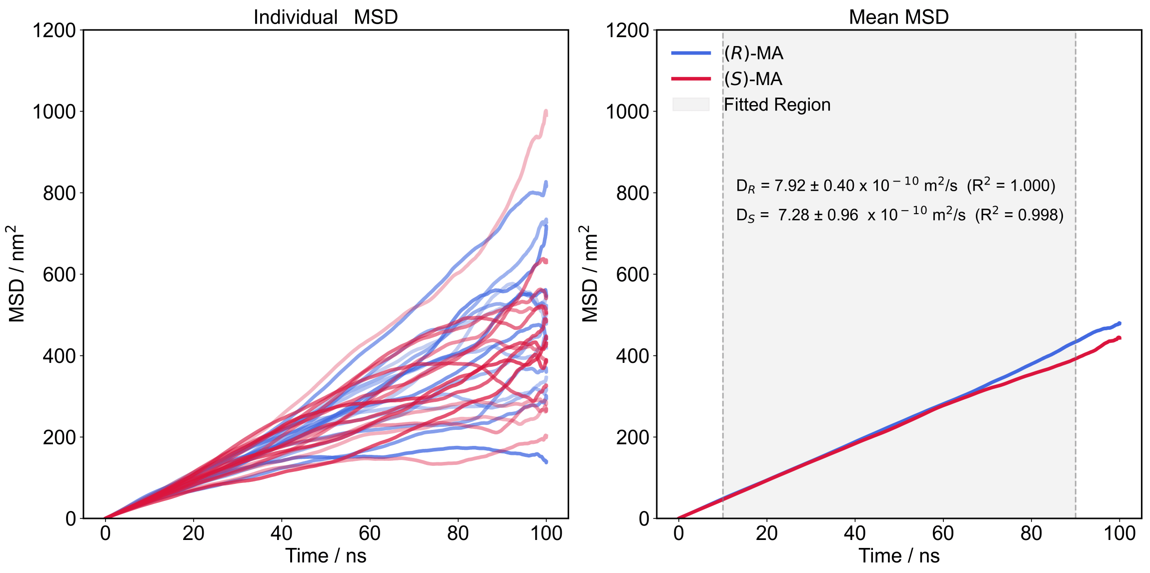

Figure S12: Mean square displacement (MSD) over time for (*R*)-MA (blue) and (*S*)-MA (red) obtained from 15 independent MD simulations of a cubic containing 5 molecules of (*R*)-MA, 5 molecules of (*S*)-MA, 50 molecules of (*R*)-BINOL and 2338 molecules of $CHCl_3$ (left panel). The right panel shows the mean MSD obtained from an average of all MSD curves plotted against the simulation time. The gray area indicates the region used for fitting to obtain the diffusion coefficients. All molecules were parameterized with GAFF force field.

Table S13: Calculated diffusion coefficients (**D**, in x10^{-10} m^2 s^{-1}), estimated errors (**error**), and correlation coefficients (**R^2**) for each MA enantiomer obtained from different MD replicates of a simulation containing 5 molecules of (*R*)-MA, 5 molecules of (*S*)-MA, 50 molecules of (*R*)-BINOL and 2338 molecules of $CHCl_3$. All molecules were parameterized with GAFF force field.

**Replicate**	**(*R*)-MA**			**(*S*)-MA**		
	**D**	**error**	**R^2**	**D**	**error**	**R^2**
01	10.16	0.12	0.999	13.90	2.75	0.994
02	9.53	6.16	0.957	4.37	1.80	0.984
03	4.58	7.20	0.863	5.44	2.22	0.982
04	8.14	2.86	0.990	2.04	3.79	0.795
05	5.48	4.67	0.938	3.65	5.80	0.847
06	6.87	2.38	0.990	9.52	4.19	0.985
07	11.02	2.70	0.990	8.88	2.75	0.991
08	11.06	1.98	0.991	10.82	0.41	0.990
09	4.22	3.73	0.926	7.98	0.47	0.992
10	14.32	5.65	0.989	10.53	1.50	0.989
11	4.26	0.06	0.998	7.27	8.49	0.905
12	7.15	0.74	0.989	5.98	2.08	0.989
13	10.48	8.34	0.956	6.92	3.52	0.978
14	9.23	1.55	0.994	5.57	3.83	0.970
15	2.33	3.66	0.821	6.30	4.90	0.949
**Average**	7.92	0.40	1.000	7.28	0.96	0.998

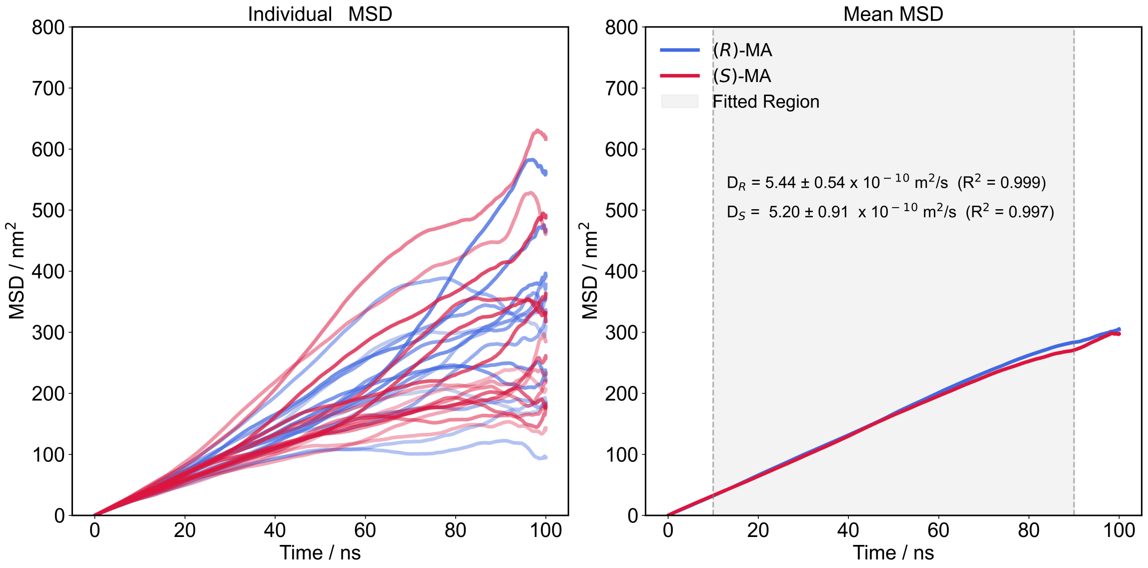

Figure S13: Mean square displacement (MSD) over time for (*R*)-MA (blue) and (*S*)-MA (red) obtained from 15 independent MD simulations of a cubic containing 5 molecules of (*R*)-MA, 5 molecules of (*S*)-MA, 50 molecules of (*R*)-BINOL and 2338 molecules of $CHCl_3$ (left panel). The right panel shows the mean MSD obtained from an average of all MSD curves plotted against the simulation time. The gray area indicates the region used for fitting to obtain the diffusion coefficients. All molecules were parameterized with OPLS-AA force field.

Table S14: Calculated diffusion coefficients (**D**, in x10^{-10} m^2 s^{-1}), estimated errors (**error**), and correlation coefficients (**R^2**) for each MA enantiomer obtained from different MD replicates of a simulation containing 5 molecules of (*R*)-MA, 5 molecules of (*S*)-MA, 50 molecules of (*R*)-BINOL and 2338 molecules of $CHCl_3$. All molecules were parameterized with OPLS-AA force field.

Replicate	(*R*)-MA			(*S*)-MA		
	D	error	R^2	D	error	R^2
01	3.85	3.65	0.920	4.47	0.06	0.997
02	6.64	2.78	0.966	2.42	2.27	0.936
03	2.38	1.25	0.961	3.33	1.19	0.989
04	1.56	2.98	0.783	3.89	2.37	0.970
05	5.85	3.34	0.978	9.18	3.23	0.991
06	2.97	2.96	0.934	3.67	0.68	0.991
07	8.32	5.66	0.939	2.83	4.31	0.859
08	4.88	2.53	0.963	11.27	2.07	0.984
09	6.97	1.12	0.994	3.46	2.50	0.954
10	6.17	1.14	0.987	3.51	2.36	0.970
11	4.76	1.73	0.976	3.50	1.07	0.993
12	4.80	3.55	0.962	7.21	4.19	0.974
13	6.10	1.97	0.992	4.55	2.78	0.975
14	7.30	0.58	0.996	6.29	5.42	0.952
15	9.07	9.25	0.926	8.48	0.76	0.995
**Average**	5.44	0.54	0.999	5.20	0.91	0.997

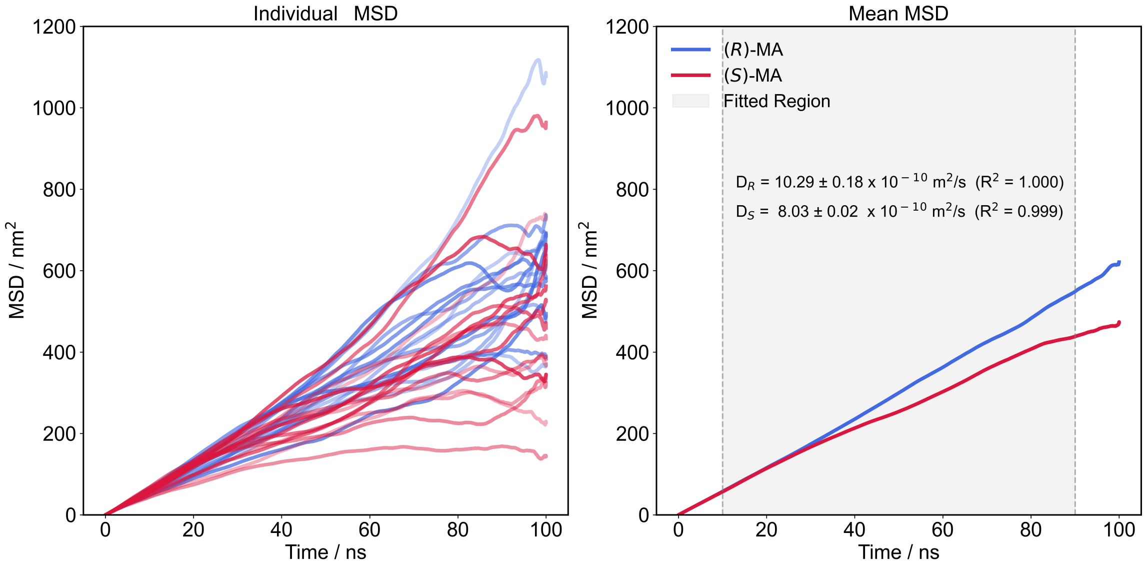

Figure S14: Mean square displacement (MSD) over time for (*R*)-MA (blue) and (*S*)-MA (red) obtained from 15 independent MD simulations of a cubic containing 5 molecules of (*R*)-MA, 5 molecules of (*S*)-MA, 50 molecules of (*R*)-BINOL and 4675 molecules of $CHCl_3$ (left panel). The right panel shows the mean MSD obtained from an average of all MSD curves plotted against the simulation time. The gray area indicates the region used for fitting to obtain the diffusion coefficients. All molecules were parameterized with GAFF force field.

Table S15: Calculated diffusion coefficients (**D**, in x10^{-10} m^2 s^{-1}), estimated errors (**error**), and correlation coefficients ($\mathbf{R^2}$) for each MA enantiomer obtained from different MD replicates of a simulation containing 5 molecules of (*R*)-MA, 5 molecules of (*S*)-MA, 50 molecules of (*R*)-BINOL and 4675 molecules of $CHCl_3$. All molecules were parameterized with GAFF force field.

Replicate	(*R*)-MA			(*S*)-MA		
	D	error	R^2	D	error	R^2
01	16.93	10.95	0.971	10.98	3.64	0.992
02	11.96	3.40	0.987	5.93	2.07	0.975
03	7.40	3.74	0.982	6.21	3.48	0.974
04	7.15	1.79	0.982	5.33	4.03	0.958
05	10.70	4.67	0.988	9.02	3.93	0.987
06	7.74	1.73	0.986	2.42	3.94	0.847
07	12.09	0.22	0.993	3.12	3.95	0.870
08	8.17	4.61	0.978	5.55	4.43	0.946
09	14.67	2.14	0.994	16.29	11.89	0.962
10	7.37	4.31	0.976	9.40	7.58	0.951
11	11.24	0.02	0.999	9.09	5.42	0.976
12	11.20	1.40	0.995	7.37	0.46	0.995
13	13.00	2.97	0.976	8.82	2.51	0.993
14	6.69	1.92	0.988	14.32	0.47	0.996
15	8.04	2.38	0.978	6.64	5.49	0.946
**Average**	10.29	0.18	1.000	8.03	0.02	0.999

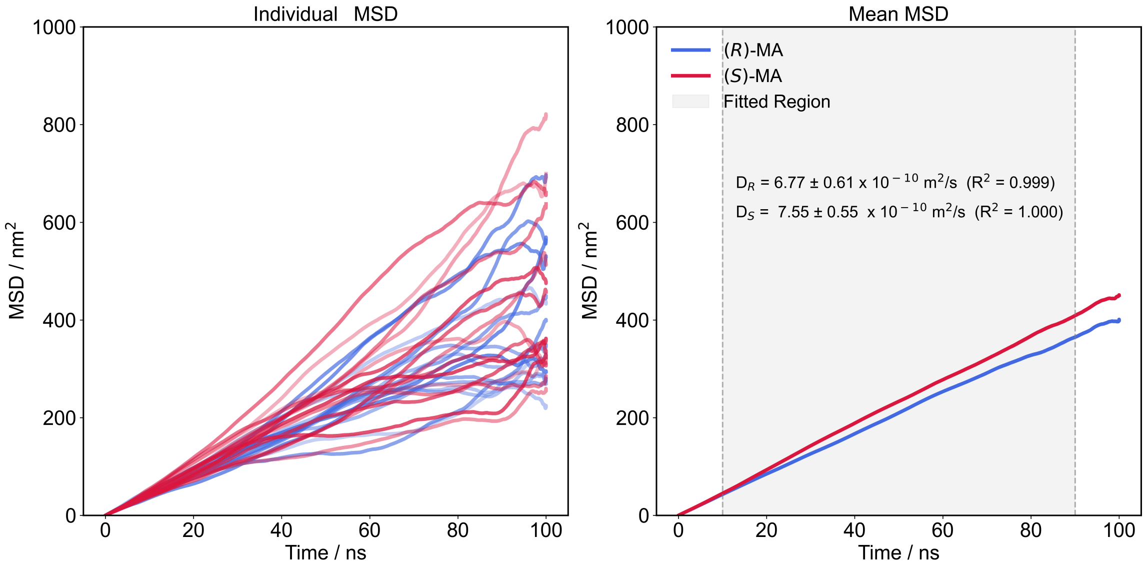

Figure S15: Mean square displacement (MSD) over time for (*R*)-MA (blue) and (*S*)-MA (red) obtained from 15 independent MD simulations of a cubic containing 5 molecules of (*R*)-MA, 5 molecules of (*S*)-MA, 50 molecules of (*R*)-BINOL and 4675 molecules of $CHCl_3$ (left panel). The right panel shows the mean MSD obtained from an average of all MSD curves plotted against the simulation time. The gray area indicates the region used for fitting to obtain the diffusion coefficients. All molecules were parameterized with OPLS-AA force field.

Table S16: Calculated diffusion coefficients (**D**, in x10^{-10} m^2 s^{-1}), estimated errors (**error**), and correlation coefficients (**R^2**) for each MA enantiomer obtained from different MD replicates of a simulation containing 5 molecules of (*R*)-MA, 5 molecules of (*S*)-MA, 50 molecules of (*R*)-BINOL and 4675 molecules of $CHCl_3$. All molecules were parameterized with OPLS-AA force field.

Replicate	(*R*)-MA			(*S*)-MA		
	D	error	R^2	D	error	R^2
01	3.67	0.54	0.959	4.38	8.00	0.824
02	8.67	1.42	0.997	12.53	2.81	0.992
03	4.41	8.11	0.823	7.35	3.20	0.977
04	4.77	6.44	0.883	11.97	8.26	0.965
05	6.42	1.60	0.972	3.16	0.52	0.981
06	4.75	1.54	0.973	7.65	4.25	0.981
07	6.96	0.38	0.979	4.54	6.07	0.894
08	5.41	3.22	0.962	10.99	3.75	0.990
09	7.35	0.65	0.997	13.64	0.95	0.994
10	3.87	3.16	0.902	5.41	4.35	0.951
11	9.44	5.63	0.974	8.64	5.99	0.970
12	11.42	1.28	0.996	4.99	3.35	0.968
13	11.53	1.89	0.993	9.10	0.80	0.994
14	7.06	1.41	0.996	2.92	2.58	0.895
15	5.86	0.65	0.994	6.00	5.09	0.952
**Average**	6.77	0.61	0.999	7.55	0.55	1.000

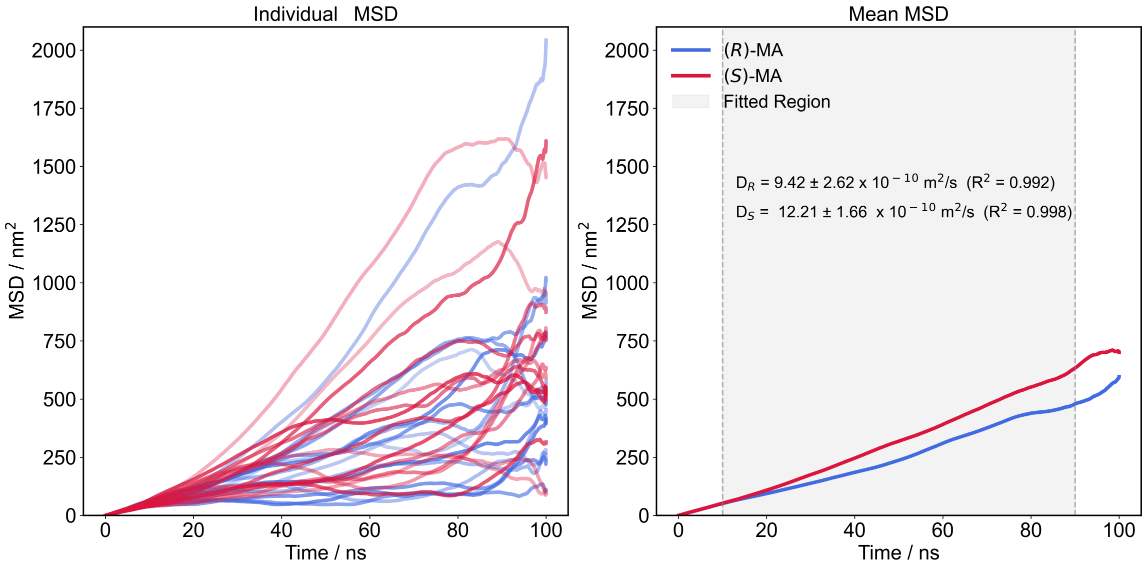

Figure S16: Mean square displacement (MSD) over time for (*R*)-MA (blue) and (*S*)-MA (red) obtained from 30 (15 for (*R*)-enantiomer and 15 for (*S*)-enantiomer) independent MD simulations of a cubic containing 1 molecule of (*R*)-MA or 1 molecule of (*S*)-MA, 5 molecules of (*R*)-BINOL and 468 molecules of $CHCl_3$ (left panel). The right panel shows the mean MSD obtained from an average of all MSD curves plotted against the simulation time. The gray area indicates the region used for fitting to obtain the diffusion coefficients. The enantiomer molecules were simulated in separate boxes and all molecules were parameterized with the GAFF force field.

Table S17: Calculated diffusion coefficients (**D**, in x10^{-10} m^2 s^{-1}), estimated errors (**error**), and correlation coefficients (**R^2**) for each MA enantiomer obtained from different MD replicates of a simulation containing 1 molecule of (*R*)-MA or 1 molecule of (*S*)-MA, 5 molecules of (*R*)-BINOL and 468 molecules of $CHCl_3$. The enantiomer molecules were simulated in separate boxes and all molecules were parameterized with the GAFF force field.

**Replicate**	**(*R*)-MA**			**(*S*)-MA**		
	**D**	**error**	**R^2**	**D**	**error**	**R^2**
01	15.73	0.16	0.974	26.67	4.94	0.988
02	6.08	4.43	0.891	38.14	7.36	0.987
03	6.72	3.91	0.854	3.05	12.99	0.451
04	34.14	18.97	0.974	4.91	2.76	0.932
05	0.95	3.79	0.110	3.12	6.40	0.292
06	3.83	0.51	0.887	12.76	5.46	0.968
07	8.39	0.19	0.957	7.21	5.62	0.956
08	17.39	1.43	0.987	4.61	6.66	0.717
09	17.38	6.45	0.981	11.97	2.94	0.989
10	0.86	3.22	0.441	16.07	0.67	0.985
11	8.95	0.68	0.975	10.16	1.65	0.980
12	2.13	1.06	0.928	23.87	6.11	0.989
13	12.26	11.57	0.927	0.98	2.51	0.243
14	1.29	1.07	0.709	9.00	11.17	0.901
15	5.23	2.11	0.978	10.70	4.55	0.952
**Average**	9.42	2.62	0.992	12.21	1.66	0.998

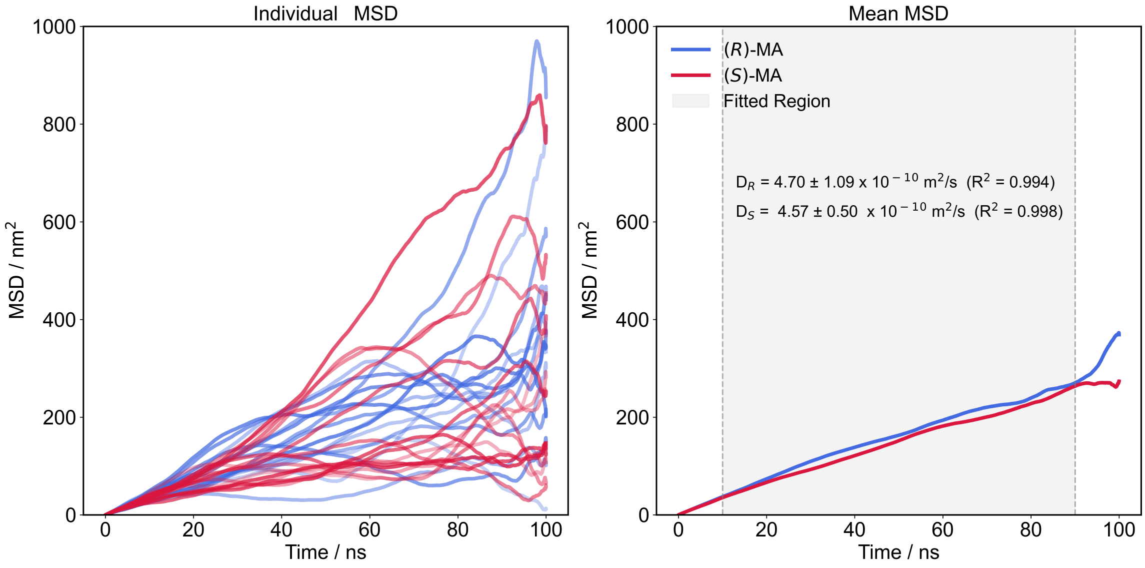

Figure S17: Mean square displacement (MSD) over time for (*R*)-MA (blue) and (*S*)-MA (red) obtained from 30 (15 for (*R*)-enantiomer and 15 for (*S*)-enantiomer) independent MD simulations of a cubic containing 1 molecule of (*R*)-MA or 1 molecule of (*S*)-MA, 5 molecules of (*R*)-BINOL and 468 molecules of $CHCl_3$ (left panel). The right panel shows the mean MSD obtained from an average of all MSD curves plotted against the simulation time. The gray area indicates the region used for fitting to obtain the diffusion coefficients. The enantiomer molecules were simulated in separate boxes and all molecules were parameterized with the OPLS-AA force field.

Table S18: Calculated diffusion coefficients (**D**, in x10^{-10} m^2 s^{-1}), estimated errors (**error**), and correlation coefficients (**R^2**) for each MA enantiomer obtained from different MD replicates of a simulation containing 1 molecule of (*R*)-MA or 1 molecule of (*S*)-MA, 5 molecules of (*R*)-BINOL and 468 molecules of $CHCl_3$. The enantiomer molecules were simulated in separate boxes and all molecules were parameterized with the OPLS-AA force field.

**Replicate**	**(*R*)-MA**			**(*S*)-MA**		
	**D**	**error**	**R^2**	**D**	**error**	**R^2**
01	1.65	5.64	0.449	1.77	2.52	0.574
02	6.65	16.50	0.668	1.43	1.53	0.783
03	4.94	0.95	0.989	2.61	0.24	0.844
04	4.64	12.77	0.642	2.78	5.09	0.725
05	5.30	0.65	0.966	2.00	3.12	0.439
06	1.72	4.14	0.710	4.91	16.67	0.511
07	3.69	0.96	0.985	9.98	2.10	0.984
08	5.78	3.85	0.957	0.99	7.34	0.204
09	13.12	12.66	0.936	9.68	2.22	0.986
10	2.95	6.77	0.713	6.79	0.32	0.979
11	-0.74	5.98	0.120	3.09	3.98	0.750
12	4.13	10.64	0.684	1.86	2.23	0.878
13	5.36	6.06	0.909	2.80	0.78	0.819
14	3.83	3.72	0.843	16.06	5.73	0.983
15	7.42	5.16	0.961	1.77	2.42	0.883
**Average**	4.70	1.09	0.994	4.57	0.50	0.998

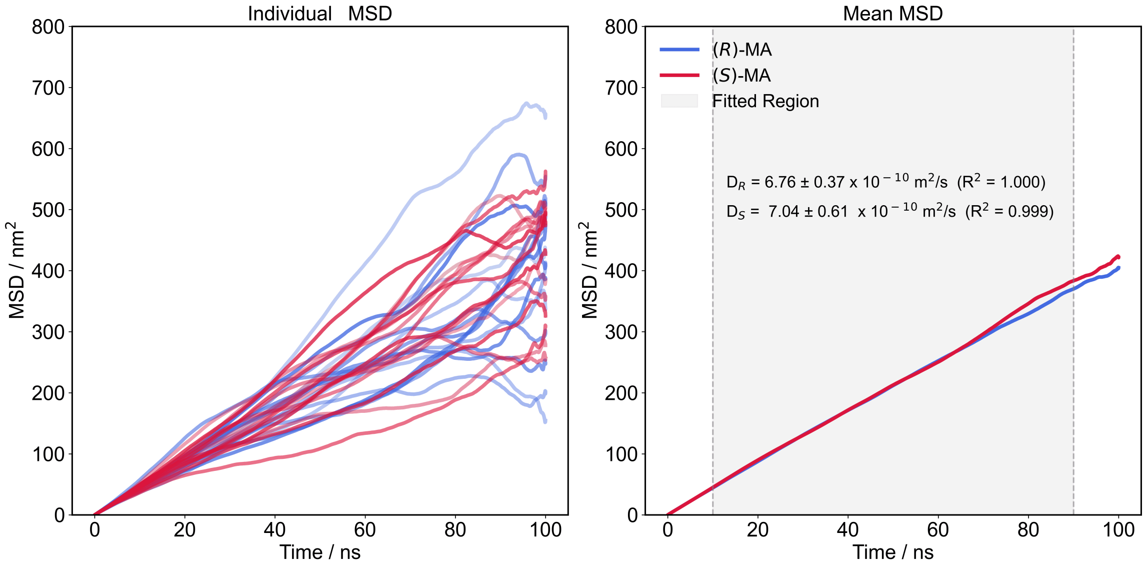

Figure S18: Mean square displacement (MSD) over time for (*R*)-MA (blue) and (*S*)-MA (red) obtained from 30 (15 for (*R*)-enantiomer and 15 for (*S*)-enantiomer) independent MD simulations of a cubic containing 5 molecules of (*R*)-MA or 5 molecules of (*S*)-MA, 25 molecules of (*R*)-BINOL and 1169 molecules of $CHCl_3$ (left panel). The right panel shows the mean MSD obtained from an average of all MSD curves plotted against the simulation time. The gray area indicates the region used for fitting to obtain the diffusion coefficients. The enantiomer molecules were simulated in separate boxes and all molecules were parameterized with the GAFF force field.

Table S19: Calculated diffusion coefficients (**D**, in x10^{-10} m^2 s^{-1}), estimated errors (**error**), and correlation coefficients (**R^2**) for each MA enantiomer obtained from different MD replicates of a simulation containing 5 molecules of (*R*)-MA or 5 molecules of (*S*)-MA, 25 molecules of (*R*)-BINOL and 1169 molecules of $CHCl_3$. The enantiomer molecules were simulated in separate boxes and all molecules were parameterized with the GAFF force field.

**Replicate**	**(*R*)-MA**			**(*S*)-MA**		
	**D**	**error**	**R^2**	**D**	**error**	**R^2**
01	8.50	0.47	0.992	8.86	1.30	0.996
02	13.22	2.17	0.992	10.04	6.58	0.973
03	7.57	3.96	0.979	5.98	1.34	0.997
04	4.94	3.64	0.949	7.65	0.11	0.994
05	5.95	3.57	0.973	3.49	1.65	0.946
06	3.69	4.36	0.912	7.81	1.10	0.986
07	9.81	3.92	0.981	4.85	5.18	0.912
08	5.44	3.21	0.937	5.88	1.22	0.986
09	5.18	1.87	0.961	8.16	4.53	0.976
10	6.38	2.30	0.980	3.52	2.13	0.970
11	4.82	5.74	0.905	10.18	5.87	0.978
12	5.81	2.44	0.980	7.72	2.27	0.992
13	4.95	1.41	0.987	5.27	1.41	0.992
14	5.98	3.66	0.970	6.67	0.78	0.992
15	9.11	0.46	0.992	9.56	3.30	0.982
**Average**	6.76	0.37	1.000	7.04	0.61	0.999

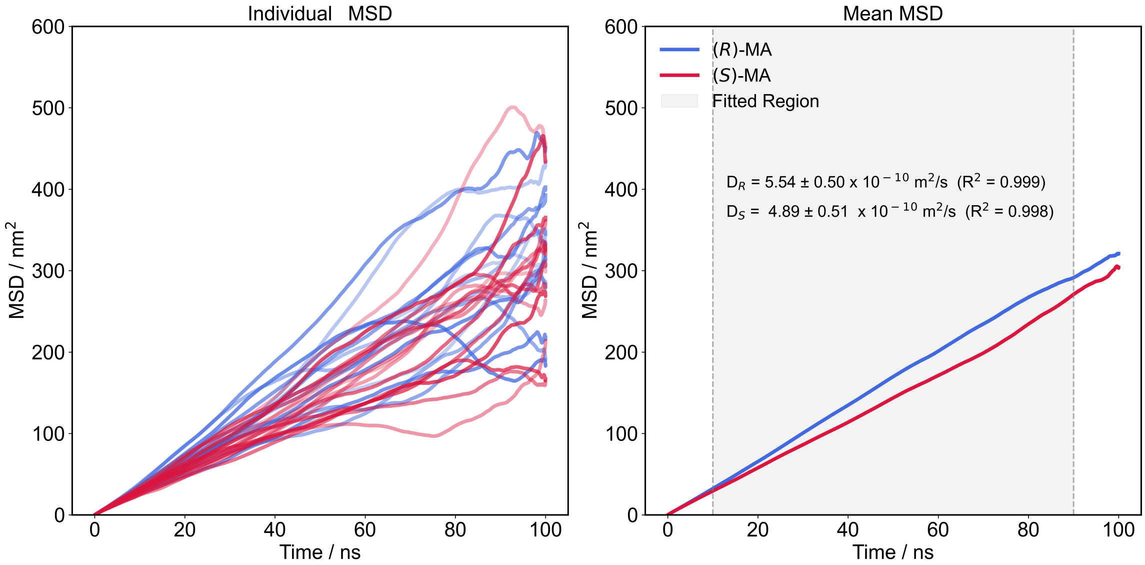

Figure S19: Mean square displacement (MSD) over time for (*R*)-MA (blue) and (*S*)-MA (red) obtained from 30 (15 for (*R*)-enantiomer and 15 for (*S*)-enantiomer) independent MD simulations of a cubic containing 5 molecules of (*R*)-MA or 5 molecules of (*S*)-MA, 25 molecules of (*R*)-BINOL and 1169 molecules of $CHCl_3$ (left panel). The right panel shows the mean MSD obtained from an average of all MSD curves plotted against the simulation time. The gray area indicates the region used for fitting to obtain the diffusion coefficients. The enantiomer molecules were simulated in separate boxes and all molecules were parameterized with the OPLS-AA force field.

Table S20: Calculated diffusion coefficients (**D**, in x10^{-10} m^2 s^{-1}), estimated errors (**error**), and correlation coefficients (**R^2**) for each MA enantiomer obtained from different MD replicates of a simulation containing 5 molecules of (*R*)-MA or 5 molecules of (*S*)-MA, 25 molecules of (*R*)-BINOL and 1169 molecules of $CHCl_3$. The enantiomer molecules were simulated in separate boxes and all molecules were parameterized with the OPLS-AA force field.

**Replicate**	**(*R*)-MA**			**(*S*)-MA**		
	**D**	**error**	**R^2**	**D**	**error**	**R^2**
01	6.25	1.26	0.995	5.35	1.25	0.989
02	4.27	1.43	0.970	8.79	5.07	0.969
03	9.37	1.06	0.983	5.54	0.22	0.994
04	6.48	1.95	0.976	5.61	2.31	0.979
05	5.40	0.61	0.998	1.60	3.07	0.746
06	5.20	0.19	0.979	5.47	1.72	0.992
07	4.01	2.89	0.960	5.90	1.08	0.995
08	5.11	0.63	0.992	2.68	1.84	0.968
09	3.11	0.09	0.929	5.72	0.93	0.994
10	6.65	0.60	0.998	4.15	1.40	0.988
11	6.39	1.13	0.996	4.58	1.08	0.981
12	8.56	1.32	0.993	5.10	0.74	0.995
13	3.61	1.15	0.989	3.19	0.72	0.980
14	4.88	0.17	0.999	6.30	0.70	0.994
15	3.80	9.00	0.705	3.34	0.08	0.992
**Average**	5.54	0.50	0.999	4.89	0.51	0.998

4.2 Number of replicates and Diffusion Coefficients

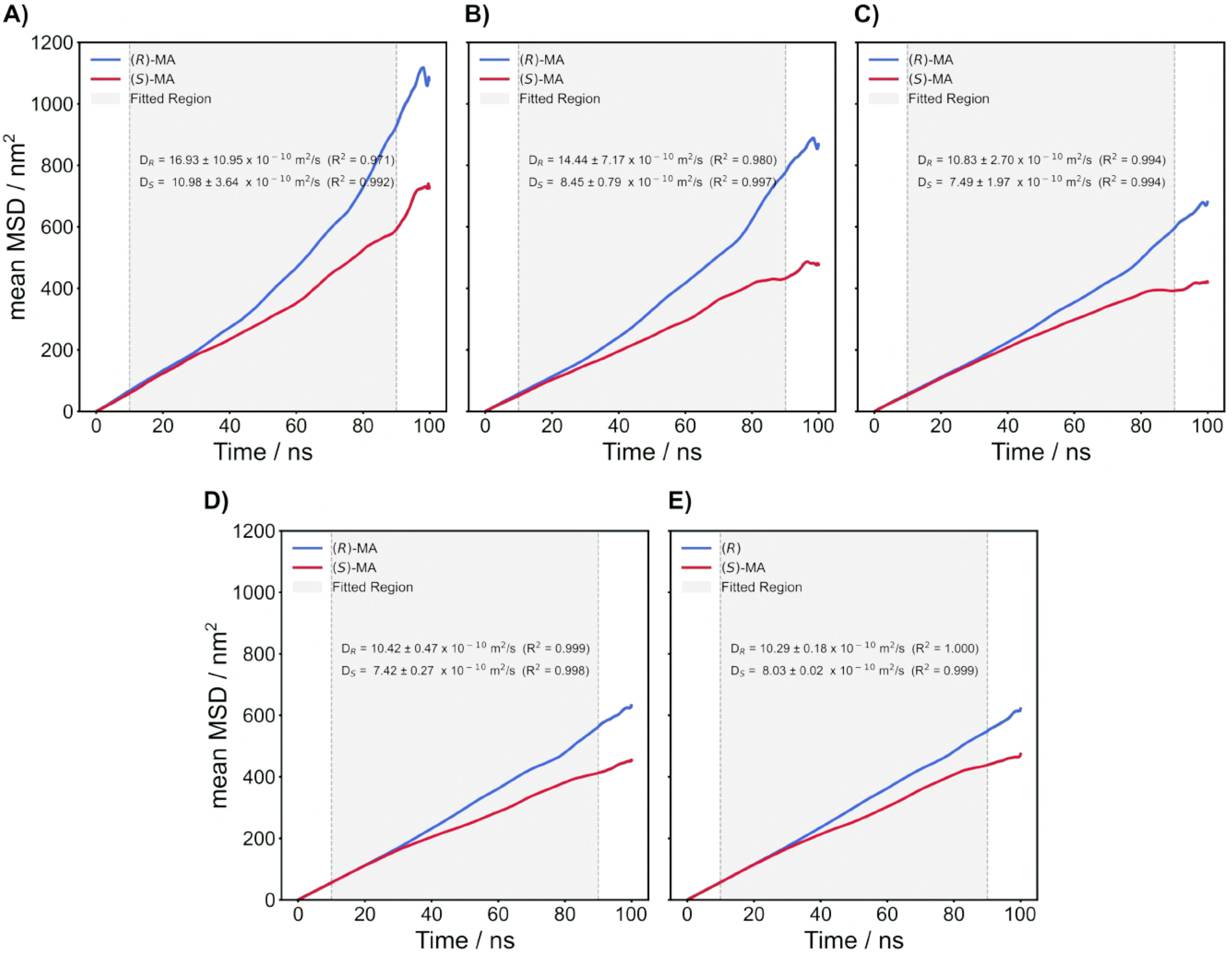

Figure S20: Mean MSD over time for (*R*)-MA (blue) and (*S*)-MA (red) obtained from **A)** 1, **B)** 2, **C)** 5, **D)** 10, and **E)** 15 independent MD simulations of a cubic containing 5 molecules of (*R*)-MA or 5 molecules of (*S*)-MA, 50 molecules of (*R*)-BINOL and 4675 molecules of $CHCl_3$. The gray area indicates the region used for fitting to obtain the diffusion coefficients. All molecules were parameterized with the GAFF force field.

Table S21: Calculated diffusion coefficients (**D**, in x10^{-10} m^2 s^{-1}), estimated errors (**error**), and correlation coefficients (**R^2**) for each MA enantiomer obtained from different MD replicates of a simulation containing 5 molecules of (*R*)-MA or 5 molecules of (*S*)-MA, 50 molecules of (*R*)-BINOL and 4675 molecules of $CHCl_3$. All molecules were parameterized with GAFF force field.

**Number of replicates**	**(*R*)-MA**			**(*S*)-MA**		
	**D**	**error**	**R^2**	**D**	**error**	**R^2**
1	16.93	10.95	0.971	10.98	3.64	0.992
2	14.44	7.17	0.980	8.45	0.79	0.997
5	10.83	2.70	0.994	7.49	1.97	0.994
10	10.42	0.47	0.999	7.42	0.27	0.998
15	10.29	0.18	1.000	8.03	0.02	0.999

4.3 Convergence of Diffusion Coefficients with Simulation Lengths

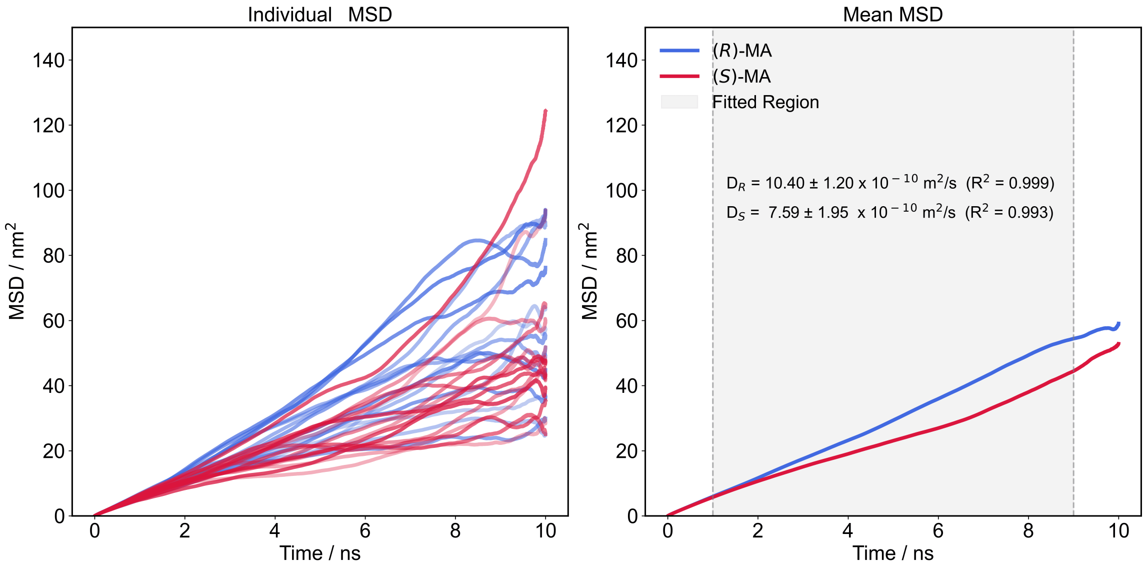

Figure S21: Mean square displacement (MSD) over 10 ns for (*R*)-MA (blue) and (*S*)-MA (red) obtained from 15 independent MD simulations of a cubic containing 5 molecules of (*R*)-MA, 5 molecules of (*S*)-MA, 50 molecules of (*R*)-BINOL and 4675 molecules of $CHCl_3$ (left panel). The right panel shows the mean MSD obtained from an average of all MSD curves plotted against the simulation time. The gray area indicates the region used for fitting to obtain the diffusion coefficients. All molecules were parameterized with GAFF force field.

Table S22: Calculated diffusion coefficients (**D**, in x10^{-10} m^2 s^{-1}), estimated errors (**error**), and correlation coefficients (**R^2**) for each MA enantiomer obtained from different MD replicates of a simulation containing 5 molecules of (*R*)-MA, 5 molecules of (*S*)-MA, 50 molecules of (*R*)-BINOL and 4675 molecules of $CHCl_3$. All molecules were parameterized with GAFF force field. Values are relative to a simulation time of 10 ns.

**Replicate**	**(*R*)-MA**			**(*S*)-MA**		
	**D**	**error**	**R^2**	**D**	**error**	**R^2**
01	10.67	7.97	0.965	12.06	9.21	0.956
02	6.14	5.48	0.950	3.41	1.37	0.967
03	7.66	1.66	0.992	4.51	1.60	0.957
04	5.15	2.15	0.984	7.40	5.11	0.969
05	9.32	3.16	0.989	11.73	5.93	0.983
06	14.93	10.27	0.957	4.85	2.04	0.935
07	10.55	3.28	0.989	8.65	6.10	0.962
08	12.27	4.12	0.979	9.09	0.57	0.997
09	3.59	3.17	0.940	8.45	4.66	0.967
10	9.90	2.92	0.977	3.14	5.69	0.748
11	7.43	1.18	0.990	4.33	3.34	0.924
12	18.02	7.58	0.984	6.96	2.99	0.985
13	9.70	1.27	0.998	15.45	8.30	0.970
14	13.71	2.81	0.995	6.96	2.95	0.972
15	16.90	3.01	0.994	6.82	3.77	0.973
**Average**	10.40	1.20	0.999	7.59	1.95	0.993

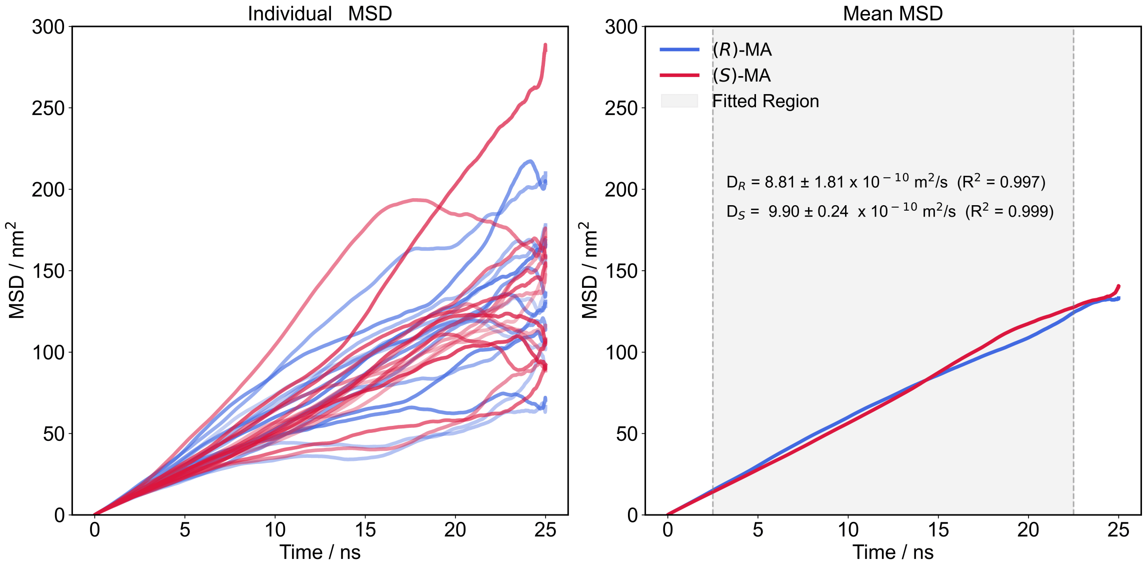

Figure S22: Mean square displacement (MSD) over 25 ns for (*R*)-MA (blue) and (*S*)-MA (red) obtained from 15 independent MD simulations of a cubic containing 5 molecules of (*R*)-MA, 5 molecules of (*S*)-MA, 50 molecules of (*R*)-BINOL and 4675 molecules of $CHCl_3$ (left panel). The right panel shows the mean MSD obtained from an average of all MSD curves plotted against the simulation time. The gray area indicates the region used for fitting to obtain the diffusion coefficients. All molecules were parameterized with GAFF force field.

Table S23: Calculated diffusion coefficients (**D**, in x10^{-10} m^2 s^{-1}), estimated errors (**error**), and correlation coefficients (**R^2**) for each MA enantiomer obtained from different MD replicates of a simulation containing 5 molecules of (*R*)-MA, 5 molecules of (*S*)-MA, 50 molecules of (*R*)-BINOL and 4675 molecules of $CHCl_3$. All molecules were parameterized with GAFF force field. Values are relative to a simulation time of 25 ns.

Replicate	(*R*)-MA			(*S*)-MA		
	D	error	R^2	D	error	R^2
01	9.11	6.45	0.967	8.72	0.85	0.998
02	9.05	5.30	0.972	8.99	2.47	0.987
03	3.11	2.24	0.833	8.46	3.20	0.973
04	3.16	0.51	0.924	9.44	0.07	0.998
05	11.87	1.92	0.995	10.44	3.38	0.979
06	10.79	2.21	0.995	3.86	1.50	0.901
07	6.91	4.28	0.971	9.22	1.11	0.994
08	14.77	5.30	0.986	16.29	15.88	0.913
09	9.89	2.74	0.990	11.94	2.53	0.995
10	9.44	1.44	0.995	9.10	1.04	0.976
11	10.31	1.75	0.997	4.00	2.31	0.973
12	11.74	2.88	0.977	10.74	3.79	0.987
13	4.09	4.22	0.922	18.67	12.54	0.972
14	6.78	1.84	0.979	10.35	2.48	0.984
15	11.08	2.28	0.995	8.25	1.62	0.996
**Average**	8.81	1.81	0.997	9.90	0.24	0.999

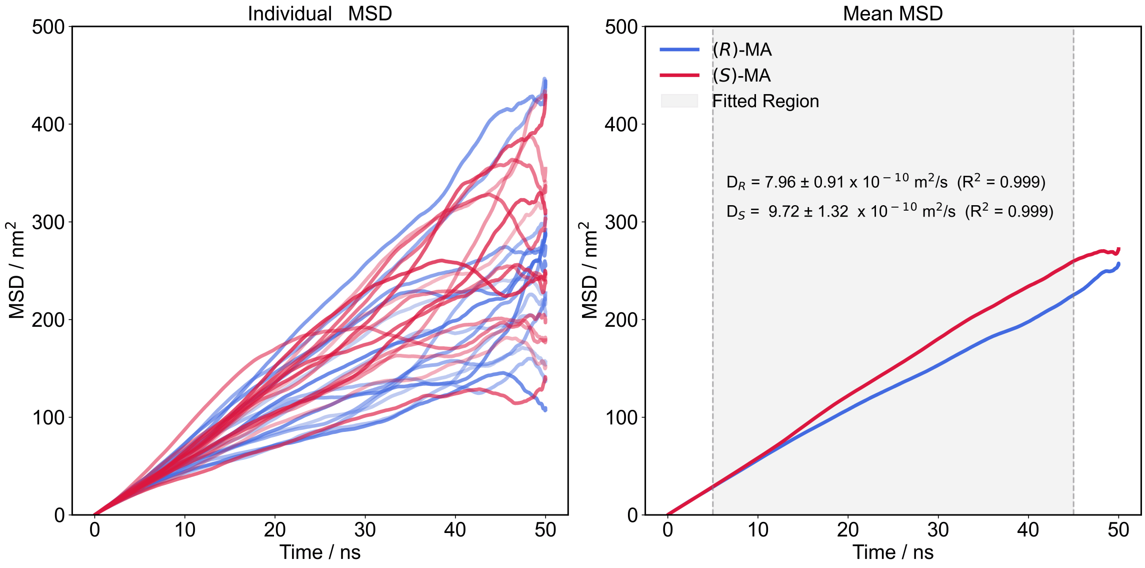

Figure S23: Mean square displacement (MSD) over 50 ns for (*R*)-MA (blue) and (*S*)-MA (red) obtained from 15 independent MD simulations of a cubic containing 5 molecules of (*R*)-MA, 5 molecules of (*S*)-MA, 50 molecules of (*R*)-BINOL and 4675 molecules of $CHCl_3$ (left panel). The right panel shows the mean MSD obtained from an average of all MSD curves plotted against the simulation time. The gray area indicates the region used for fitting to obtain the diffusion coefficients. All molecules were parameterized with GAFF force field.

Table S24: Calculated diffusion coefficients (**D**, in x10^{-10} m^2 s^{-1}), estimated errors (**error**), and correlation coefficients (**R^2**) for each MA enantiomer obtained from different MD replicates of a simulation containing 5 molecules of (*R*)-MA, 5 molecules of (*S*)-MA, 50 molecules of (*R*)-BINOL and 4675 molecules of $CHCl_3$. All molecules were parameterized with GAFF force field. Values are relative to a simulation time of 50 ns.

Replicate	(*R*)-MA			(*S*)-MA		
	D	error	R^2	D	error	R^2
01	9.04	2.95	0.987	10.63	4.06	0.985
02	5.21	0.37	0.995	6.15	2.70	0.985
03	7.63	2.52	0.978	13.95	1.09	0.993
04	5.71	3.69	0.938	6.66	4.35	0.967
05	4.02	8.56	0.766	10.63	8.76	0.932
06	8.79	10.81	0.901	7.60	1.96	0.993
07	5.92	0.39	0.980	9.22	1.94	0.988
08	13.41	1.48	0.998	4.95	14.17	0.635
09	6.58	0.60	0.970	14.35	3.65	0.995
10	10.63	4.28	0.985	13.87	0.64	0.993
11	15.71	0.81	0.997	4.70	1.62	0.987
12	8.67	7.67	0.941	8.68	4.49	0.979
13	5.32	2.34	0.972	13.95	8.21	0.974
14	4.59	1.31	0.988	10.03	5.93	0.973
15	8.13	2.90	0.991	10.44	10.64	0.920
**Average**	7.96	0.91	0.999	9.72	1.32	0.999

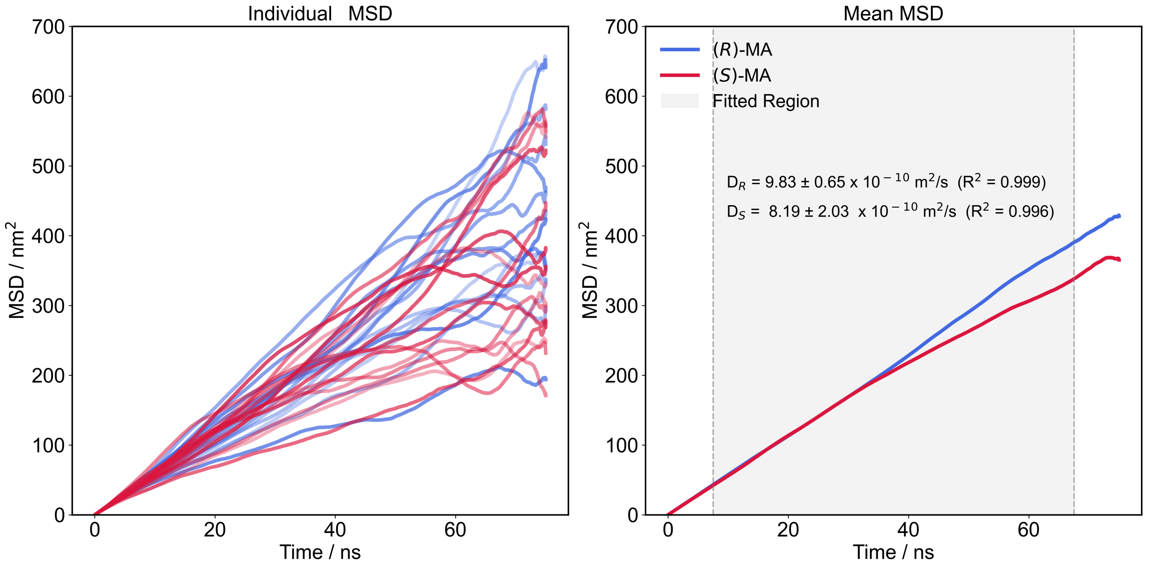

Figure S24: Mean square displacement (MSD) over 75 ns for (*R*)-MA (blue) and (*S*)-MA (red) obtained from 15 independent MD simulations of a cubic containing 5 molecules of (*R*)-MA, 5 molecules of (*S*)-MA, 50 molecules of (*R*)-BINOL and 4675 molecules of $CHCl_3$ (left panel). The right panel shows the mean MSD obtained from an average of all MSD curves plotted against the simulation time. The gray area indicates the region used for fitting to obtain the diffusion coefficients. All molecules were parameterized with GAFF force field.

Table S25: Calculated diffusion coefficients (**D**, in x10^{-10} m^2 s^{-1}), estimated errors (**error**), and correlation coefficients (**R^2**) for each MA enantiomer obtained from different MD replicates of a simulation containing 5 molecules of (*R*)-MA, 5 molecules of (*S*)-MA, 50 molecules of (*R*)-BINOL and 4675 molecules of $CHCl_3$. All molecules were parameterized with GAFF force field. Values are relative to a simulation time of 75 ns.

**Replicate**	**(*R*)-MA**			**(*S*)-MA**		
	**D**	**error**	**R^2**	**D**	**error**	**R^2**
01	12.91	9.78	0.957	12.23	5.01	0.988
02	9.01	2.60	0.994	5.16	4.07	0.950
03	7.67	2.23	0.990	10.18	3.71	0.987
04	8.00	4.85	0.947	5.77	4.10	0.952
05	8.37	0.67	0.991	12.20	3.60	0.993
06	7.36	3.13	0.976	5.80	2.01	0.992
07	12.39	5.43	0.981	5.16	4.16	0.951
08	11.57	6.88	0.975	3.41	12.67	0.508
09	11.36	0.56	0.998	12.90	0.63	0.997
10	11.05	2.39	0.977	7.49	3.88	0.977
11	13.95	1.13	0.995	4.85	0.98	0.996
12	9.71	1.59	0.990	6.94	0.69	0.993
13	13.18	7.57	0.977	10.75	7.45	0.955
14	4.46	0.56	0.982	11.00	1.88	0.992
15	6.47	5.58	0.948	9.04	9.73	0.920
**Average**	9.83	0.65	0.999	8.19	2.03	0.996

4.4 Calculated Diffusion Coefficients for (*R*/*S*)-MA, (*R*/*S*)-BINOL and $CHCl_3$

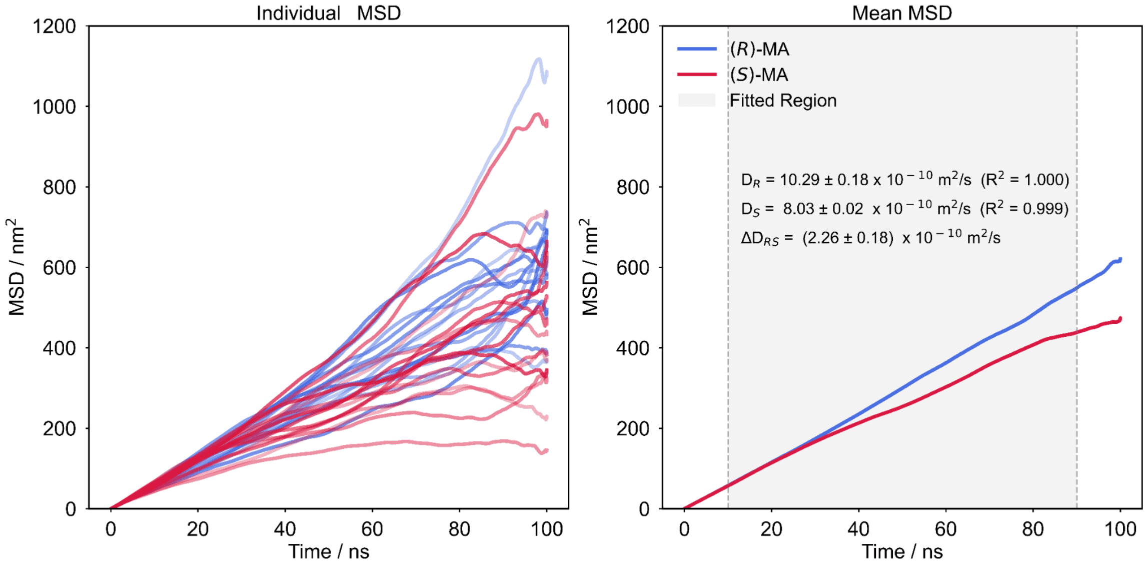

Figure S25: Mean square displacement (MSD) as a function of time for (*R*)-MA (blue) and (*S*)-MA (red) of each 15 independent MD simulations (left panel). The right panel shows the mean MSD of all simulations with shaded gray regions indicating the fitting range used to determine the diffusion coefficients. Both panels display MSD curves for (*R*/*S*)-MA in the presence of (*R*)-BINOL. All molecules were parameterized with GAFF force field.

Table S26: Calculated diffusion coefficients (**D**, in x10^{-10} m^2 s^{-1}), estimated errors (**error**), and correlation coefficients (**R^2**) for different replicates of each molecular species in the simulation box: (*R*)-MA, (*S*)-MA, (*R*)-BINOL, and $CHCl_3$. All molecules were parameterized with GAFF force field.

Replicate	(*R*)-MA			(*S*)-MA			(*R*)-BINOL			$CHCl_3$		
	D	error	R^2	D	error	R^2	D	error	R^2	D	error	R^2
01	16.93	10.95	0.971	10.98	3.64	0.992	6.58	0.95	0.998	19.15	0.43	1.000
02	11.96	3.40	0.987	5.93	2.07	0.975	7.11	0.15	1.000	19.20	0.06	1.000
03	7.40	3.74	0.982	6.21	3.48	0.974	6.60	0.95	0.998	19.16	0.27	1.000
04	7.15	1.79	0.982	5.33	4.03	0.958	5.58	1.78	0.992	19.49	0.29	1.000
05	10.70	4.67	0.988	9.02	3.93	0.987	5.71	0.56	0.999	18.66	0.46	1.000
06	7.74	1.73	0.986	2.42	3.94	0.847	5.73	0.50	0.998	19.45	0.08	1.000
07	12.09	0.22	0.993	3.12	3.95	0.870	7.97	1.20	0.998	19.60	0.25	1.000
08	8.17	4.61	0.978	5.55	4.43	0.946	6.77	0.80	0.999	19.10	0.15	1.000
09	14.67	2.14	0.994	16.29	11.89	0.962	5.96	0.82	0.997	20.68	0.56	1.000
10	7.37	4.31	0.976	9.40	7.58	0.951	7.63	1.10	0.999	19.48	0.08	1.000
11	11.24	0.02	0.999	9.09	5.42	0.976	7.29	0.82	0.999	19.37	0.22	1.000
12	11.20	1.40	0.995	7.37	0.46	0.995	7.85	0.54	0.999	20.11	0.30	1.000
13	13.00	2.97	0.976	8.82	2.51	0.993	7.62	0.02	1.000	19.22	0.58	1.000
14	6.69	1.92	0.988	14.32	0.47	0.996	6.20	0.47	0.999	19.91	0.05	1.000
15	8.04	2.38	0.978	6.64	5.49	0.946	7.31	0.06	1.000	18.67	0.12	1.000
**Average**	10.29	0.18	1.000	8.03	0.02	0.999	6.79	0.22	1.000	19.42	0.09	1.000

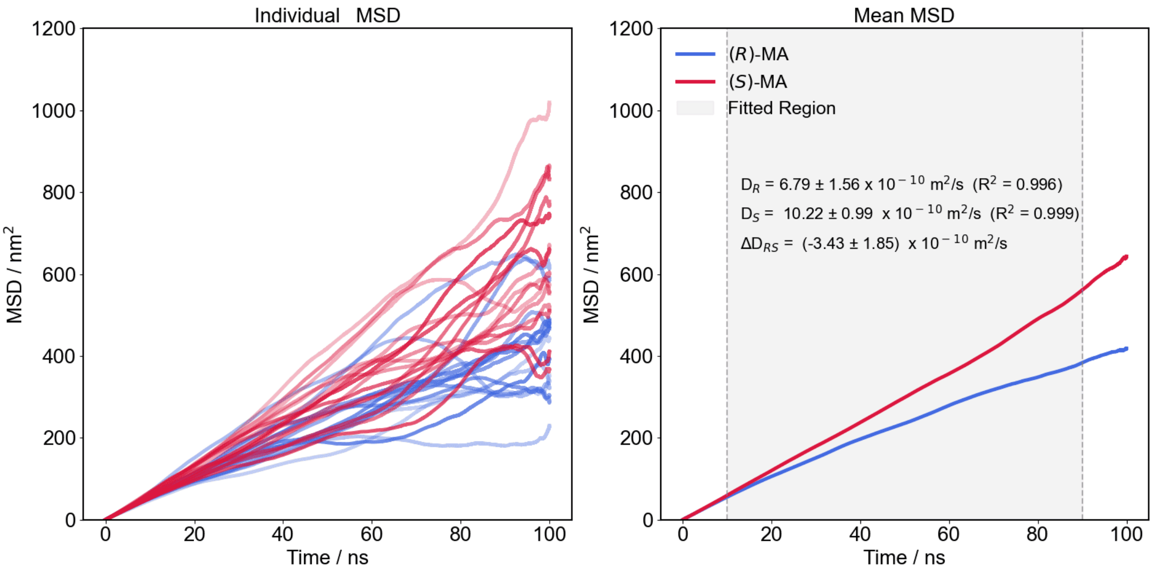

Figure S26: Mean square displacement (MSD) as a function of time for (*R*)-MA (blue) and (*S*)-MA (red) of each 15 independent MD simulations (left panel). The right panel shows the mean MSD of all simulations with shaded gray regions indicating the fitting range used to determine the diffusion coefficients. Both panels display MSD curves for (*R*/*S*)-MA in the presence of (*S*)-BINOL. All molecules were parameterized with GAFF force field.

Table S27: Calculated diffusion coefficients (**D**, in x10^{-10} m^2 s^{-1}), estimated errors (**error**), and R-squared values (**R^2**) for different replicates of each molecular species in the simulation box: (*R*)-MA, (*S*)-MA, (*S*)-BINOL, and $CHCl_3$. All molecules were parameterized with GAFF force field.

Replicate	(*R*)-MA			(*S*)-MA			(*S*)-BINOL			$CHCl_3$		
	D	error	R^2	D	error	R^2	D	error	R^2	D	error	R^2
01	6.11	1.72	0.992	15.28	0.28	0.998	8.24	1.69	1.00	19.27	0.25	1.00
02	5.92	3.57	0.967	12.22	5.65	0.946	5.91	2.19	0.99	19.67	0.33	1.00
03	6.15	1.52	0.990	8.37	6.04	0.960	7.11	0.53	1.00	19.87	0.12	1.00
04	1.93	7.20	0.503	10.53	1.71	0.991	7.78	0.19	1.00	19.61	0.08	1.00
05	6.60	14.07	0.708	8.01	5.67	0.965	7.35	0.68	1.00	19.63	0.36	1.00
06	13.28	2.26	0.993	8.44	1.18	0.982	7.23	0.84	1.00	19.73	0.04	1.00
07	7.64	2.37	0.991	14.75	1.44	0.997	7.52	0.36	1.00	19.41	0.18	1.00
08	7.38	0.85	0.997	8.43	0.63	0.994	7.86	0.05	1.00	19.38	0.51	1.00
09	8.99	8.90	0.910	8.25	5.29	0.962	8.39	0.46	1.00	19.79	0.72	1.00
10	6.01	7.62	0.897	10.71	6.56	0.967	6.14	1.93	0.99	19.55	0.04	1.00
11	7.27	0.10	0.998	9.84	11.37	0.895	8.07	0.02	1.00	20.19	0.05	1.00
12	5.93	5.01	0.952	7.41	3.31	0.979	8.66	0.64	1.00	19.56	0.12	1.00
13	6.77	3.62	0.973	11.60	1.90	0.997	5.83	0.87	1.00	19.66	0.23	1.00
14	7.65	1.29	0.996	6.52	4.09	0.928	6.61	1.03	1.00	19.39	0.01	1.00
15	4.29	0.26	0.924	12.98	7.46	0.973	7.15	0.41	1.00	19.37	0.02	1.00
**Average**	6.79	1.56	0.996	10.22	0.99	0.999	7.32	0.26	1.00	19.61	0.02	1.00

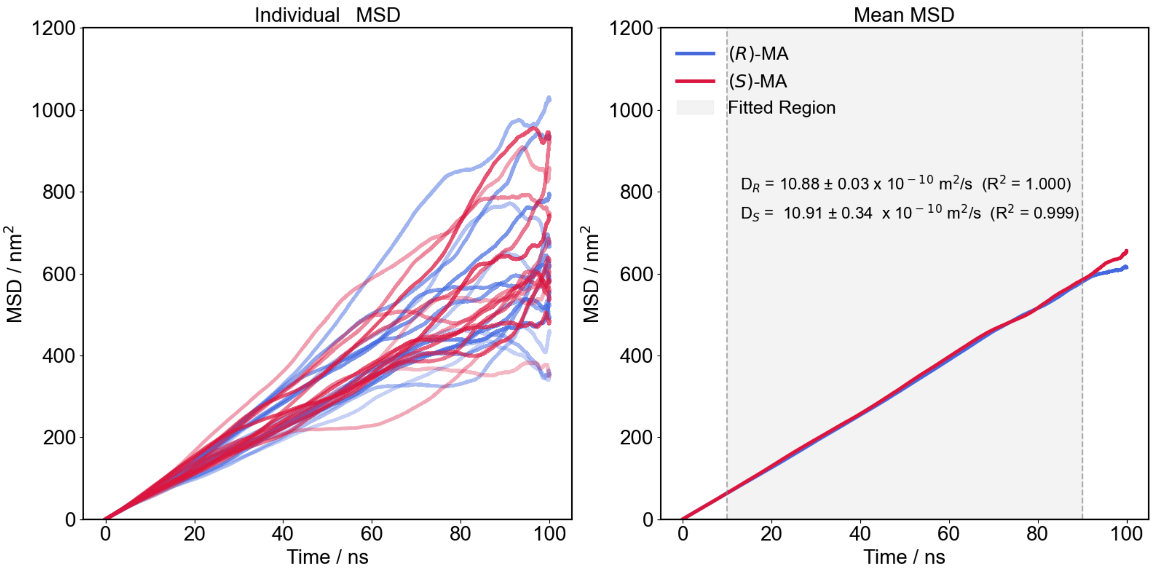

Figure S27: Mean square displacement (MSD) as a function of time for (*R*)-MA (blue) and (*S*)-MA (red) of each 15 independent MD simulations (left panel). The right panel shows the mean MSD of all simulations with shaded gray regions indicating the fitting range used to determine the diffusion coefficients. Both panels display MSD curves for (*R*/*S*)-MA in the absence of BINOL. All molecules were parameterized with GAFF force field.

Table S28: Calculated diffusion coefficients (**D**, in x10^{-10} m^2 s^{-1}), estimated errors (**error**), and R-squared values (**R^2**) for different replicates of each molecular species in the simulation box: (*R*)-MA, (*S*)-MA, and $CHCl_3$. All molecules were parameterized with GAFF force field.

Replicate	(*R*)-MA			(*S*)-MA			$CHCl_3$		
	D	error	R^2	D	error	R^2	D	error	R^2
01	8.08	1.02	0.997	7.12	8.31	0.904	20.97	0.10	1.000
02	9.12	0.86	0.982	11.63	2.81	0.977	21.36	0.13	1.000
03	11.30	8.31	0.962	6.07	3.71	0.915	21.44	0.04	1.000
04	14.82	5.82	0.985	16.19	3.61	0.994	21.52	0.23	1.000
05	7.07	4.46	0.959	10.38	0.05	0.998	20.85	0.37	1.000
06	8.25	3.52	0.987	10.23	11.10	0.913	21.23	0.67	1.000
07	19.59	3.91	0.991	16.33	3.62	0.995	21.56	0.21	1.000
08	8.90	0.78	0.996	9.80	3.01	0.990	21.35	0.48	1.000
09	8.39	4.03	0.973	12.92	1.70	0.995	21.95	0.02	1.000
10	14.89	0.81	0.994	9.04	0.50	0.990	20.76	0.03	1.000
11	10.41	5.05	0.982	7.71	3.88	0.974	21.60	0.30	1.000
12	11.76	2.21	0.986	9.90	1.78	0.997	21.57	0.55	1.000
13	12.53	2.06	0.996	9.37	3.73	0.984	21.15	0.25	1.000
14	9.08	0.56	0.998	15.93	10.35	0.967	21.08	0.06	1.000
15	9.03	2.68	0.981	11.01	7.35	0.959	21.60	0.10	1.000
**Average**	10.88	0.03	1.000	10.91	0.34	0.999	21.33	0.07	1.000

4.5 Calculated Diastereomeric Complex Lifetime

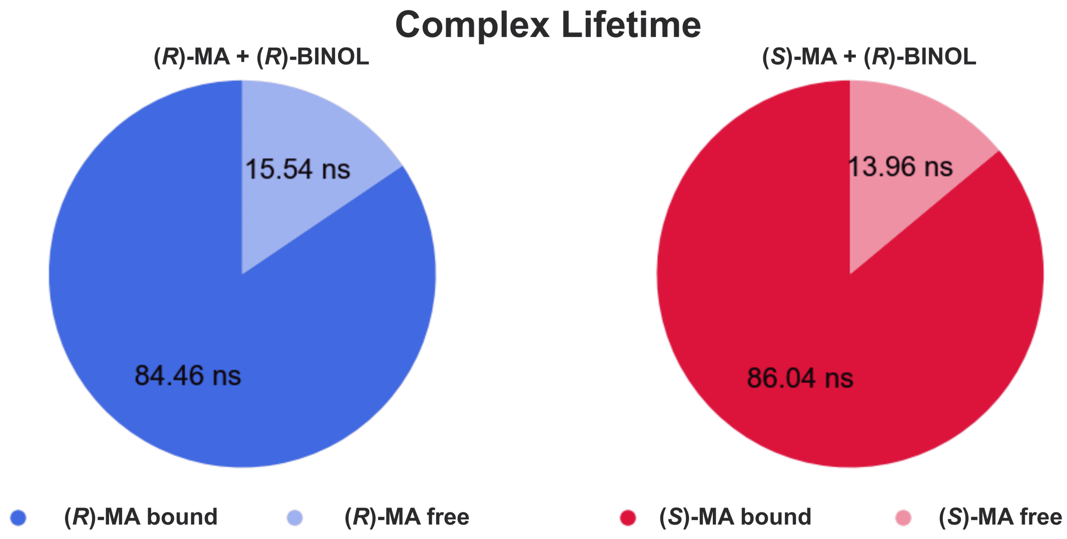

Figure S28: Distribution of complex lifetimes for (*R*)-MA (left) and (*S*)-MA (right) in either the bound or free state with respect to (*R*)-BINOL.

Table S29: Complex lifetimes (in ns) for the diastereoisomeric complexes formed between (*R*)-BINOL and (*R*)-MA, and (*R*)-BINOL and (*S*)-MA, for each of the 15 MD replicates.

**Replicate**	**Complex Lifetime**	
	**(*R*)-MA + (*R*)-BINOL**	**(*S*)-MA + (*R*)-BINOL**
01	85.81	84.94
02	85.09	83.88
03	82.80	87.45
04	84.69	89.26
05	86.13	85.10
06	82.56	86.88
07	82.44	86.39
08	85.93	84.56
09	85.19	87.89
10	83.80	85.12
11	85.28	83.45
12	81.86	84.54
13	83.97	85.45
14	86.06	88.96
15	85.35	86.73
**Average**	84.46	86.04
**error**	0.38	0.46

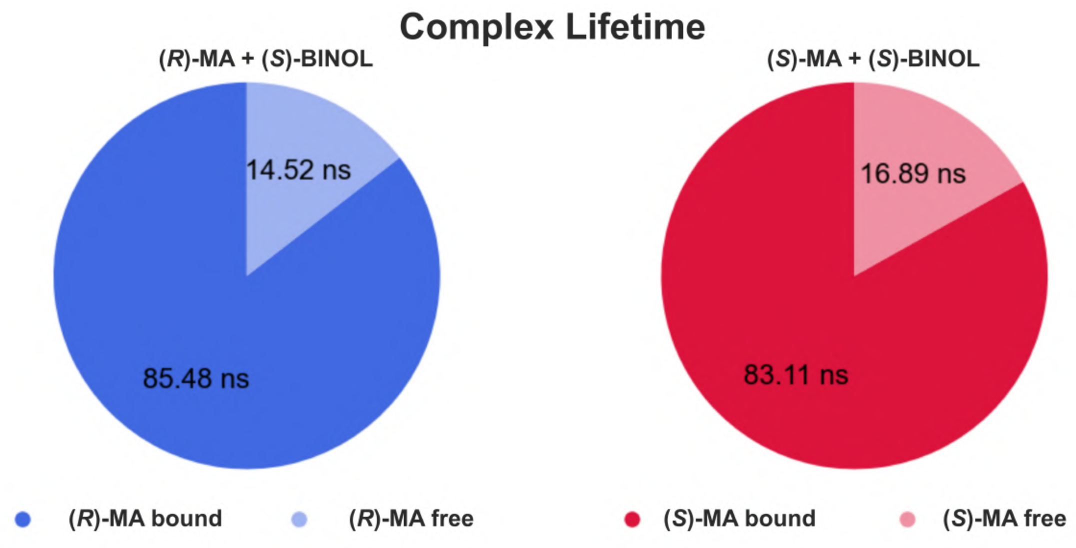

Figure S29: Distribution of complex lifetimes for (*R*)-MA (left) and (*S*)-MA (right) in either the bound or free state with respect to (*S*)-BINOL.

Table S30: Complex lifetimes (in ns) for the diastereoisomeric complexes formed between (*S*)-BINOL and (*R*)-MA, and (*S*)-BINOL and (*S*)-MA, for each of the 15 MD replicates.

**Replicate**	**Complex Lifetime**	
	**(*R*)-MA + (*S*)-BINOL**	**(*S*)-MA + (*S*)-BINOL**
01	86.21	83.48
02	85.37	82.56
03	85.79	84.85
04	84.24	80.18
05	86.87	84.23
06	84.62	84.02
07	86.38	84.21
08	86.61	84.35
09	83.63	80.00
10	87.65	83.28
11	86.10	85.32
12	83.22	82.98
13	82.48	80.13
14	86.17	81.38
15	86.87	85.61
**Average**	85.48	83.11
**error**	0.39	0.49

4.6 Calculating the Stoichiometries of Diastereomeric Complexes

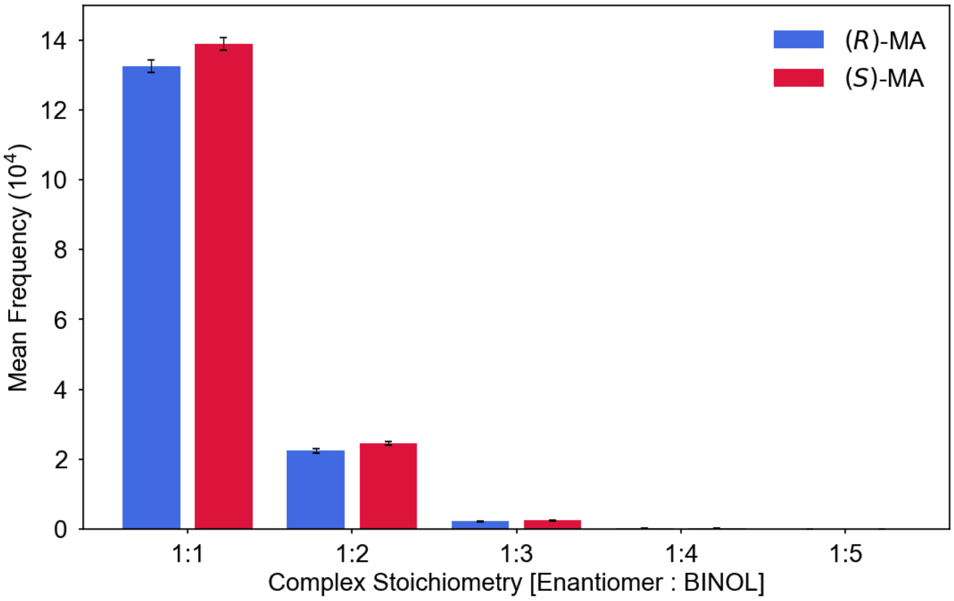

Figure S30: Mean occurrence frequencies of complexes with different stoichiometries for the interactions between (*R*)-BINOL and (*R*)-MA (blue) and (*R*)-BINOL and (*S*)-MA (red).

Table S31: Number of complexes (frequency of occurrence) for each enantiomer in different stoichiometries (MA:(*R*)-BINOL) across each independent molecular dynamics replicate.

Replicate	MA 1:1 BIN		MA 1:2 BIN		MA 1:3 BIN		MA 1:4 BIN		MA 1:5 BIN	
	**RMA**	**SMA**	**RMA**	**SMA**	**RMA**	**SMA**	**RMA**	**SMA**	**RMA**	**SMA**
01	140,167	133,617	19,885	21,685	1,250	1,783	44	105	0	0
02	146,563	131,970	26,531	22,361	2,910	1,962	131	97	1	0
03	124,802	133,070	17,981	27,270	1,590	3,671	40	339	0	8
04	131,596	149,614	24,314	28,469	3,279	3,066	169	167	0	0
05	132,255	140,224	24,101	24,727	2,970	2,495	145	185	0	0
06	122,759	144,297	19,951	27,314	2,439	2,489	199	58	4	0
07	136,799	139,934	19,005	23,922	1,235	2,737	54	284	1	3
08	137,225	132,502	23,642	21,779	1,389	2,015	81	100	1	1
09	136,246	153,437	26,342	25,270	2,925	2,377	72	71	2	4
10	134,849	137,714	22,186	22,787	2,189	2,192	137	110	5	23
11	126,552	131,511	23,756	25,715	3,054	1,837	97	56	0	1
12	124,154	136,434	21,629	22,924	1,862	2,515	51	215	0	20
13	126,205	134,234	22,916	23,875	2,424	2,330	354	73	21	0
14	138,092	148,369	21,404	26,829	1,360	2,406	44	88	0	0
15	130,372	138,152	22,347	23,764	2,094	2,735	185	142	3	0
**Average**	132,576	139,005	22,399	24,579	2,198	2,441	120	139	3	4
**error**	1,762	1,803	647	558	187	127	22	22	1	2

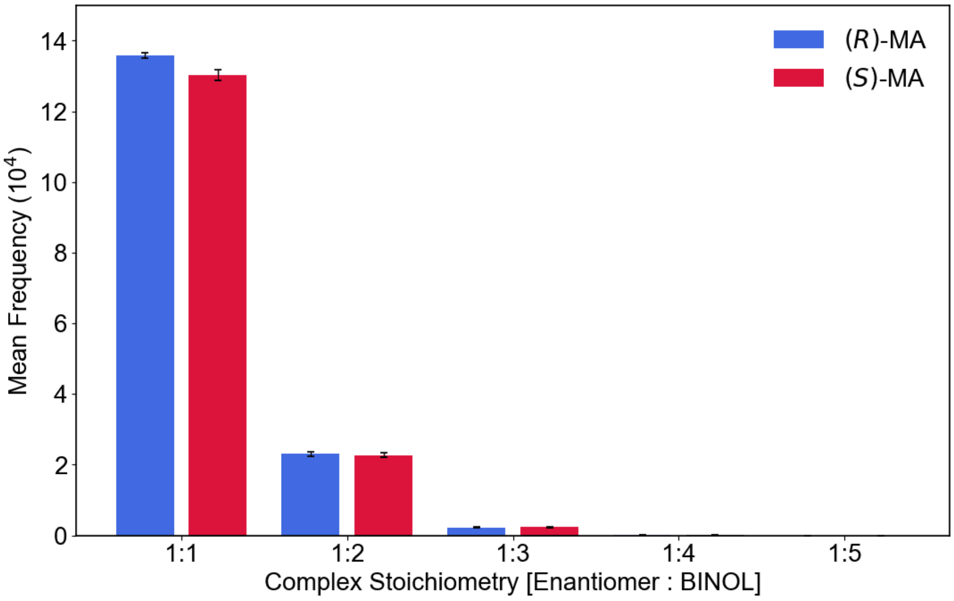

Figure S31: Mean occurrence frequencies of complexes with different stoichiometries for the interactions between (*S*)-BINOL and (*R*)-MA (blue) and (*S*)-BINOL and (*S*)-MA (red).

Table S32: Number of complexes (frequency of occurrence) for each enantiomer in different stoichiometries (MA:(*S*)-BINOL) across each independent molecular dynamics replicate.

Replicate	MA 1:1 BIN		MA 1:2 BIN		MA 1:3 BIN		MA 1:4 BIN		MA 1:5 BIN	
	RMA	SMA	RMA	SMA	RMA	SMA	RMA	SMA	RMA	SMA
01	133,757	138,506	21,669	23,199	2,454	2,488	366	150	30	0
02	134,275	118,902	18,842	19,822	1,897	1,461	91	32	0	0
03	139,101	130,571	23,925	24,479	1,812	3,247	143	214	6	0
04	137,491	127,749	26,670	18,789	2,262	1,236	92	15	3	0
05	136,518	133,693	23,578	25,019	2,096	2,978	250	267	17	5
06	137,014	130,911	23,013	22,517	2,306	1,491	164	18	8	0
07	135,226	135,447	25,725	21,201	2,272	2,423	134	205	0	0
08	139,429	131,022	24,500	27,209	2,471	2,818	239	212	6	0
09	129,789	122,356	18,650	21,818	2,328	2,008	52	271	0	2
10	134,045	134,863	24,951	23,441	2,748	2,958	114	199	1	5
11	136,807	130,840	23,720	24,754	1,495	3,094	48	48	0	0
12	130,093	135,702	19,223	19,801	1,105	2,089	4	217	0	1
13	134,507	119,770	21,852	21,709	2,705	2,072	92	149	1	1
14	139,894	126,386	23,129	21,625	2,101	2,596	232	227	5	6
15	139,823	137,567	26,883	24,811	2,751	2,316	202	150	2	6
**Average**	135,851	130,286	23,089	22,680	2,187	2,352	148	158	5	2
**error**	826	1,596	680	600	119	161	25	23	2	1

4.7 Spatial Distribution Functions

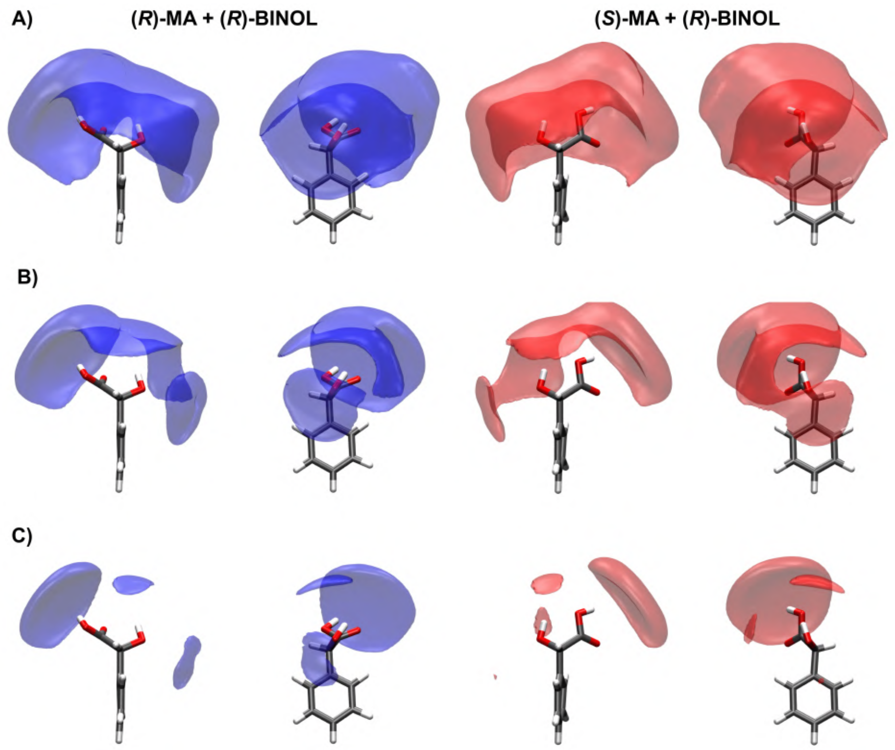

Figure S32: Spatial Distribution Function (SDF) of (*R*)-BINOL around (*R*)-MA and (*S*)-MA at different isovalues. **A)**, **B)**, and **C)** correspond to isovalues of 0.30, 0.40, and 0.50, respectively. For both enantiomers, the front and side views are presented to highlight the distribution patterns.

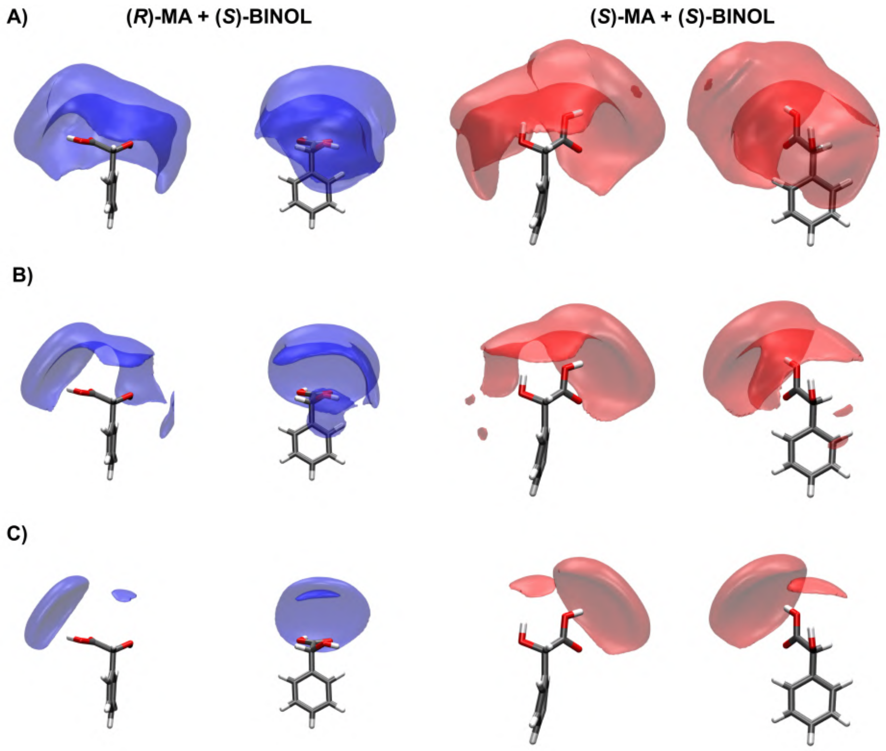

Figure S33: Spatial Distribution Function (SDF) of (*S*)-BINOL around (*R*)-MA and (*S*)-MA at different isovalues. **A)**, **B)**, and **C)** correspond to isovalues of 0.30, 0.40, and 0.50, respectively. For both enantiomers, the front and side views are presented to highlight the distribution patterns.

5 Data from Quantum Chemical Calculations

5.1 Selection of MD Frames and QM Refinement

Table S33: Selected distances (in Å), chosen MA enantiomer, number of structures from MD simulations, and number of remaining complexes at the end of each CREST and CENSO stage, when (*R*)-BINOL is used as the resolving agent. In PART2, the structures were initially optimized at r2SCAN-3c/def2-mTZVPP and then fully optimized at PW6B95-D3/def2-TZVPP. PART3 and PART4 were conducted at PW6B95-D3/def2-TZVPD.

Molecular Dynamics			CREST			CENSO				
**Distance**	**Enantiomer**	**Structures**	**Reopt**	**SPH**	**Cluster**	**PART0**	**PART1**	**PART2**	**PART3**	**PART4**
2.50	*R*	5	2	2	2	8	8	7	6	6
2.95	*R*	24	2	2	2					
14.00	*R*	425	8	7	4					
15.15	*R*	124	2	2	2					
4.75	*S*	849	6	6	4	25	21	16	16	16
7.45	*S*	2098	19	18	5					
8.20	*S*	2030	22	20	5					
10.35	*S*	1811	74	45	7					
14.80	*S*	176	9	5	4					
15.05	*S*	139	2	2	2					

Table S34: Selected distances (in Å), chosen MA enantiomer, number of structures from MD simulations, and number of remaining complexes at the end of each CREST and CENSO stage, when (*S*)-BINOL is used as the resolving agent. In PART2, the structures were initially optimized at r2SCAN-3c/def2-mTZVPP and then fully optimized at PW6B95-D3/def2-TZVPP. PART3 and PART4 were conducted at PW6B95-D3/def2-TZVPD.

**Molecular Dynamics**			**CREST Filtering**			**CENSO Refinement**				
**Distance**	**Enantiomer**	**Structures**	**Reopt**	**SPH**	**Cluster**	**PART0**	**PART1**	**PART2**	**PART3**	**PART4**
2.45	*S*	1	1	1	1					
2.95	*S*	26	8	8	5	5	5	5	5	5
16.10	*S*	27	7	7	5					
4.75	*R*	1124	18	16	2					
5.05	*R*	1297	14	14	4					
9.55	*R*	2298	5	4	3					
10.45	*R*	2418	73	43	4	20	15	11	11	11
13.00	*R*	1109	17	11	7					
15.05	*R*	168	3	2	2					

5.2 Computed Gibbs Free Energies of Binding

Table S35: Calculated Gibbs free energies, binding Gibbs free energies, and the differences between the binding Gibbs free energies. These values are based on Boltzmann weights, expressed in Hartree, and were calculated at the PW6B95-D3/def2-TZVPD level using structures optimized at r2SCAN-3c/def2-mTZVPP.

**Complex**	$G_{\mathbf{tot}}^{\mathbf{COMPLEX}}$	$G_{\mathbf{tot}}^{\mathbf{BINOL}}$	$G_{\mathbf{tot}}^{\mathbf{MA}}$	$\Delta G_{\mathrm{tot}}^{BINDING}$	$\delta\Delta G_{\mathrm{tot}}^{BINDING}$
(*R*)-BINOL + (*R*)-MA	-1458.5279215	-922.3712999	-536.1214564	-0.0351652	0.0023651
(*R*)-BINOL + (*S*)-MA	-1458.5306546	-922.3707555	-536.1223688	-0.0375303	
(*S*)-BINOL + (*R*)-MA	-1458.5307354	-922.3708993	-536.1223751	-0.0374610	-0.0029462
(*S*)-BINOL + (*S*)-MA	-1458.5291118	-922.3712271	-536.1233699	-0.0345147	

Table S36: Calculated Gibbs free energies, binding Gibbs free energies, and the differences between the binding Gibbs free energies. These values are based on Boltzmann weights, expressed in Hartree, and were calculated at the ωB97X-V/def2-TZVPD level using structures optimized at ωB97X-V/def2-TZVPP.

**Complex**	$G_{\mathbf{tot}}^{\mathbf{COMPLEX}}$	$G_{\mathbf{tot}}^{\mathbf{BINOL}}$	$G_{\mathbf{tot}}^{\mathbf{MA}}$	$\Delta G_{\mathrm{tot}}^{BINDING}$	$\delta\Delta G_{\mathrm{tot}}^{BINDING}$
(*R*)-BINOL + (*R*)-MA	-1456.2202554	-920.8862518	-535.2954575	-0.0385462	0.0004879
(*R*)-BINOL + (*S*)-MA	-1456.2228490	-920.8856846	-535.2981303	-0.0390341	
(*S*)-BINOL + (*R*)-MA	-1456.2233252	-920.8859899	-535.2981276	-0.0392077	-0.0030751
(*S*)-BINOL + (*S*)-MA	-1456.2211156	-920.8862969	-535.2986861	-0.0361326	

Table S37: Calculated Gibbs free energies, binding Gibbs free energies, and the differences between the binding Gibbs free energies. These values are based on Boltzmann weights, expressed in Hartree, and were calculated at the PW6B95-D3/def2-TZVPD level using structures optimized at PW6B95-D3/def2-TZVPP.

Complex	$G_{\mathbf{tot}}^{\mathbf{COMPLEX}}$	$G_{\mathbf{tot}}^{\mathbf{BINOL}}$	$G_{\mathbf{tot}}^{\mathbf{MA}}$	$\Delta G_{\mathrm{tot}}^{BINDING}$	$\delta\Delta G_{\mathrm{tot}}^{BINDING}$
(*R*)-BINOL + (*R*)-MA	-1458.5327028	-922.3739091	-536.1239402	-0.0348534	0.0022201
(*R*)-BINOL + (*S*)-MA	-1458.5346313	-922.3731554	-536.1244023	-0.0370736	
(*S*)-BINOL + (*R*)-MA	-1458.5347492	-922.3733996	-536.1245758	-0.0367738	-0.0018303
(*S*)-BINOL + (*S*)-MA	-1458.5332512	-922.3736714	-536.1246364	-0.0349435	

5.3 DFT calculation of ^{1}H Chemical Shifts

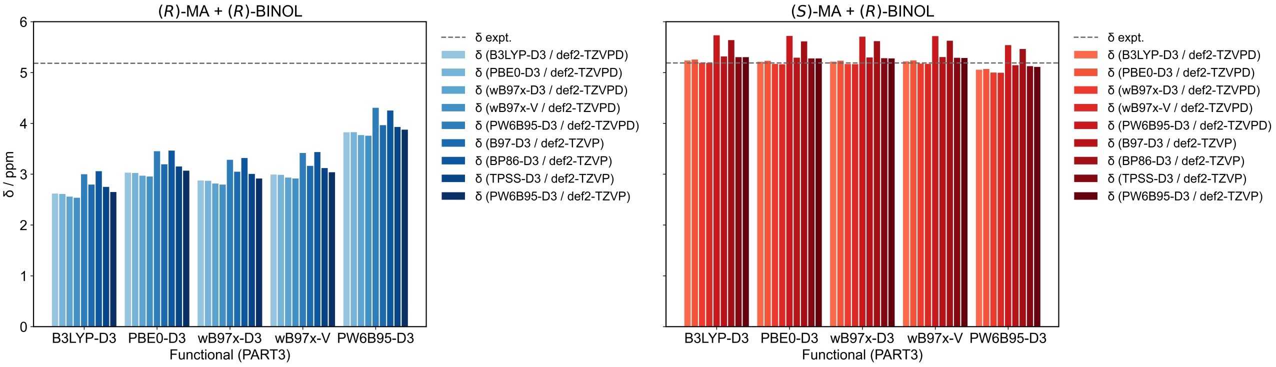

Figure S34: Calculated chemical shifts for the hydrogen atom at the chiral center of (*R*)-MA (left) (*S*)-MA (right) across different theoretical levels, using the SMD solvation model, and in the presence of (*R*)-BINOL. Structures were optimized at the r2SCAN-3c/def2-mTZVPP level of theory, and the functionals in PART3 were used with the def2-TZVPD basis set.

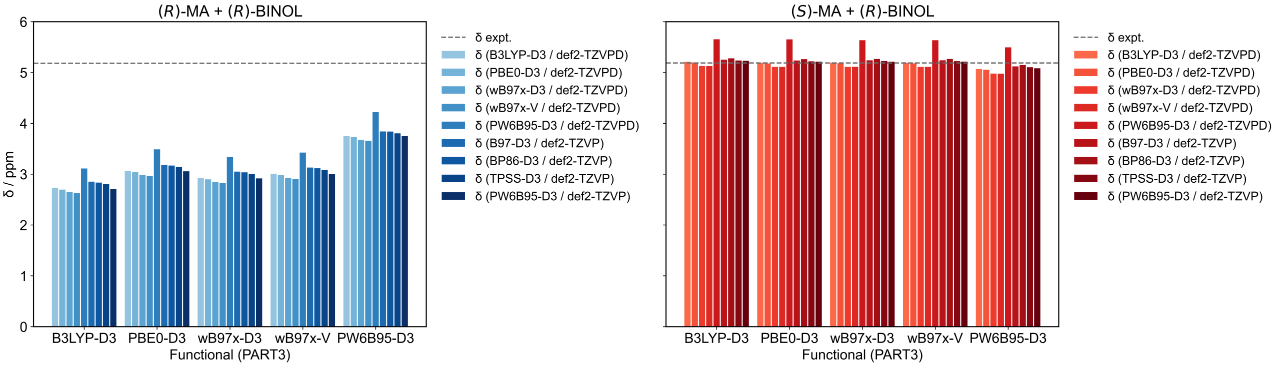

Figure S35: Calculated chemical shifts for the hydrogen atom at the chiral center of (*R*)-MA (left) (*S*)-MA (right) across different theoretical levels, using the CPCM solvation model, and in the presence of (*R*)-BINOL. Structures were optimized at the r2SCAN-3c/def2-mTZVPP level of theory, and the functionals in PART3 were used with the def2-TZVPD basis set.

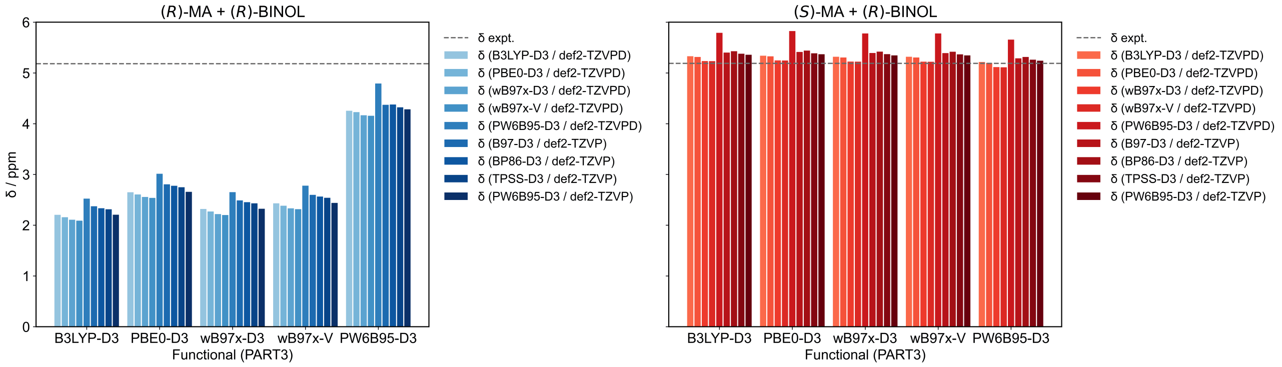

Figure S36: Calculated chemical shifts for the hydrogen atom at the chiral center of (*R*)-MA (left) (*S*)-MA (right) across different theoretical levels, using the SMD solvation model, and in the presence of (*R*)-BINOL. Structures were pre-optimized at the r2SCAN-3c/def2-mTZVPP and fully optimized at the PW6B95-D3/def2-TZVPP level of theory. The functionals in PART3 were used with the def2-TZVPD basis set.

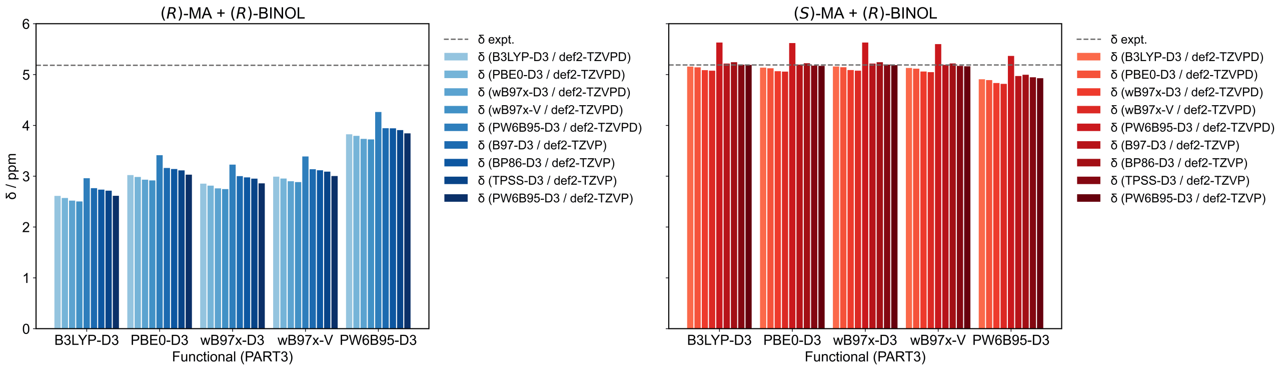

Figure S37: Calculated chemical shifts for the hydrogen atom at the chiral center of (*R*)-MA (left) (*S*)-MA (right) across different theoretical levels, using the SMD solvation model, and in the presence of (*R*)-BINOL. Structures were pre-optimized at the r2SCAN-3c/def2-mTZVPP and fully optimized at the ωB97X-V/def2-TZVPP level of theory. The functionals in PART3 were used with the def2-TZVPD basis set.

5.4 Calculating Solvation Energy

Solvation energies (ΔE_{solv}) and the relative differences ($\Delta\Delta E_{\text{solv}}$) between the solvation energies for the formation of homochiral and heterochiral complexes were calculated. These values were obtained from high-accuracy DFT single-point calculations using the CENSO-PART3 workflow for the fully optimized structures at the PW6B95-D3/def2-TZVPP level of theory. All solvation energies are based on the Boltzmann-weighted populations.

For the COSMO-RS model, solvation energy calculations were performed separately using the same level of theory as CENSO-PART3. These computations were conducted with ORCA 6.0.1, interfaced with the open-source implementation of the COSMO-RS (openCOSMO-RS)[12].

Table S38: Calculated solvation energies (E_{solv}) for individual components, solvation energies of diastereoisomeric complexation ($\Delta E_{\text{solv}}^{RR,SR}$), and their relative differences ($\delta\Delta E_{\text{solv}}$) using various solvation models. All values are reported in kcal / mol.

**Solvation Model**	$E_{\textbf{solv}}^{\textbf{(}R\textbf{)-MA}}$	$E_{\textbf{solv}}^{\textbf{BINOL}}$	$E_{\textbf{solv}}^{\textbf{COMPLEX}}$	$\Delta E_{\text{solv}}^{RR}$	$E_{\textbf{solv}}^{\textbf{(}S\textbf{)-MA}}$	$E_{\textbf{solv}}^{\textbf{BINOL}}$	$E_{\textbf{solv}}^{\textbf{COMPLEX}}$	$\Delta E_{\text{solv}}^{SR}$	$\delta\Delta E_{\text{solv}}$
SMD	-12.401	-16.736	-26.396	2.742	-11.532	-16.869	-23.377	5.024	-2.282
CPCM	-9.389	-7.431	-15.047	1.773	-8.641	-7.577	-12.263	3.955	-2.182
COSMO-RS	-11.199	-16.182	-27.595	-0.214	-10.781	-16.277	-26.130	0.928	-1.142

6 References

[1] Cabral, T. L. G.; Dal Poggetto, G.; Brussolo da Silva, J. P.; Nilsson, M.; Tormena, C. F. Determining the Absolute Configuration of Small Molecules by Diffusion NMR Experiments. *Angew. Chem. Int. Ed.* **2025**, *64*, e202418508.

[2] Drosg, M. *Dealing with Uncertainties: A Guide to Error Analysis*, 2nd ed.; Springer Berlin, Heidelberg, 2009; eBook ISBN: 978-3-642-01384-3.

[3] Pelta, M. D.; Morris, G. A.; Stchedroff, M. J.; Hammond, S. J. A one-shot sequence for high-resolution diffusion-ordered spectroscopy. *Magn. Reson. Chem.* **2002**, *40*, S147–S152.

[4] Wagner, R.; Berger, S. Gradient-selected NOESY-A fourfold reduction of the measurement time for the NOESY experiment. *J. Magn. Reson. Ser. A.* **1996**, *123*, 119–121.

[5] Jeener, J.; Meier, B.; Bachmann, P.; Ernst, R. R. Investigation of exchange processes by two-dimensional NMR spectroscopy. *J. Chem. Phys.* **1979**, *71*, 4546–4553.

[6] Dodda, L. S.; Cabeza de Vaca, I.; Tirado-Rives, J.; Jorgensen, W. L. LigParGen web server: an automatic OPLS-AA parameter generator for organic ligands. *Nucleic Acids Res.* **2017**, *45*, W331–W336.

[7] Dodda, L. S.; Vilseck, J. Z.; Tirado-Rives, J.; Jorgensen, W. L. 1.14* CM1A-LBCC: localized bond-charge corrected CM1A charges for condensed-phase simulations. *J. Phys. Chem. B.* **2017**, *121*, 3864–3870.

[8] Pranami, G.; Lamm, M. H. Estimating error in diffusion coefficients derived from molecular dynamics simulations. *J. Chem. Theory Comput.* **2015**, *11*, 4586–4592.

[9] Peluso, P.; Chankvetadze, B. Recognition in the domain of molecular chirality: from noncovalent interactions to separation of enantiomers. *Chem. Rev.* **2022**, *122*, 13235–13400.

[10] Salome, K. S.; Tormena, C. F. Enantiodiscrimination by matrix-assisted DOSY NMR. *Chem. Commun.* **2019**, *55*, 8611–8614.

[11] Honegger, P.; Di Pietro, M. E.; Castiglione, F.; Vaccarini, C.; Quant, A.; Steinhauser, O.; Schroder, C.; Mele, A. The intermolecular NOE depends on isotope selection: Short range vs long range behavior. *J. Phys. Chem. Lett.* **2021**, *12*, 8658–8663.

[12] Müller, S.; Nevolianis, T.; Garcia-Ratés, M.; Riplinger, C.; Leonhard, K.; Smirnova, I. Predicting solvation free energies for neutral molecules in any solvent with openCOSMO-RS. *Fluid Ph. Equilib.* **2025**, *589*, 114250.
